# Functional omics of ORP7 in primary endothelial cells

**DOI:** 10.1186/s12915-024-02087-6

**Published:** 2024-12-18

**Authors:** Juuso H. Taskinen, Minna Holopainen, Hanna Ruhanen, Miesje van der Stoel, Reijo Käkelä, Elina Ikonen, Salla Keskitalo, Markku Varjosalo, Vesa M. Olkkonen

**Affiliations:** 1 Minerva Foundation Institute for Medical Research, Tukholmankatu 8, 00290 Helsinki, Finland; 2 Molecular and Integrative Biosciences Research Programme, Faculty of Biological and Environmental Sciences, Helsinki University Lipidomics Unit (HiLIPID), Helsinki Institute of Life Science (HiLIFE) and Biocenter Finland, University of Helsinki, Viikinkaari 1, PO BOX 65, 00014 Helsinki, Finland; 3 Department of Anatomy and Stem Cells and Metabolism Research Program, Faculty of Medicine, University of Helsinki, Haartmaninkatu 8, 00290 Helsinki, Finland; 4 Proteomics Unit Viikki, Institute of Biotechnology, HiLIFE and Biocenter Finland, University of Helsinki, Viikinkaari 1, 00790 Helsinki, Finland; 5 Systems Biology/Pathology Research Group, iCAN Digital Precision Cancer Medicine Flagship, University of Helsinki, Helsinki, Finland; 6 Department of Anatomy, Faculty of Medicine, University of Helsinki, Haartmaninkatu 8, Helsinki, 00290, Finland

**Keywords:** Transcriptomics, Lipidomics, Interactomics, Endothelial cells, Inflammation, OSBPL7, ORP7, AKT1

## Abstract

**Background:**

Many members of the oxysterol-binding protein-related protein (ORP) family have been characterized in detail over the past decades, but the lipid transport and other functions of ORP7 still remain elusive. What is known about ORP7 points toward an endoplasmic reticulum and plasma membrane-localized protein, which also interacts with GABA type A receptor-associated protein like 2 (GABARAPL2) and unlipidated Microtubule-associated proteins 1A/1B light chain 3B (LC3B), suggesting a further autophagosomal/lysosomal association. Functional roles of ORP7 have been suggested in cholesterol efflux, hypercholesterolemia, and macroautophagy. We performed a hypothesis-free multi-omics analysis of chemical ORP7 inhibition utilizing transcriptomics and lipidomics as well as proximity biotinylation interactomics to characterize ORP7 functions in a primary cell type, human umbilical vein endothelial cells (HUVECs). Moreover, assays on angiogenesis, cholesterol efflux, and lipid droplet quantification were conducted.

**Results:**

Pharmacological inhibition of ORP7 leads to an increase in gene expression related to lipid metabolism and inflammation, while genes associated with cell cycle and cell division were downregulated. Lipidomic analysis revealed increases in ceramides and lysophosphatidylcholines as well as saturated and monounsaturated triacylglycerols. Significant decreases were seen in all cholesteryl ester and in some unsaturated triacylglycerol species, compatible with the detected decrease of mean lipid droplet area. Along with the reduced lipid stores, ATP-binding cassette subfamily G member 1 (ABCG1)-mediated cholesterol efflux and angiogenesis decreased. Interactomics revealed an interaction of ORP7 with AKT1, a central metabolic regulator.

**Conclusions:**

The transcriptomics results suggest an increase in prostanoid as well as oxysterol synthesis, which could be related to the observed upregulation of proinflammatory genes. We envision that the defective angiogenesis in HUVECs subjected to ORP7 inhibition could be the result of an unfavorable plasma membrane lipid composition and/or reduced potential for cell division. To conclude, the present study suggests multifaceted functions of ORP7 in lipid homeostasis, angiogenic tube formation, and gene expression of lipid metabolism, inflammation, and cell cycle in primary endothelial cells.

**Supplementary Information:**

The online version contains supplementary material available at 10.1186/s12915-024-02087-6.

## Background

Current understanding of the oxysterol-binding protein-related protein (ORP) family is extensive, but the functions of some of its members still elude researchers. A good example is ORP7 which has been left with little attention, whereas oxysterol-binding protein (OSBP), from which the family takes its name, has been extensively studied. The primary role of ORPs is to establish membrane contact sites (MCS) where they facilitate the transfer of different lipids between intracellular membranes [1]. A well-studied MCS is formed by OSBP between the ER and *trans*-Golgi, where OSBP mediates the transport of endoplasmic reticulum (ER) cholesterol to *trans*-Golgi and reciprocally trans-Golgi PI(4)P is transported to the ER [2]. Another well-examined family member is ORP2, which carries cholesterol from endosomes to the plasma membrane (PM) in exchange for PI(4,5)P_2_ transported from PM to endosomes [3–5]. Which lipids ORP7 transports and between which membranes remain unclear, as do its other cellular functions The sparse knowledge on ORP7 suggests that it localizes to the PM and ER [6] and interacts with GABARAPL2, formerly GATE16 [7] which has been shown to localize to ER, Golgi, and autophagosomes [8, 9]. More recent studies have unveiled that inhibition of ORP7 leads to an increase in ABCA1-dependent cholesterol efflux to apolipoprotein A1 (ApoA1) in kidney podocytes, while ORP7 inhibition had a weaker impact on cholesterol efflux to high-density lipoprotein (HDL) particles [10]. A whole genome sequencing study in a Malaysian cohort showed that participants with a ORP7 c.651_652del variant had 17 times higher odds of hypercholesterolemia than subjects without the variant [11]. A large-scale study of the human macroautophagy pathway revealed that ORP7 among other ORPs binds to the unlipidated form of LC3B and is required for macroautophagy [12]. A database mining study on pancreatic ductal adenocarcinoma (PDCA) data performed by Chou et al. showed that *OSBPL7* had the highest positive correlation with *OSBPL2* and *OSBPL3* and highest negative correlations in expression with *OSBPL8* and *OSBPL11* [13]. Expression of *OSBPL7* was also shown to be positively associated with the infiltration of immune cells. Moreover, single-nucleotide variations in *OSBPL7* have been associated with eczema [14] as well as with the uptake of amyloid beta in the brain of a Korean cohort [15]. The most recent results have shown that ORP7 silencing in podocytes increases triacylglycerol (TG) levels, membrane fluidity, and lipid droplet numbers as well as activates ER stress and apoptosis. Total cholesterol (TC) levels were shown to be slightly but non significantly increased, and an increase in LC3II/LC3I ratios was shown along with a reduction in GABARAPL2 levels and autophagy [16].

Given the sparse information on ORP7, we set out to investigate how the manipulation of ORP7 affects the macromolecule pools of primary human umbilical vein endothelial cells (HUVECs). To this end, we employed hypothesis-free omics methods such as transcriptomics, lipidomics, and interactomics to get a systemwide view of the changes in manipulated HUVECs. We used a recently discovered 5-arylnicotinamide ORP7 inhibitor CpdG [10] and an ORP7 proximity biotinylation overexpression construct with a mycBirA domain when applying these omics to study HUVECs. To our knowledge, this is to date the first study to thoroughly investigate the effects of ORP7 manipulation by using multiomics in any cell or animal model.

## Results

We performed all, except interactomics, analyses on both CpdG/DMSO treated or ORP7-mycBirA (oexORP7) and mycBirA (oexControl) expressing cells but chose to focus on the results acquired for CpdG-treated cells. Results from the oex cells have been relegated to supplementary material, and addressed only when relevant, in order to keep the present article concise.

### HUVECs exhibit a high tolerance for ORP7 inhibition and overexpression

When inhibitors are used in a new cell model, it is vital to study how they affect cellular health to discover a concentration where the cells show little to no toxicity and the inhibitor shows strong inhibition of its target. To this end, we performed the well-established MTT assay, to measure metabolic activity of HUVECs treated with CpdG or the vehicle control DMSO as well as in oexORP7 and oexControl cells. CpdG was well tolerated by HUVECs: a 50% reduction in metabolic activity was reached at 250 μM concentration, while the third highest concentration of 100 μM displayed similar reductions in metabolic activity to all the lower concentrations of CpdG (Additional file 1: Fig. S1A). oexORP7 cells exhibited a small but statistically significant 33% decrease in the metabolic activity compared to oexControl (Additional file 1: Fig. S1B). We chose to continue further experimentation with 10 μM concentration of CpdG as it did not affect HUVEC metabolism in a deleterious manner, and this concentration has been shown to be biologically relevant in THP1 cells [10].

### Quality control of overexpression, treatment, and omics results

**Fig. 1:**
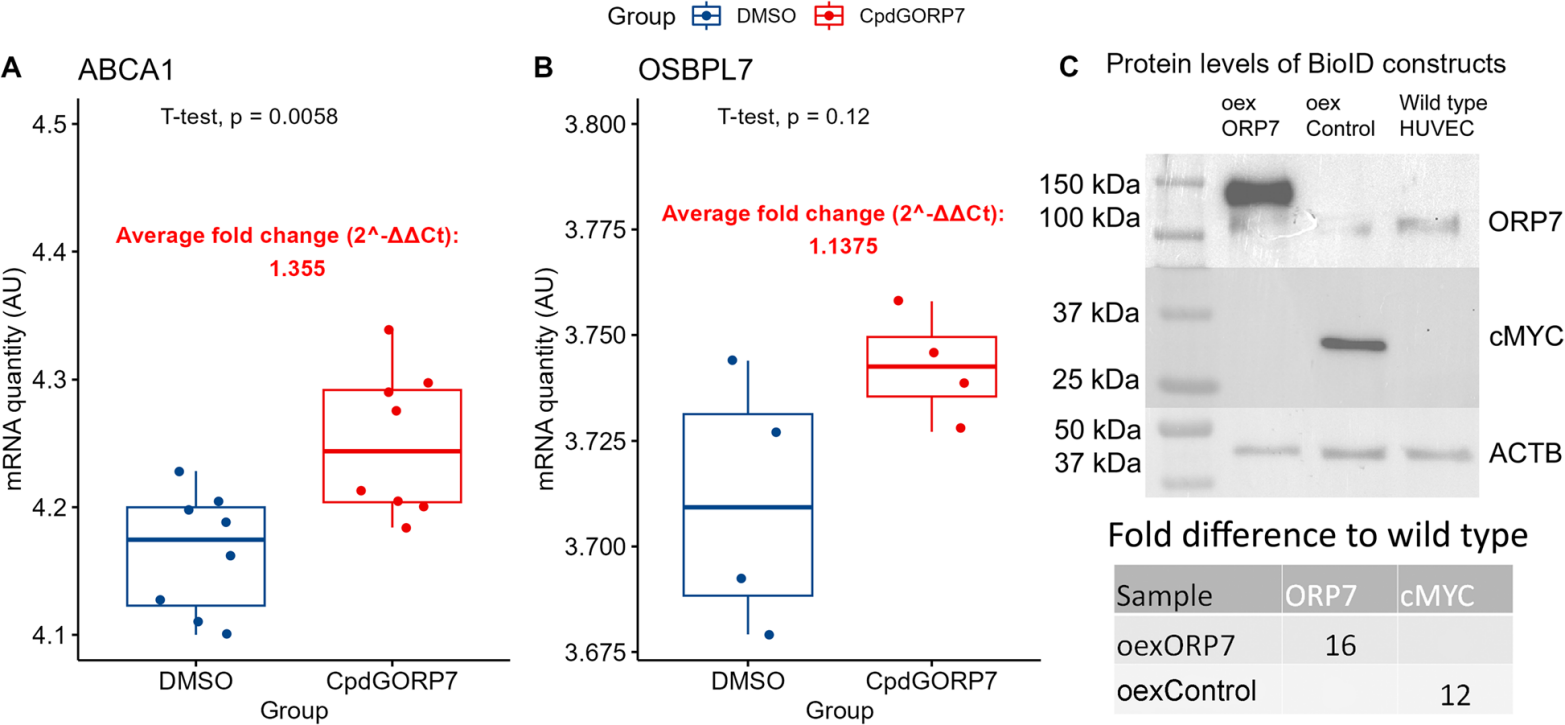
Results of qPCR from CpdG- and DMSO-treated cells and Western blotting of ORP7 or MYC overexpressing cells. **A** mRNA quantity of *ABCA1* in arbitrary units (*N* = 8 per group). **B** mRNA quantity of *OSBPL7* in arbitrary units, showing a statistically non-significant average fold change of 1.13. *N* = 4 per group. **C** Western blotting results of overexpressing cells. The membranes from top to bottom are protein levels of ORP7, cMYC, and β-Actin (ACTB). The leftmost lane is for oexORP7 cells, the second lane displays the same in oexControl cells, and the last lane depicts the same but in wild type cells. Fold differences of proteins compared to wild type shown in the table were calculated from b-Actin normalized pixel densities compared to wild type. Values are from one experiment

To ensure the quality of results from the experiments detailed in further sections, we performed rigorous quality control of both CpdG-treated and oexORP7 cells. We examined the mRNA levels of *ABCA1* and *OSBPL7* in CpdG-treated cells with qPCR, as *ABCA1* expression has been shown to increase with ORP7 inhibition [10]. The CpdG-treated cells had statistically significantly higher ABCA1 expression (fold change 1.36) than the DMSO-treated cells (Fig. 1A), but the parallel slight increase of OSBPL7 was not significant (Fig. 1B). At the protein level, the western blot analysis verified the overexpression of oexORP7 and oexControl in the corresponding transduced cells (Fig. 1C). The level of ORP7-mycBirA overexpression was approximately 16-fold as compared to the endogenous ORP7 in untransduced (wild type) cells.

To ensure the quality of our omics results, we performed principal component analysis (PCA) to verify if our manipulations resulted in variation between sample groups in all omics experiments performed (Additional file 1: Fig. S2 A–D). When using as PCA loadings either transcript counts, lipid concentrations, or for interactome analysis unique spectra counts, in each experiment, all samples resembled the samples of their own treatment group, and treatment or overexpression groups separated along the dimension that has the largest explanatory power (Additional file 1: Fig. S2 A–D). This indicates that the variation between the biochemical profiles of samples was mostly due to the treatments or the overexpression.

### Transcriptomics reveals multifaceted changes in CpdG-treated HUVECs

**Fig. 2:**
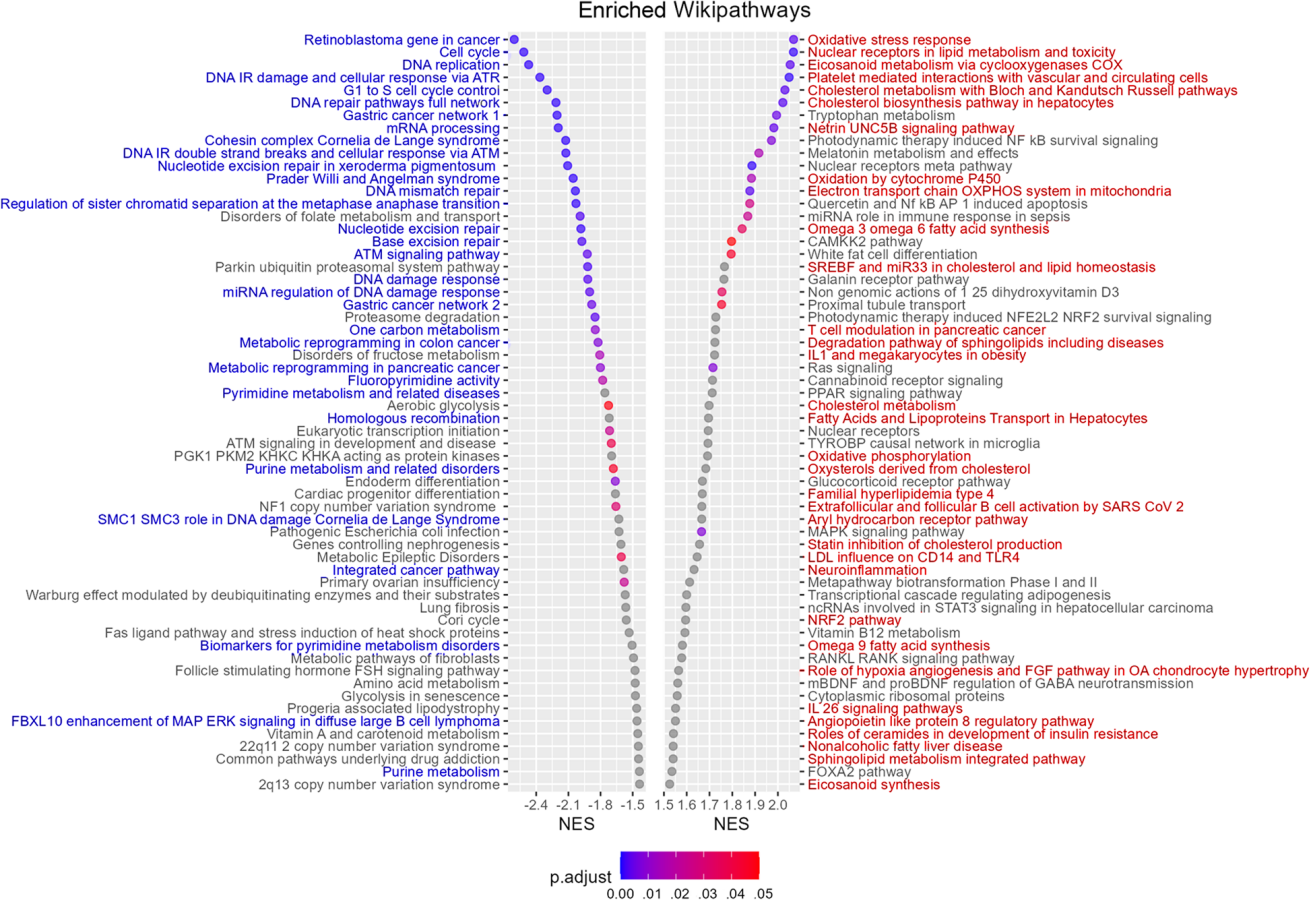
Subset of GSEA results of differentially expressed genes from CpdG-treated cells using WikiPathways database. *Y*-axis represents enriched gene sets, and *X*-axis the normalized enrichment score (NES) for each gene set, where a negative value indicates overrepresentation in highly downregulated and positive values highly upregulated genes. The color of each dot represents the Benjamini–Hochberg adjusted *p*-value for each gene set. Highlighted with blue font are the gene sets related to cell cycle, cell division, cancer, DNA repair, and nucleotide metabolism. Highlighted with red font are the gene sets related to lipid metabolism, mitochondrial function, and inflammation. *N* = 4, values are from one experiment

Transcriptomics is a convenient method to study the intermediate steps from genotype to phenotype. It can give insights into how cells compensate for the effects of a test variable. We performed a comprehensive gene set analysis on CpdG-treated differential expression data compared to DMSO control, in order to reach a better understanding of how HUVECs compensate for the CpdG treatment (Fig. 2). WikiPathways gene sets with overrepresentation of downregulated genes were related to cell cycle, DNA repair, cancer, and nucleic acid metabolism (highlighted with blue in the left facet of Fig. 2). Overrepresentation in upregulated genes was mostly related to lipid metabolism gene sets, such as cholesterol biosynthesis, eicosanoid metabolism, synthesis of n-3 and n-6 polyunsaturated fatty acids, and oxysterols derived from cholesterol. Inflammation-related sets were also overrepresented, which include sets such as IL-18 signaling pathway, platelet-mediated interactions, and netrin-UNC5B signaling pathway. Moreover, gene set related to mitochondria, such as oxidative stress response, electron transport chain OXPHOS system in mitochondria, mitochondrial complex 1 assembly model OXPHOS system, and NRF2 pathway (highlighted with red in the right facet of Fig. 2) also stood out. Similar analysis done on other databases and on data from overexpressing cells is shown in Additional file 2: Figs. S1–S16.

**Fig. 3:**
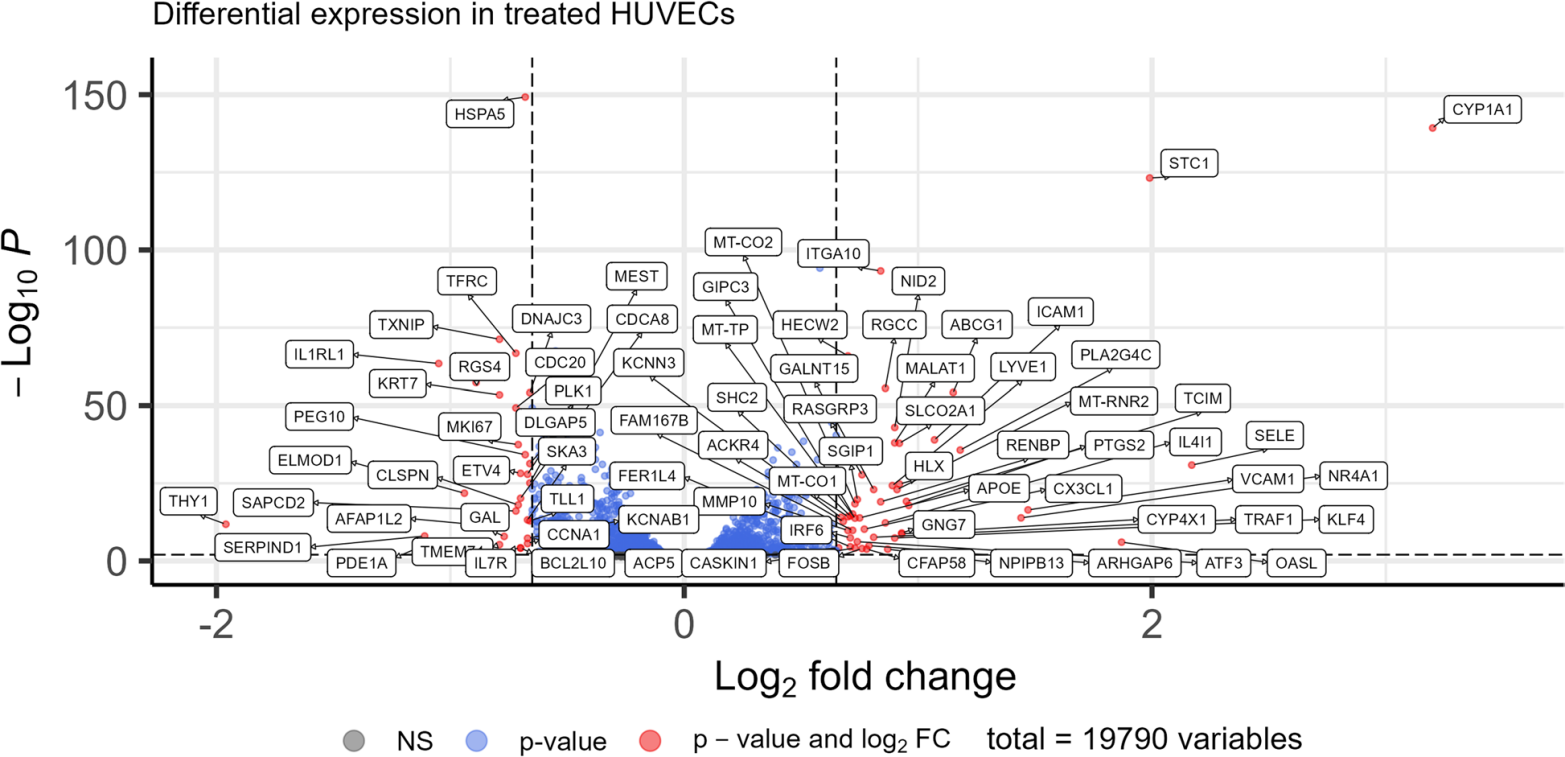
Volcano plot of differential gene expression in CpdG-treated cells compared to DMSO control. *X*-axis represents log2 fold change and *Y*-axis -log10 adjusted *p*-value. Annotated red dots show genes with an absolute log2 fold change of 0.75 or higher and are significantly altered, blue dots have an absolute log2 fold change lower than 0.75 and are significantly altered, and grey dots below and between dashed lines represent genes that have not been significantly altered

Gene set analysis is a convenient and robust way to produce systematic understanding of the basis of functional changes, but understanding gene level changes provides a more detailed and precise understanding into a differential expression dataset. Examining the changes in individual genes can reveal variations in, for example, key pathway genes or rate limiting enzymes that affect whole pathways. The same goes for transcriptional factors which, when differentially expressed, can have significant downstream effects. Therefore, we also plotted the differentially expressed genes as a conventional volcano plot (Fig. 3).

Interestingly, many genes related to prostaglandin metabolism were upregulated. These included *CYP1A1*, *SLOC2A1*, and *PTGS2* [17–19] as well as phospholipase, lysophospholipase, and O-acyltransferase PLA2G4C, which release arachidonic acid (AA) from phosphatidylcholine [20–22]. Other notable upregulated genes were *SELE*, *VCAM1*, and *ICAM1*, which are related to endothelial cell inflammation and early atherogenesis [23–26]. A similar expression pattern is visible in other inflammation and atherosclerosis related genes such as *APOE*, *KLF4*, *CXC3L1*, *STC1*, *CH25H*, and *ABCG1* [27–36]. Downregulation was evident in inflammation related genes such as *THY1*, *IL1RL1*, and *IL7R* [37, 38] as well as in cell adhesion related genes including *FERMT1* [39]. Log2 fold change and statistics for the aforementioned genes and other genes related to, for example cholesterol synthesis, autophagy and inflammation can be found in Table 1.

**Table 1:** Differential expression metrics for selected genes related to lipid metabolism, cholesterol metabolism, inflammation, mammalian autophagy [40] and endothelial cell function. Log2 fold change (log2FC) and Benjamini–Hochberg adjusted *p*-values (padj) are shown

ENTREZID	SYMBOL	GENENAME	log2FC	padj
19	ABCA1	ATP-binding cassette subfamily A member 1	0.16	0.005
9619	ABCG1	ATP-binding cassette subfamily G member 1	1.17	8.64E − 59
239	ALOX12	Arachidonate 12-lipoxygenase, 12S type	0.001	0.993
240	ALOX5	Arachidonate 5-lipoxygenase	0.01	NA
241	ALOX5AP	Arachidonate 5-lipoxygenase-activating protein	0.01	NA
59,344	ALOXE3	Arachidonate lipoxygenase 3	− 0.02	NA
348	APOE	Apolipoprotein E	0.79	3.25E − 16
83,734	ATG10	Autophagy-related 10	− 0.02	0.826
60,673	ATG101	Autophagy-related 101	− 0.12	0.070
9140	ATG12	Autophagy-related 12	0.08	0.187
9776	ATG13	Autophagy-related 13	− 0.02	0.754
22,863	ATG14	Autophagy-related 14	0.07	0.418
55,054	ATG16L1	Autophagy-related 16 like 1	0.05	0.538
89,849	ATG16L2	Autophagy-related 16 like 2	0.58	2.26E − 06
64,422	ATG3	Autophagy-related 3	0.002	0.981
115,201	ATG4A	Autophagy-related 4A cysteine peptidase	− 0.12	0.050
23,192	ATG4B	Autophagy-related 4B cysteine peptidase	0.06	0.354
84,938	ATG4C	Autophagy-related 4C cysteine peptidase	− 0.02	0.816
84,971	ATG4D	Autophagy-related 4D cysteine peptidase	0.07	0.477
9474	ATG5	Autophagy-related 5	− 0.05	0.581
10,533	ATG7	Autophagy-related 7	− 0.07	0.344
1071	CETP	Cholesteryl ester transfer protein	0.37	0.024
9023	CH25H	Cholesterol 25-hydroxylase	0.94	2.92E − 05
54,206	CYCS	Cytochrome c, somatic	− 0,26	5.27E10
1543	CYP1A1	Cytochrome P450 family 1 subfamily A member 1	3.23	2.04E − 148
1593	CYP27A1	Cytochrome P450 family 27 subfamily A member 1	0.41	6.61E − 11
1718	DHCR24	24-Dehydrocholesterol reductase	− 0.18	1.11E − 05
1717	DHCR7	7-Dehydrocholesterol reductase	0.06	0.371
3992	FADS1	Fatty acid desaturase 1	0.23	1.36E − 06
9415	FADS2	Fatty acid desaturase 2	0.30	9.73E − 13
2222	FDFT1	Farnesyl-diphosphate farnesyltransferase 1	0.12	0.012
2224	FDPS	Farnesyl diphosphate synthase	− 0.03	0.738
55,612	FERMT1	FERM domain containing kindlin 1	− 0.97	0.0001
2321	FLT1	fms-related receptor tyrosine kinase 1	− 0.15	4.50E − 05
11,337	GABARAP	GABA type A receptor-associated protein	0.003	NA
23,710	GABARAPL1	GABA type A receptor-associated protein like 1	0.25	5.81E − 07
11,345	GABARAPL2	GABA type A receptor-associated protein like 2	0.04	0.551
9453	GGPS1	Geranylgeranyl diphosphate synthase 1	− 0.03	0.742
3156	HMGCR	3-Hydroxy-3-methylglutaryl-CoA reductase	− 0.03	0.664
3157	HMGCS1	3-Hydroxy-3-methylglutaryl-CoA synthase 1	0.05	0.541
80,270	HSD3B7	Hydroxy-delta-5-steroid dehydrogenase, 3 beta- and steroid delta-isomerase 7	0.10	0.296
3383	ICAM1	Intercellular adhesion molecule 1	1.10	5.41E − 43
9173	IL1RL1	Interleukin 1 receptor like 1	− 1.07	5.36E − 68
3575	IL7R	Interleukin 7 receptor	− 0.91	1.21E − 05
9314	KLF4	KLF transcription factor 4	1.06	3.82E − 09
3949	LDLR	Low density lipoprotein receptor	0.02	0.864
3988	LIPA	Lipase A, lysosomal acid type	0.16	0.001
4047	LSS	Lanosterol synthase	0.10	0.031
84,557	MAP1LC3A	Microtubule-associated protein 1 light chain 3 alpha	0.41	1.55E − 11
81,631	MAP1LC3B	Microtubule-associated protein 1 light chain 3 beta	0.23	5.55E − 07
643,246	MAP1LC3B2	Microtubule-associated protein 1 light chain 3 beta 2	− 0.02	NA
4597	MVD	Mevalonate diphosphate decarboxylase	0.10	0.121
4598	MVK	Mevalonate kinase	0.06	0.473
4864	NPC1	NPC intracellular cholesterol transporter 1	0.16	0.005
10,577	NPC2	NPC intracellular cholesterol transporter 2	0.31	3.49E − 15
7376	NR1H2	Nuclear receptor subfamily 1 group H member 2	0.06	0.286
10,062	NR1H3	Nuclear receptor subfamily 1 group H member 3	0.27	0.001
57,110	PLAAT1	Phospholipase A and acyltransferase 1	0.74	0.0001
10,654	PMVK	Phosphomevalonate kinase	− 0.04	0.575
5742	PTGS1	Prostaglandin-endoperoxide synthase 1	0.44	5.67E − 19
5743	PTGS2	Prostaglandin-endoperoxide synthase 2	1.02	1.67E − 20
9821	RB1CC1	RB1-inducible coiled-coil 1	0.004	0.963
949	SCARB1	Scavenger receptor class B member 1	− 0.22	8.44E − 07
6319	SCD	Stearoyl-CoA desaturase	0.46	1.09E − 34
6401	SELE	Selectin E	2.19	1.39E − 34
6646	SOAT1	Sterol O-acyltransferase 1	0.06	0.415
6713	SQLE	Squalene epoxidase	− 0.11	0.064
6720	SREBF1	Sterol regulatory element-binding transcription factor 1	0.45	4.18E − 22
6781	STC1	Stanniocalcin 1	1.99	7.73E − 133
8408	ULK1	unc-51 like autophagy activating kinase 1	0.03	0.632
9706	ULK2	unc-51 like autophagy activating kinase 2	0.19	0.003
7412	VCAM1	Vascular cell adhesion molecule 1	1.52	2.84E − 19
7422	VEGFA	Vascular endothelial growth factor A	0.20	0.011
7423	VEGFB	Vascular endothelial growth factor B	0.29	5.68E − 09
55,062	WIPI1	WD repeat domain, phosphoinositide-interacting 1	0.06	0.456
26,100	WIPI2	WD repeat domain, phosphoinositide-interacting 2	− 0.01	0.905

### Manipulated HUVECs have reduced angiogenic capacity

**Fig. 4:**
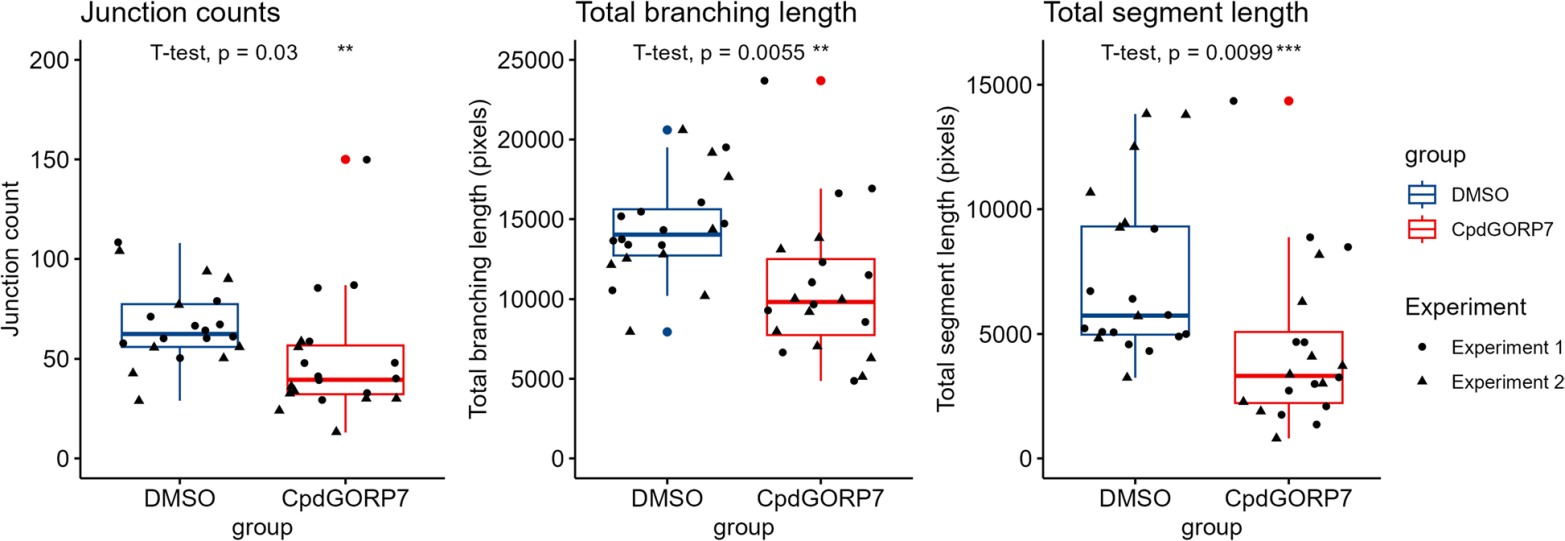
Angiogenic metrics from CpdG or DMSO treated cells grown on angiogenesis inducing matrix. In all box plots, the *Y*-axis represents each angiogenic metric and *X*-axis the group measured. Blue represents the DMSO control and red indicates CpdG-treated cells; length is represented in pixels. As shown, slight reductions in all angiogenetic metrics CpdG treated cells are clearly visible. *N* = 20 per group. Values are from two independent experiments, which have been delineated with dots and triangles, and outliers are highlighted as colored dots

One of the crucial functions of endothelial cells is angiogenesis, i.e., the formation of new blood vessels from existing ones [41]. We used a matrix-based angiogenesis assay to measure how CpdG treatment affects angiogenesis in HUVECs. Inhibition of ORP7 reduced angiogenetic capacity in all metrics measured but did not block it entirely. The number of junctions, branching length, and segment length of the newly formed vessels were all statistically significantly decreased in the CpdG-treated cells compared to the DMSO controls (Fig. 4). The definitions of each angiogenic metric are detailed in the documentation of Angiogenesis Analyzer [42].

### Lipidome of manipulated HUVECs is significantly altered

**Fig. 5:**
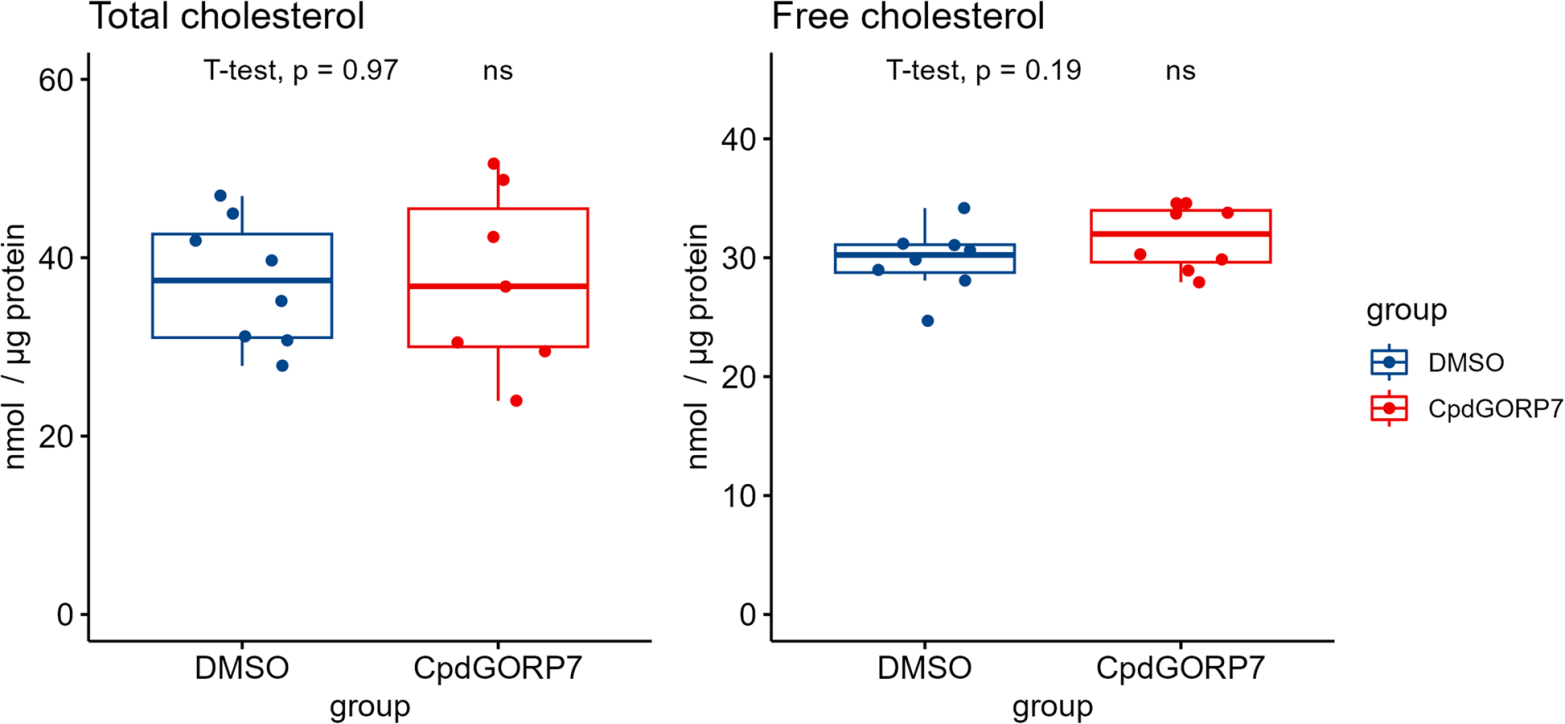
Total and free cholesterol levels in CpdG- and DMSO-treated cells. Box plots show on the *Y*-axis protein normalized cholesterol concentrations and on the *X*-axis each group measured. Blue represents DMSO control and red depicts CpdG-treated cells. Although no significant changes can be seen, a slight increasing tendency in mean free cholesterol levels is visible. *p*-values were determined using Student’s *T*-test, *N* = 8; values are from one experiment

As the OSBP family has been shown to transport multiple types of lipids [43], we decided to investigate how the manipulation of ORP7 affects the lipidomic profile of HUVECs and examined the cellular cholesterol levels using Amplex Red assay. We found no significant changes in the protein normalized concentrations of TC or free cholesterol (FC) in the CpdG-treated cells compared to the DMSO group, but an increasing tendency in the mean FC levels was seen (Fig. 5).

**Fig. 6:**
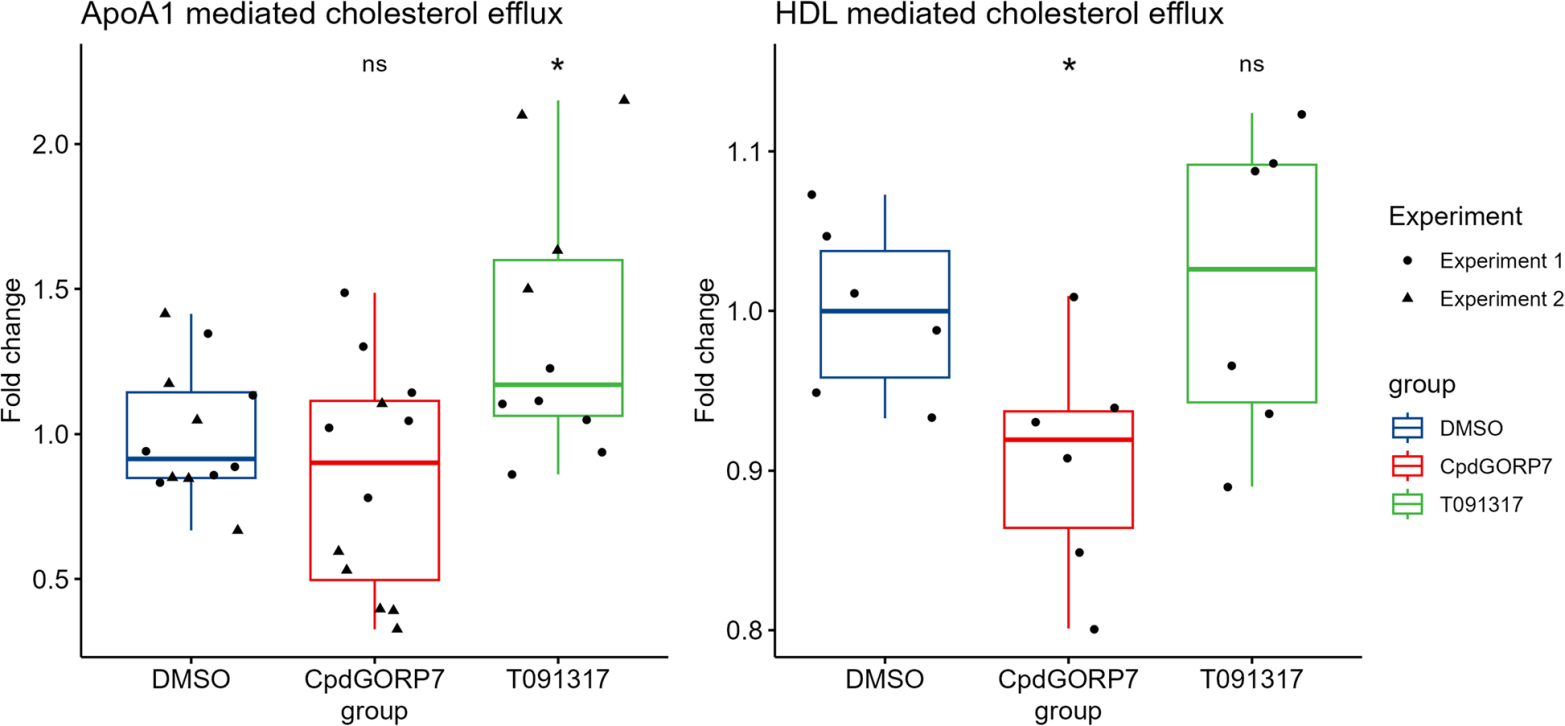
Cholesterol efflux measured as fold change in CpdG-, DMSO-, or T091317-treated HUVECs. Box plots are shown where *Y*-axis represents the fold change compared to the mean of DMSO control and the *X*-axis each group measured. Controls are shown in blue. The left facet shows a significant increase in cholesterol efflux to ApoA1 in T091317-treated cells, whereas the CpdG treatment did not show any significant increase, compared to DMSO control. On the right facet, a significant decrease in efflux to HDL in the CpdG-treated cells can be seen, whereas no notable change is visible in the T091317 or DMSO group. Pairwise *p*-values were determined using Student’s *T*-test against the DMSO group, and groupwise ANOVA was calculated using DMSO as reference; *p*-values for ANOVA statistics are 0.0043 in ApoA1-mediated cholesterol efflux and 0.055 in HDL cholesterol efflux. *N* = 12 for ApoA1, *N* = 6 for HDL per group. For ApoA1 experiments, values are from two independent experiments that are shown as dots or triangles; values for HDL are from one experiment

Although we did not see significant changes in the cholesterol pools of manipulated HUVECs, we wanted to study whether the already published increase in cholesterol efflux in CpdG-treated cells [10] is detectable in HUVECs. To this end, we performed a cholesterol efflux assay using ^3^H-labeled cholesterol and human ApoA1 or HDL as acceptors (Fig. 6). These experiments showed a significant increase in ApoA1 dependent cholesterol efflux with the positive control T091317, a known LXR agonist that induces ABCA1 mediated cholesterol efflux [44], but not with CpdG. However, a significant decrease in HDL-dependent cholesterol efflux was observed in CpdG-treated cells, which is mediated through ABCG1 [45] (Fig. 6).

Since we saw an evident drop in cholesterol efflux to HDL, we also analyzed membrane organization and cholesterol distribution in CpdG and DMSO treated HUVECs with a C-laurdan staining as well as with an mCherry-Domain 4 of Perfringolysin O (D4H) cholesterol probe.

**Fig. 7:**
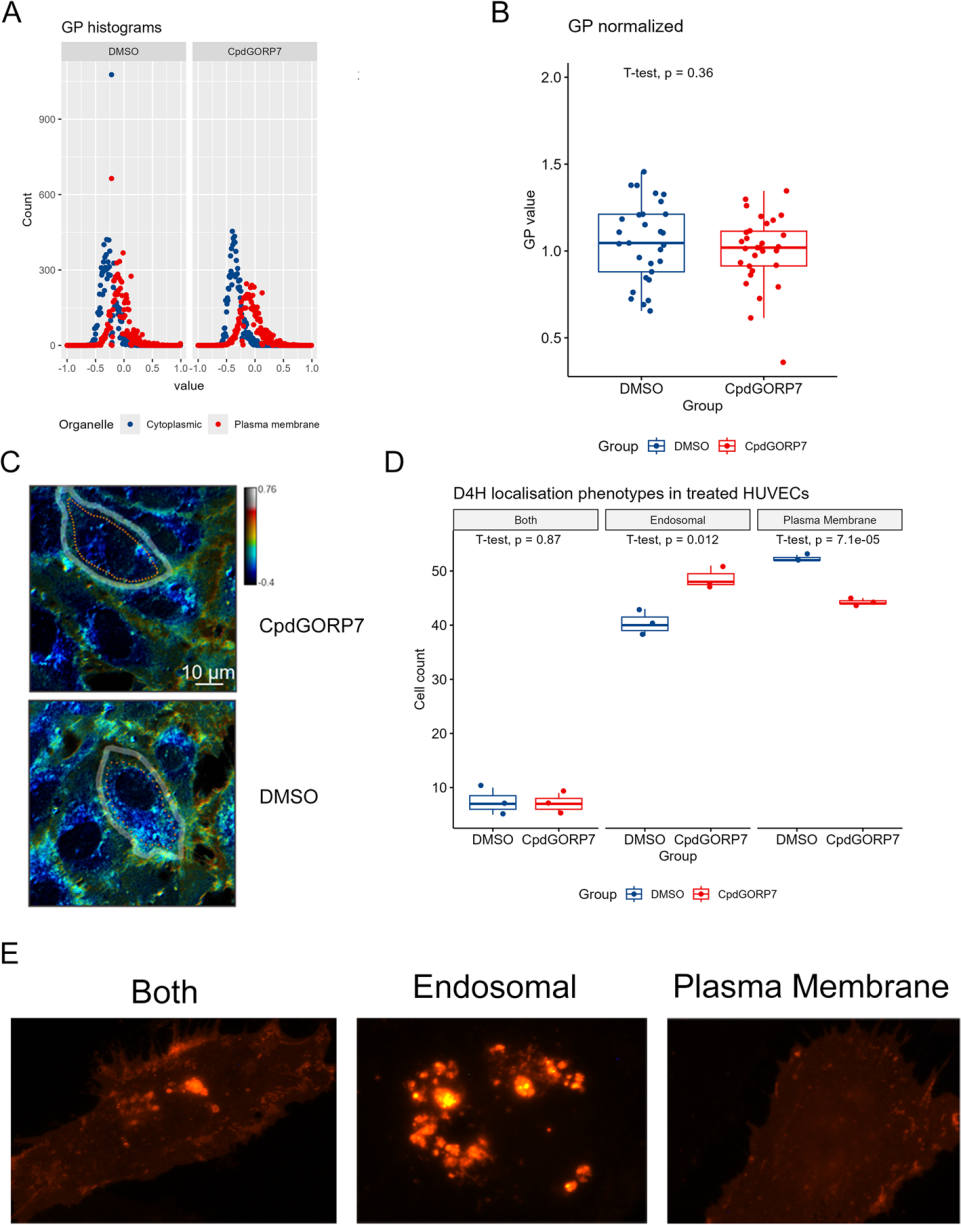
Cholesterol distribution in CpdG or DMSO-treated HUVECs. **A** Histogram of the GP values from the cells indicated by the dashed orange line in **D**. A ROI of the cytoplasmic (orange) and plasma membrane (green) area was used. The histogram shows how many pixels each GP value has. **B** Bar graph showing the normalized GP values of treated HUVECs stained with C-Laurdan. **C** Representative pseudocolored images of the samples (CpdG and DMSO). The dashed line shows the cell used for the GP histogram analysis in **B**. Images were from two replicates of each condition, and image counts were 30 images for CpdG and 29 images for DMSO treatment. **D** Cell counts of different cholesterol distribution phenotypes in CpdG- or DMSO-treated cells, representative images of phenotypes shown in **E**. Both represent a phenotype where PM was clearly visible with some endosomal D4H localization. Endosomal phenotype describes cells where no or very faint PM D4H localization could be seen, but the majority of D4H was clearly visible in endosomes. Cells with a plasma membrane phenotype show a clear PM and no or very little D4H localization to endosomal compartments. Cells were counted from three replicates and cell count was 100 cells per replicate. All values were from one experiment

**Fig. 8:**
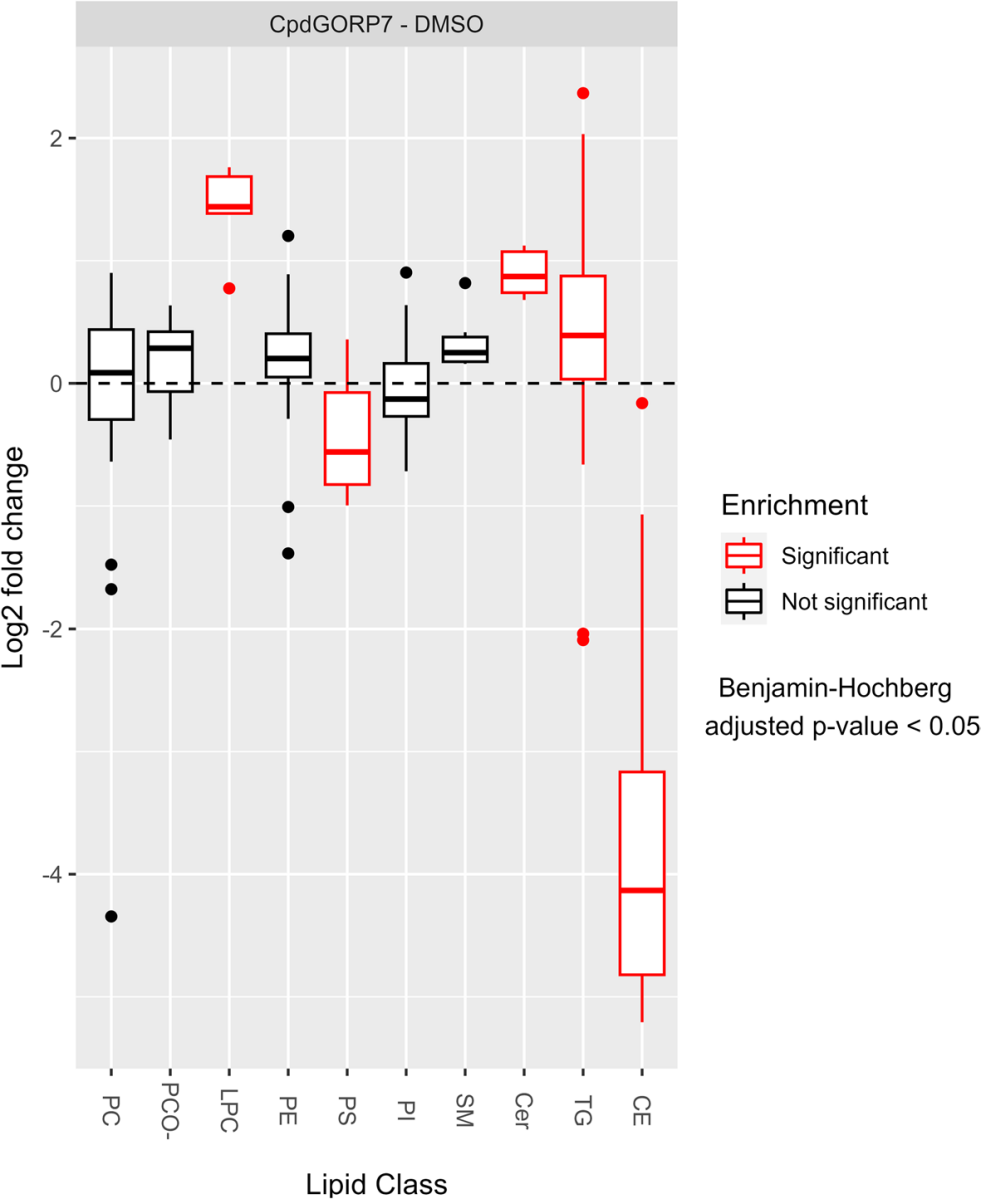
Log2 fold change distribution of different lipid classes in CpdG-treated HUVECs as compared to DMSO-treated controls. The *Y*-axis represents log2 fold change and *X*-axis each lipid class, significantly altered classes are shown in red, according to Benjamini–Hochberg adjusted *p*-values. The CpdG-treated HUVECs show significant changes in cholesteryl ester (CE), ceramide (Cer), lysophosphatidylcholine (LPC), phosphatidylserine (PS), and triacylglycerol (TG). Phosphatidylcholine diacyl and ether species (PC and PCO-, respectively), phosphatidylethanolamine (PE), phosphatidylinositol (PI), and sphingomyelin (SM) levels were the same. *N* = 4; values are from one experiment

CpdG-treated cells showed no significant difference in the distribution of ordered or disordered membrane regions in the C-laurdan analysis (Fig. 7A–C), whereas a small but significant change was seen in cholesterol distribution phenotypes by employing the mCherry-D4H probe (Fig. 7D–E). In CpdG-treated cells, more endosomal D4H distribution phenotypes were evident with a corresponding decrease in PM phenotypes, compared to the DMSO-treated control,

Next, we investigated how ORP7 inhibition affects the lipidome of HUVECs. We performed LC–MS/MS mass spectrometric analysis to get a systemwide view of the total lipidome changes in CpdG-treated cells (Fig. 8). When looking at lipid class level, the CpdG treatment induced a significant reduction of cholesteryl esters (CE) and phosphatidylserines (PS) and significant increases in ceramides (Cer), lysophosphatidylcholines (LPC), and TG (Fig. 8).

**Fig. 9:**
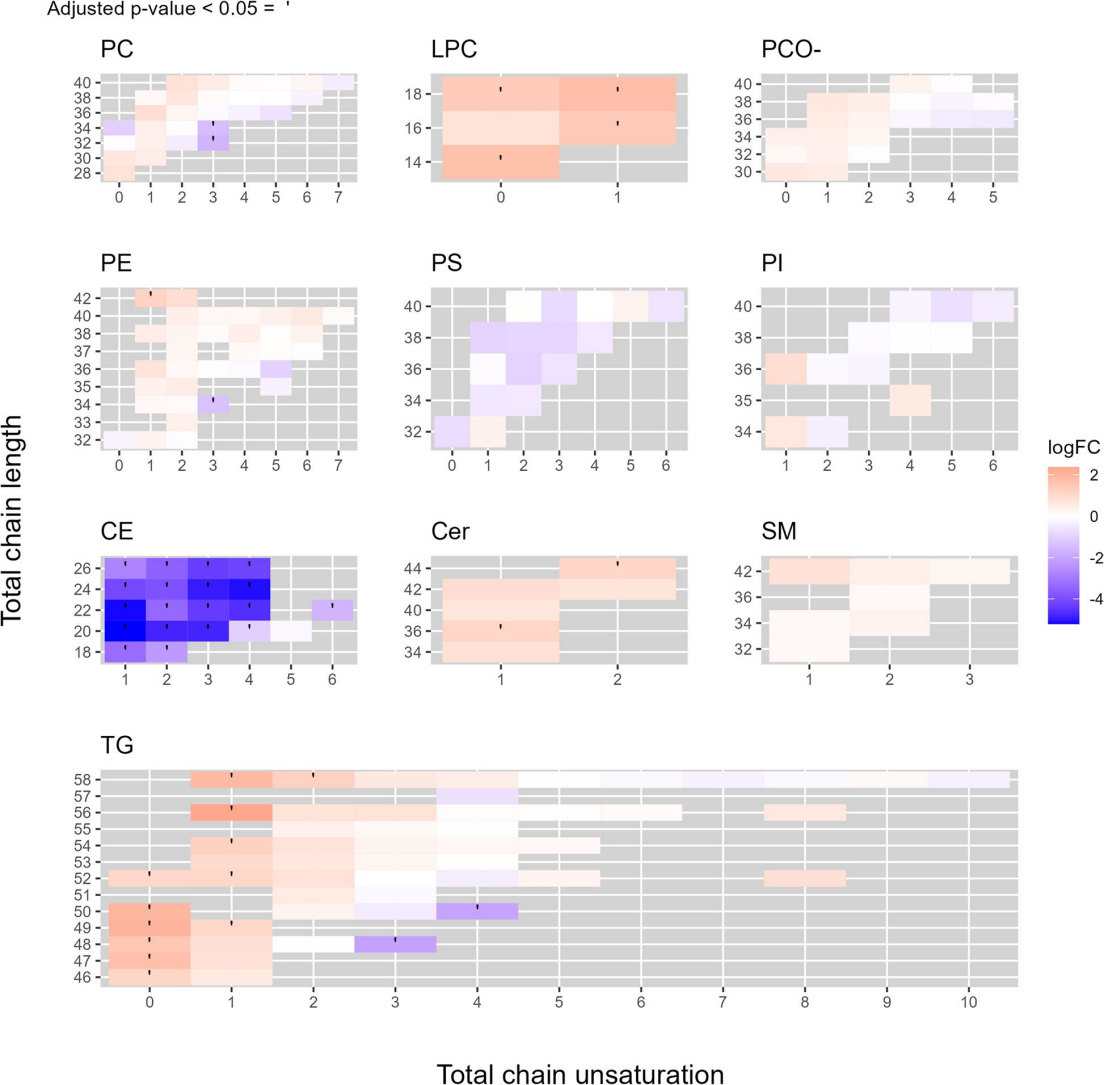
Lipid species-specific changes in CpdG-treated cells as compared to DMSO-treated controls. Each facet shows a different lipid class, where the *Y*-axis represents the total chain length, the *X*-axis total chain unsaturation, and each tile depicts a different lipid species. Each tile is colored according to the log2 fold change of each species, where orange represents an increase and blue a decrease; each statistically significantly altered species has been marked with a dot on the tile. Lipid class abbreviations are as in Fig. 8

Even though the levels of certain lipid classes had not changed significantly, there were differences in molecular species in each lipid class. Therefore, we plotted the log2 fold change of individual lipid species in each lipid class using a tile plot (Fig. 9).

ORP7 inhibition by CpdG reduced CEs and PSs, and increased LPCs and Cers across all molecular species, whereas other classes showed distinct lipid species-specific increases and decreases (Fig. 9). For example, saturated and monounsaturated TGs increased upon ORP7 inhibition, whereas a decrease was found in relatively short polyunsaturated species (TGs 48:3 and 50:4). The intermediates between those two types of TGs showed a slight increase or no change at all. Phosphatidylcholines (PCs), phosphatidylcholine-alkyls (PCO-), and phosphatidylethanolamines (PEs) exhibited a general pattern where ORP7 inhibition caused increases for most saturated and monounsaturated species (except PC 34:0). The clearest decreases due to ORP7 inhibition were found in certain relatively short polyunsaturated species (significant for PC 32:3, PC 34:3 and PE 34:3), whereas the longest highly unsaturated species showed mild, non-significant responses. The responses of sphingomyelin (SM), PS, and phosphatidylinositol (PI) species to ORP7 inhibition remained non-significant; all SM species displayed an increasing tendency, whereas a majority of phosphatidylserine (PS) and phosphatidylinositol (PI) species showed a decreasing tendency as compared to the control DMSO treatment.

**Fig. 10:**
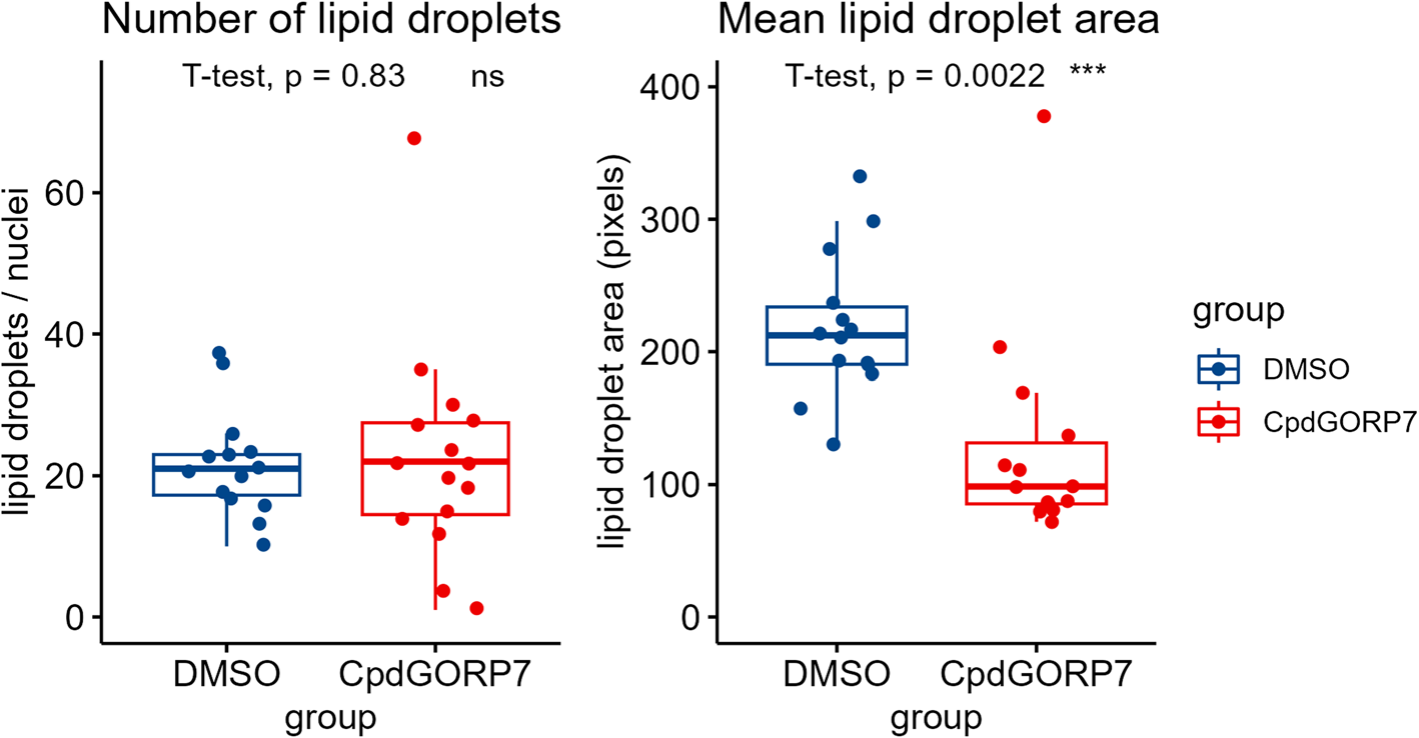
Lipid droplet metrics measured from CpdG-treated HUVECs using BODIPY 493/503 staining. The left panel displays mean number of lipid droplets per nuclei and right panel the mean lipid droplet area in pixels on the *Y*-axis; the *X*-axis shows each group measured. No notable change can be seen in the number of lipid droplets, but the mean area has decreased significantly in CpdG-treated cells. Pairwise *p*-values were determined using Student’s *T*-test against DMSO group, *N* = 13 images per group; values are from one experiment

As ORP7 inhibition by CpdG had decreased CEs drastically but simultaneously increased TGs, we investigated how the microscopic occurrence of lipid droplets in the HUVECs had changed, CEs and TGs being the main components of lipid droplets. BODIPY 493/503 staining was employed to visualize and quantify lipid droplets in the CpdG- and DMSO-treated cells. Even though the nuclei normalized lipid droplet number had not changed, we observed a drop in the mean area of lipid droplets in the CpdG-treated HUVECs (Fig. 10).

### Proximity biotinylation interactomics suggests interaction between ORP7, AKT1, and basement membrane components

To better understand the biological role of ORP7, we performed proximity biotinylation interactomics analysis by using an ORP7 with biotinylation tag. This resulted in a total of 1632 proteins being captured, most of which were also identified from control cells expressing the mycBirA bait. These data were then subjected to SAINT analysis, where the top 10 candidate ORP7 interaction partners were indicated (Table 2). Interestingly, these partners included multiple extracellular matrix (ECM) components: Fibronectin (FN1) Collagen Type IV Alpha 4 A 1 and 2 as well as 18A1 (COL4A1/2, COL18A1), along with Perlecan (HSPG2) and Multimerin 1 (MMRN1). Other proteins in the top 10 were RAC-alpha serine/threonine-protein kinase (AKT1) and Chaperonin Containing TCP1 subunit 7 (CCT7). Interactomics results for VAPA and VAPB, ER proteins acting as membrane anchors for several ORPs, did not produce any results in the SAINT analysis, but their unique spectrum counts were added in Table 2.

**Table 2:** SAINT interactomics results. As statistical metrics, the average probability (AvgP), maximum probability (MaxP), and false discovery rate (FDR) are shown for the top 10 proteins predicted to interact with ORP7 as well as the unique spectrum counts in each sample on the last 6 columns

Protein	AvgP	MaxP	FDR	ORP7_7	ORP7_6	ORP7_5	MYC_7	MYC_6	MYC_5
FN1	1	1	0	182	191	281	73	99	96
COL4A1	1	1	0	6	10	15	0	0	0
COL18A1	1	1	0	18	13	25	2	6	7
COL4A2	0.9642	1	0.009	3	9	9	1	0	2
HSPG2	0.9225	0.9975	0.0227	206	148	238	87	113	85
OSBPL7	0.9042	0.9925	0.0349	5	18	55	0	0	0
AKT1	0.885	0.9975	0.0463	1	14	17	2	2	4
CCT7	0.6667	1	0.0822	2	7	4	2	0	1
MMRN1	0.665	0.9975	0.1327	6	23	27	2	14	3
VAPA	NA	NA	NA	0	0	0	46	72	32
VAPB	NA	NA	NA	0	0	0	8	6	3

**Fig. 11:**
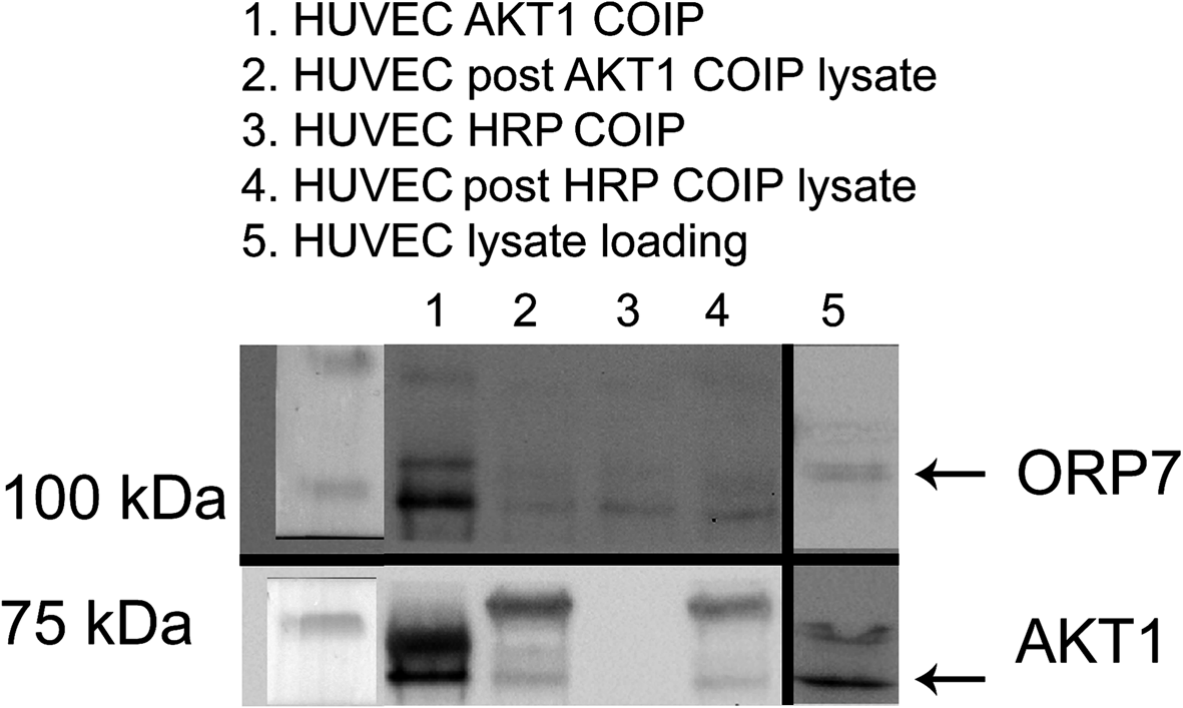
Western blotting results of co-immunoprecipitation using Protein-G-coupled magnetic beads incubated with AKT1 or mouse-HRP antibody used as a negative control. Samples are named at the top and corresponding lanes marked with numbers. Mobilities of molecular mass markers are indicated on the left, and each protein probed on the right with an arrow pointing to the wild type band. Samples from a representative experiment are shown

Given the overrepresentation of ECM components, and the fact that ORP7 has been shown to localize to the cytoplasmic face of the plasma membrane [6], we suspected that the interaction between ORP7 and these ECM components could be driven by AKT1, as it is unlikely that ORP7 would come into proximity with secreted or extracellular matrix proteins given its known localization in the cytoplasmic compartment. To confirm the suggested interaction of ORP7 with AKT1, we performed co-immunoprecipitation using ProteinG coupled magnetic beads and AKT1 antibody (Fig. 11). ORP7 and AKT1 were found co-precipitating from wild type HUVECs, while the proteins were absent in precipitates pulled down from negative control lysates incubated in the absence of the AKT1 antibody (Fig. 11).

As AKT1 has been shown to interact with and activate FAK at focal adhesions [46, 47], we also examined by western blotting if overexpression of ORP7 activates either of these kinases (Additional file 1: Fig. S11). The levels of phosphorylated FAK and AKT1 (pFAK, pAKT1) as well as those of unphosphorylated AKT1 appeared to be increased in the oexORP7 cells, but only the pFAK increase was statistically significant. No change in unphosphorylated FAK could be seen. In the CpdG-treated cells, the levels of both phosphorylated AKT1 and FAK, their unphosphorylated forms, or the pAKT1/AKT1 and pFAK/FAK ratios showed only small, statistically insignificant changes.

## Discussion

The present results show that inhibition of ORP7 with CpdG leads to a plethora of complex outcomes in endothelial cells, many of which likely represent secondary effects of the inhibition. These downstream effects ranged from the upregulation of lipid biosynthesis, oxidative stress, and inflammatory processes to reductions in cell division, proliferation, angiogenesis in vitro, and cholesterol efflux. We suspect that these outcomes are the result of dysfunctional lipid traffic from the ER to the plasma membrane, mitochondria, or autophagosomes, as will be discussed in the sections to come.

### Limitations

There are limitations related to the compound CpdG, which has also been shown to display a low affinity for cannabinoid receptor 1 (CNR1) [10]. Moreover, it is unknown whether CpdG could have off-target effects on other ORPs. In our transcriptomics dataset, the other ORP mRNAs showed upon CpdG treatment only minor log2 fold changes in the range of − 0.1–0.2. Concerning CNR, our transcriptomics analysis of CpdG- or DMSO-treated HUVECs detected no or just 1–2 transcripts for CNR1 or CNR2. Furthermore, CNR1 expression level is according to Human Protein Atlas data [48, 49] very low in endothelial cells (nTPM = 4) as for example inhibitory neurons (transcripts per million (nTPM) = 200) display almost 50 times more CNR1. It is also notable that immortalized HUVEC cell lines, such as HUVEC-TERT2, do not have any transcripts for CNR1, in line with our transcriptomics data. Given the abovementioned evidence, we studied by western blot analysis CNR1 levels in CpdG or DMSO treated HUVECs, Huh-7, and HeLa cells as well as in mouse hippocampal and spinal cord tissue lysates (as positive controls). These quantifications are displayed in Additional file 1: Fig. S12, where there are very faint or no visible bands for CNR1 in the HUVEC, Huh-7, and HeLa lysates (Additional file 1: Fig. S13 lanes 1–6), whereas strong bands are seen in mouse nervous tissue lysates (Additional file 1: Fig. S13 lanes 7–8). The size difference between human cell and mouse tissue ORP7 is likely due to splice variant differences [50, 51] or post-translational modifications.

Given the evidence that HUVECs express CNR1 neither at mRNA nor at protein level, it is most likely that the effects of CpdG in HUVECs are driven by ORP7 inhibition.

### System-wide omics results point toward changes in inflammatory processes, lipid metabolism, and oxidative stress

Gene set analysis of our transcriptomic data showed decreases in mRNAs related to cell cycle, cell division, and nucleotide synthesis as well as increases in genes related to inflammation, oxidative stress, and lipid metabolism. Lipidomic analysis, on the other hand, exhibited increases in lipid classes known to increase inflammation and endothelial cell dysfunction, such as Cers [52, 53], TGs [54, 55], and LPCs [56].

#### Inflammation

The relative upregulation of inflammatory processes in the transcriptomics data can most likely be attributed to genes encoding pro-inflammatory proteins such as VCAM1, ICAM1, and SELE, which are shown to be elevated in the initial stages of atherosclerosis [24]. A vital anti-inflammatory molecule also affected is the transcription factor *KLF4* that controls the expression of multiple inflammation-associated genes [57], of which *CH25H* is a pivotal example in our transcriptomics dataset [58]. Another anti-inflammatory gene upregulated upon CpdG treatment is *STC1*, which mediates inflammation by inhibiting the tissue infiltration of leukocytes [33]. Moreover, downregulation can be observed in certain pro-inflammatory genes such as *THY1*, *IL1RL1*, and *IL7R* [37, 38]. Other evidence suggesting that CpdG-treated HUVECs show inflammatory activation is the induction of genes of arachidonic acid (AA)-derived lipid mediator synthesis i.e., *PTGS2*, *SLOC2A1*, and *CYP1A1.* Lipid mediators are auto- and paracrine signals produced from membrane phospholipids [17–22], especially from the hydrolysis of PC that produces LPC species, which had decreased and increased, respectively, in our lipidomic data. *PTGS2* converts phospholipid-derived AA to pro-inflammatory prostanoids, and *CYP1A1* may convert AA to epoxyeicosatrienoic acids and hydroxyeicosatetraenoic acids, having both anti- and proinflammatory properties. It is possible that the CpdG-treated cells are trying to produce more epoxyeicosatrienoic and hydroxyeicosatetraenoic acids by upregulating *CYP1A1* [59, 60], as its log2 fold change is almost three times that of *PTGS1/2*. Since we did not see significant changes in lipoxygenase genes (*ALOX*), we suspect that neither leukotriene nor lipoxin synthesis had changed [61]. The observed increase in Cers and LPCs, and upregulation of biosynthetic genes of AA-derived lipid mediators together suggest that inflammatory signaling is activated in the CpdG-treated cells. Moreover, we saw a substantial increase in *CH25H* expression (converts cholesterol to 25-hydroxycholesterol), which suggests that cholesterol might be increasingly turned into oxysterols [62]. This notion is supported by the increase in the expression of *CYP27A1* catalyzing the synthesis of 27-hydroxycholesterol [63]. We did not see significant changes in the cholesterol biosynthetic pathway genes but did observe an increase in liver X receptor (LXR) expression, which could represent a compensatory response to enhance cholesterol efflux, as we saw a significant decrease in ABCG1 and HDL-mediated cholesterol efflux.

Since cholesterol biosynthesis genes had not changed, it is likely that the CpdG-treated cells could have turned excess FC into oxysterols [64], which could mediate a pro-inflammatory signal.

#### Accumulation of efflux accessible cholesterol remains elusive

Why CpdG-treated cells are consuming CEs from lipid droplets, as suggested by our indirect measurements of reduced mean area of lipid droplets and drastic reductions in all CE species, is thus far unknown and contrary to data on kidney glomerular podocytes where ORP7 silencing was shown to increase lipid droplet counts [16]. CEs could be used to balance the plasma membrane level of chemically available cholesterol, which might be reduced given the fact that ABCG1-mediated cholesterol efflux was reduced, and no change in ABCA1-mediated cholesterol efflux was evident. As there was a slight increase in endosomal cholesterol distribution in the CpdG-treated cells, we speculate that the reduction of cholesterol efflux to HDL could be due to the accumulation of FC in endosomes or lysosomes where it is inaccessible for CE synthesis or efflux. We also observed an increase in the expression of *NPC1* and *NPC2*, which traffic FC to the lysosomal limiting membrane, after which it is further transported to the PM [65]. This could be related to the accumulation of cholesterol in the endosomal compartments and represent a compensatory mechanism with the purpose of removing excess cholesterol from endo-lysosomes. Although we could not by C-laurdan detect alterations of the PM domain organization in the CpdG-treated cells, the above evidence is in line with previously reported increases in membrane fluidity in ORP7 silenced podocytes [16], as reduced FC at the PM has been associated with increased membrane fluidity [66–68].

Decrease in ABCG1 mediated cholesterol efflux seems peculiar considering that the reduction in CEs should make more FC available for efflux. The increase in Cers and reduction of cholesterol efflux but no difference in PM order in C-laurdan visualization could suggest that FC is in CpdG-treated cells accumulating in non-PM raft domains [69], where it is unavailable for both efflux and other cellular functions. The increase in LPC could also be the result of Cer and FC accumulation in raft domains, as LPC can make the membrane environment more hospitable for rigid raft domains [70].

#### Oxidative stress response and possible association of ORP7 with mitochondria-associated membrane (MAM)

The observed reductions in mRNAs related to cell cycle, cell proliferation, and nucleotide synthesis are puzzling. We suspect that this reduction could be due to mitochondrial dysfunction or cellular stress responses. Given that transcriptomics data showed increased NES in lipid metabolism and oxidation related gene sets, such as the oxidative stress response, the occurrence of increased oxidative stress upon CpdG treatment is likely. Oxidative stress can result in the release of cytochrome C from mitochondrial cardiolipin [71], which in turn downregulates cellular division and proliferation through caspase activation [72]. Some evidence pointing toward this conclusion is present in our transcriptomics dataset as, for example, the NF-kB/AP-1 induced apoptosis pathway showed a positive NES. Further evidence from other cell models have shown that ORP7 silencing can initiate ER stress and induce apoptosis [16]. GABARAPL2 and LC3B have been shown to be necessary for mito- and autophagy [73–75] and have high affinities for membranes with cardiolipin and Cers [76], such as those seen at both mitochondrial outer and inner membranes or MAM [77, 78]. MAM lipid raft-like domains are also necessary for autophagy [79], which suggests that GABARAPL2 and LC3B could be associated with these raft-like domains as MAM is enriched with cardiolipin [77]. It has also been shown that Cers are necessary for mitophagy, especially the C-18 Cer (Cer 18:1;O2/18:0, Cer 36:1 in Fig. 9) [80, 81]. We would like to speculate that Cers and FC may upon CpdG treatment accumulate in MAM lipid raft like domains, since Cers and cholesterol are known to be enriched at both the PM and MAM, and MAM raft-like domains are needed for autophagy [77, 79]. Therefore, an increase of Cers and FC at MAM and a subsequent increase in MAM raft-like domains could be a marker for increased autophagic potential. Given that ORP7 has been shown to interact with both GABARAPL2 and LC3B independently, and both proteins interact with each other, we suspect that ORP7 may play a role in MCSs between MAM, mitochondria or autophagosomes. Our transcriptomic data showed inconsistent patterns in the autophagy related genes (Table 1), but we did see an increase in LC3A/B (MAP1LC3A/B) and GABARAPL1, but not in GABARAPL2. Which lipids ORP7 might transfer at these MCSs still remains elusive but reciprocal transport of cholesterol and/or phosphoinositides would be a likely option as several ORP proteins have been shown to transport these lipids [4, 82, 83].

### Interaction of ORP7 with AKT1

As was evident from our interactomics data, we were unable to confirm the ORP7-VAPA or -VAPB interaction. This is puzzling as ORP7 does contain a FFAT motif, and the interaction between VAPA and ORP7 has been confirmed with GST-pull down [84], BIFC [85], and high-throughput methods [86]. We were also unable to confirm the interaction between ORP7 and GABARAPL2 or LC3B through proximity biotinylation. The reasons for the lack of these interaction partners in our dataset could be multiplex. It is unlikely that the reason is low protein concentrations in our pull-down samples, as endothelial cells have 6 to 7 times higher expression of GAPARAPL2 (231 nTPM), VAPA (197.5 nTPM), and LC3B (176 nTMP) as compared to AKT1 (31.6 nTPM), according to Human Protein Atlas [48]. Other explanations for the lack of these interactions in our dataset could be that the mycBirA epitope of the ORP7 construct does not come to sufficient proximity of these proteins. As we alluded to in the results section, we suspect that the interaction between the ECM components and ORP7 could be driven through their interaction between AKT1, as AKT1 has been shown to interact with and regulate focal adhesions [47]. Moreover, the overexpression of ORP7 did moderately increase the phosphorylation of FAK, although the underlying mechanism remains unknown. Of note, ORP7 would not be the only ORP that interacts with AKT1 as ORP2 and ORP9 are also shown to bind to AKT1 [87–89].

### Reduced angiogenic capacity as a result of systemic changes

The present results revealed an angiogenic defect in the CpdG-treated cells. Our transcriptomic dataset included little evidence pointing towards a compensation for the reduced angiogenic potential, as only VCAM1, SELE, and ICAM1 were upregulated, which have been shown to promote angiogenesis [23, 90]. Although not shown in human endothelial cells, increase in PTGS2 activity has been shown to upregulate the expression of VEGF and therefore increase angiogenesis in tumors [91, 92]. Our data revealed a slight increase in both *VEGFA* and *VEGFB* and a slight decrease in *FLT1*, which could point toward a similar mechanism in HUVECs. Even though our C-laurdan analysis failed to yield evidence for a change in PM lipid organization, probing of the cholesterol distribution with mCherry-D4H indicated that cholesterol could be redistributed towards endo-lysosomes upon CpdG treatment. The observed angiogenic defect may thus be the result of an unfavorable lipid composition, such as the reduction in FC available at the PM, which has been shown to reduce angiogenesis [93]. It is well established that PM cholesterol and sphingolipid rich lipid rafts are vital for normal angiogenesis [94, 95]. Other reasons for the reduction in angiogenic capacity could be the downregulation of cell cycle and cell division genes by cytochrome c release and activation of caspases, which could hamper angiogenic tube formation in vitro.

## Conclusions

This study shows that ORP7 inhibition in primary endothelial cells leads to an increase in inflammatory gene expression, apparently due to an elevation of pro-inflammatory lipid classes such as Cers and LPCs, as exemplified by the upregulation of pro-inflammatory genes such as *VCAM1*, *ICAM1*, and *SELE*. Our data also revealed that endothelial cells upregulate genes responsible for the synthesis of oxysterols, epoxyeicosatrienoic, and hydroxyeicosatetraenoic acids, as well as prostanoids, putatively to regulate inflammation. Inhibition of ORP7 also leads to a decrease in both ABCG1-mediated cholesterol efflux and angiogenesis but has no effect on ABCA1 mediated cholesterol efflux. Furthermore, we were also able to prove an interaction between ORP7 and AKT1 through two independent methods. This study represents the first of its kind to characterize the results of ORP7 inhibition and overexpression in a comprehensive manner in any cell type. We believe this study will serve as the basis for a larger body of work on ORP7 functions in endothelial cells as well as in other cell and animal models. More work is needed to study whether and how ORP7 inhibition affects the levels of oxysterols and prostanoids along with inflammation and whether ORP7 also modulates the formation of raft-like membrane domains. Moreover, it remains to be studied whether and how ORP7 manipulations may induce mitochondrial dysfunction. The lipid transport functions of ORP7 also remain unknown, but our work suggests that ORP7 could play a role in autophagosome formation at MAMs, for example by trafficking cholesterol between ER, mitochondria, and autophagosomes.

## Methods

**Fig. 12:**
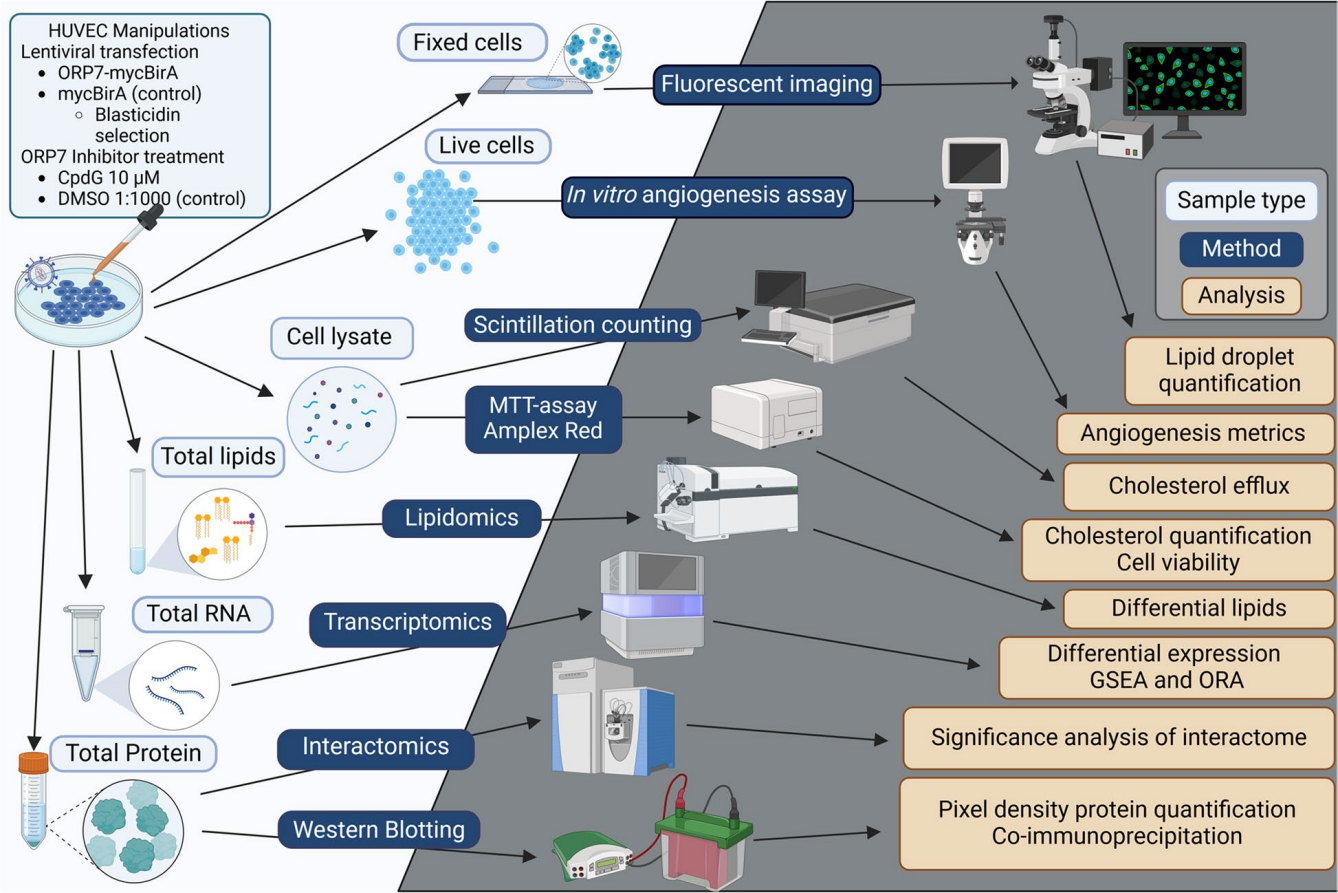
Simplified illustration of the experimental workflow used in this study. Light-blue text boxes depict sample types, whereas dark blue text boxes show the methods used, and light-yellow boxes define the feature or function analyzed (created with BioRender.com)

The following section will outline the methods and reagents used during this study; most of the workflows have been condensed and illustrated in Fig. 12. The aim was to study the macromolecule profile of pharmacologically ORP7-inhibited HUVECs, to gain a better understanding on the role of ORP7 and MCS in endothelial cells.

### cDNA constructs and mRNA quantification by quantitative real-time PCR (qPCR)

cDNA constructs were made in brief as follows: inserts were produced by PCR using Dynazyme II according to manufacturer’s instructions, using primers 1 and 2 in Table 3.

**Table 3:** Cloning and qPCR primers used in this study

#	Sequence	Restriction site or gene	Direction	Use
1	TTTTAGATCTATGGACTTCCAAGAGAGGGA	Bgl II (OSBPL7)	Forward	Cloning
2	TTTTGCGGCCGCGCCCAACTTTGTACAAGAAAGC	Not I (OSBPL7)	Reverse	Cloning
3	TTTTGTCGACATGGACTTCCAAGA	Sal I (OSBPL7)	Forward	Cloning
4	TTTTGCGGCCGCTACTTCTCTG	Not I (OSBPL7)	Reverse	Cloning
5	ACCCACCCTATGAACAACATGA	ABCA1	Forward	qPCR
6	GAGTCGGGTAACGGAAACAGG	ABCA1	Reverse	qPCR
7	CGGCCTACTCCTCCACATACC	OSBPL7	Forward	qPCR
8	ACTGTTCCCACAGGCACAATC	OSBPL7	Reverse	qPCR
9	TGGTCATCCAGCAGGTGTTCGA	RPLP	Forward	qPCR
10	ACAGACACTGGCAACATTGCGG	RPLP	Reverse	qPCR

An ORP7 cDNA construct in pEGFP-C1 vector and pMycBirA (kind gift from Johan Peränen, University of Helsinki) were digested using Bgl II and Not I and the ORP7 ORF was ligated into pMycBirA. The obtained plasmids were validated by Sanger sequencing at the Institute for Molecular Medicine Finland FIMM Genomics unit supported by HiLIFE and Biocenter Finland. Sequence reads were mapped to a reference sequence of each plasmid generated with Benchling using UniPro UGENE software [96]. Correct constructs where then moved to pENTR2B with Sal I and Not I/Xho I, in a similar manner using primers 3 and 4 in Table 3. The constructs were transferred to pLenti6.3/V5-DEST by Gateway (Thermo Fisher Scientific, Waltham, MA) recombination at the Helsinki University Genome Biology Core (HiLIFE Helsinki and Biocenter Finland).

qPCR validation of select RNAseq observations was carried out on Roche Lightcycler™ 480 II instrument by using Sybr Green chemistry, Roche qPCR Master mix, and primers specified in Table 3. *RPLP* was used as housekeeping control mRNA. Fold changes in gene expression were calculated by employing the -DDC_T_ method.

### Cell culture, inhibitor treatment, and lentiviral transfection

HUVECs were grown in EGCM2 media (PromoCell cat #: C-22111) without antibiotics, supplemented with endothelial growth mix 2 (PromoCell cat #: C-39216) to passage 6. HUVECs at passage 2 were transduced on 6-well plates using lentiviral particles packaged by the Helsinki Biomedicum Virus Core supported by HiLIFE and the Faculty of Medicine, University of Helsinki, and Biocenter Finland. Cells were incubated with the lentiviruses encoding ORP7-mycBirA or oexControl for 48 h with an approximate MOI (multiplicity of infectivity) of 15. After lentiviral transduction, the cells were selected using media with (1:4000 of 10 mg/mL Blastidicin, Gibco) for 5 days. Transduced cells were then expanded from 6-well plates to 10 cm plates without antibiotics to passage 6 and used for experiments. For ORP7 inhibition, cells were treated with 10 μM concentration of CpdG (Medchemexpress, cat #: HY-143200, OSBPL7-IN-1) or with 1:1000 dilution of DMSO unless otherwise specified.

### MTT assays

Metabolic activity of CpdG or DMSO treated and oexORP7 or oexControl cells was measured using CellTiter 96® AQueous One Solution Cell Proliferation Assay (Promega cat #: G358C), according to the manufacturer’s instructions. Absorbance at 490 nm was measured using a PerkinElmer EnSpire Multimode Plate Reader. Percentage of metabolic activity was calculated by the following formula: $100+( \frac{control-sample}{control}*100)$, where control is the absorbance of normal HUVECs or oexControl and samples are oexORP7 cells or cells treated with CpdG at 1,5,10,25,50,75,100, 250, and 500 μM concentrations for 24 h.

### Western blotting

OexORP7 and oexControl as well as CpdG- or DMSO-treated cells (24 h) were washed twice with ice cold PBS and then lysed in 200 μL lysis buffer [150 mM NaCl, 0.5 mM MgCl_2_, 10% Glycerol,0.5% Triton X-100, 0.5% sodium deoxycholate (v/v)] with 1X EDTA-free Protease Inhibitor Cocktail (Merck cat #: 04693159001) and with or without 1X phosphatase inhibitor (Roche, cat #: REF 04 906 845 001, lot #: 37,590,700) for 20 min at + 4 °C. Lysates were collected to a fresh tube and spun for 15 min at 13,000 × g at + 4 °C, after which supernatant was moved to a fresh tube. Sample protein concentrations were measured with Thermo Fisher Pierce™ BCA Protein Assay Kit (cat #: 23,225), and the samples were blotted with a BioRad Trans-Blot Turbo Transfer System. Membranes were blocked for 1 h at room temperature (RT) in 5% bovine serum albumin (BSA) for phosphospecific antibodies or for 15 min in BioRad EveryBlot solution and then probed overnight at + 4 °C with antibodies shown in Table 4. Membranes were washed thrice for 10 min with 1 X TBST at RT and then incubated with 1:2000 dilutions of HRP-conjugated secondary antibodies in Milk-TBST or EveryBlot for 1 h at RT. Membranes were washed as above and detected using Thermo Fisher Pierce ECL Western blotting substrate (cat #: 32,106, lot #: WJ335099) according to the manufacturer’s instructions. We used BioRad Precision Plus Protein All Blue Prestained as a protein standard for all membranes (BioRad, cat#: 1,610,373).

**Table 4:** Antibodies used in this study

Protein	Dilution/μg	Manufacturer	Catalog number	Lot number	Method
ORP7	1:1000	Sigma Aldrich	HPA036076	NA	WB
pFAK	1:500	Invitrogen	44-626G	NA	WB
FAK	1:1000	Invitrogen	AHO0502	NA	WB
pAKT1	1:1000	Cell Signalling	4051S	NA	WB
AKT1	1:1000	Invitrogen	AH01112	YG373683	WB
AKT1	10	Proteintech	10,175–2-AP	00127621	COIP
ACTB	1:5000	Sigma Aldrich	A2066-2ml	106m4770V	WB
cMYC	1:1000	Santa Cruz Biotechnology	sc-40	NA	WB
CNR1	1:500	Abnova	H00001268-M01	ZG4389981A	WB
HSPG2	10	Invitrogen	PA5-119,185	ZG438884	COIP
Mouse-HRP	1:2000/10	Jackson ImmunoResearch	115–035–044	138,415	WB/COIP
Rabbit-HRP	1:2000/10	Jackson ImmunoResearch	111–035-003	156,592	WB/COIP

### Co-immunoprecipitation

Normal HUVECs were grown on 10 cm plates until 80% confluent, after which they were washed twice with 1X cold PBS on ice. Cells were lysed to 1 mL of the lysis buffer described in the “Western blotting” section that included both protease and phosphatase inhibitors and incubated for 30 min at + 4 °C. Lysates were spun for 15 at 13,000 × g at + 4 °C, and supernatant was collected to a fresh tube. Magnetic beads (Thermo Fisher, Pierce Protein G Magnetic Beads, cat #: 88,847, lots #:YF379145, ZC387761) were washed twice with 1 X TBST, after which 25 μL was added to 500 μL of cell lysates and mixed on a roller for 1 h at + 4 °C. Either 5 μL of Mouse HRP antibody or 20 μL of AKT1 antibody (see the “Western blotting” section) was added to 25 μL of washed beads and mixed on a roller for 2 h at + 4 °C. Beads were removed from each mix by incubating on a magnetic rack for 2 min at + 4 °C, after which antibody coupled beads were added to the pre-cleared lysates and incubated o/n at + 4 °C. Lysates were removed to a fresh tube by incubating on a magnetic rack and beads were washed thrice for 5 min with lysis buffer at + 4 °C. To the washed beads, 40 μL of 2 X Laemmli buffer (BioRad) was added and incubated for 10 min at + 50 °C. Magnetic beads were removed as described before and to each elution 1 mL of 2-β-mercaptoethanol was added, and samples were boiled for 5 min at + 100 °C. Samples were spun down and Western blotting was performed as described in the “Western blotting” section.

### Angiogenesis assay

Angiogenesis assay (Millipore cat #: ECM625, lot #: 3,183,813) was performed on oex cells (oexControl and oexORP7) or cells treated for 24 h with CpdG or DMSO at 10 μM or 1:1000 concentrations, respectively. The oex cells were incubated in normal EGCM2 media. Cells were imaged by using a bright-field set-up of EVOS M5000 (Invitrogen) microscope after 8 h. Cell junctions were counted from binary images using Fiji [97] Angiogenesis analyzer plugin [42].

### Measurement of cholesterol concentration

Amplex Red™ cholesterol assay (Invitrogen, cat #: A12216, lot #: 2,422,702) was performed according to manufacturer’s instructions from undiluted samples of oex cell (oexControl and oexORP7) lysates or 1:4 dilutions of CpdG or DMSO treated cell lysates, made as in the “Western blotting” section. Assays were performed for TC with cholesterol esterase treatment or free cholesterol (FC) without esterase treatment, and the cholesterol concentrations were normalized to protein concentrations measured as in the “Western blotting” section.

### Cholesterol efflux to ApoA1 and HDL

Normal HUVECs or oexORP7 and oexControl cells were plated at 50,000 cells per 12-well plate and left to adhere for 24 h, after which the cells were washed twice with 1 PBS and then 1 mL of media with 0.2 μCi/mL of ^3^H-cholesterol and 5 mg/mL Sandoz 58–035 SOAT inhibitor was added. After a 24-h incubation, media were removed, and cells were washed thrice with 1 X PBS, after which 2 mL of FBS-free EGCM (Sigma Aldrich, cat #: 211F-500) media with 1 mg/mL of BSA was added. To the HUVEC media, either 10 μM of T0901317, 10 μM CpdG, or 1:1000 (v/v) DMSO was added and incubated for 24 h. After incubation, a 1-mL aliquot was taken from each well and spun at 800 × g for 5 min, and the supernatant was collected to a scintillation tube. For ApoA1-mediated efflux, 100 μL of FBS-free media with 50 μg/mL ApoA1 was added, and for HDL-mediated efflux, 111 μL of 450 μg/mL HDL isolated from human plasma and dialyzed to PBS was added and incubated for 24 h. Finally, media was collected and spun as before, and supernatant was collected to scintillation tubes. Cells were lysed to 300 μL of 1% SDS and collected to scintillation tubes. To each scintillation tube, 3 mL of scintillation cocktail (Ultima Gold, cat #: 6,013,326, lot #: 23,151, Perkin Elmer) was added, and tubes were mixed briefly before they were subject to liquid scintillation counting with a Wallac Winspectral 1414 counter (Perkin-Elmer, Waltham, MA).

### Immunofluorescence staining, fluorescence microscopy and lipid droplet quantification

HUVECs were seeded at 10,000 cells/coverslip and left to adhere for 24 h in 50 μL of EGCM2. OexORP7 and oexControl cells and normal HUVECs were left to adhere for 24 h, after which the oex cells were fixed and normal HUVECs were incubated for another 24 h in 100 μL of media containing 10 μM of ORP7 inhibitor or 1:1000 DMSO. Cells were fixed with 4% PFA in PBS and blocked in 1% BSA in PBS. Lipid droplet staining was performed by incubating fixed cells for 1 h at RT with 1:1000 of BODIPY493/503 stock (50 mM) and mounted with Mowiol:Dabco:DAPI solution. Imaging of the specimens was carried out on a Zeiss Observer.Z1 fluorescence microscope (Serial #: 3,834,002,509, Zeiss Group), with a 63 X immersion oil objective. Lipid droplets were quantified as specified in [98].

### Lipidomics

Lipids from oex and CpdG or DMSO treated cells were extracted according to Folch et al. [99]. Solvents were evaporated and the lipid extracts immediately dissolved in chloroform/methanol 1:2 (by volume). Internal standard mixture (SPLASH® LIPIDOMIX® and Cer 18:1;O2/17:0, both from Avanti Polar Lipids, Alabaster, AL, USA) was added to the samples, which were analyzed with LC–MS/MS as previously described [100]. In brief, samples were analyzed with acetonitrile/water/isopropanol-based solvent system [101] by employing Agilent 1290 Infinity HPLC (Agilent Technologies, Santa Clara, CA) equipped with a Luna Omega C18 100 Å (50 × 2.1 mm, 1.6 μm) column (Phenomenex) and Agilent 6490 Triple Quad LC/MS with iFunnel Technology. The lipid species were identified and quantified using lipid class-specific detection modes, as previously described [100]. Retrieved spectra were processed by Mass Hunter Workstation qualitative analysis software (Agilent Technologies, Inc.), and individual lipid species were quantified using the internal standards and LIMSA software [102].

### Transcriptomics

RNA was extracted from oexORP7 and oexControl or, CpdG- and DMSO-treated cells with the RNAeasy Mini kit (Qiagen cat #: 74,104) according to the manufacturer’s instructions, with the following modifications: samples were homogenized by passing lysate through a 200-μL pipette tip 10 times, on-column DNAse 1 digestion was performed according to manufacturer’s instructions, and samples were eluted to 30 μL of RNAse-free H_2_O. RNA integrity, library preparation, and RNA sequencing were performed according to the following instructions by GENEWIZ Germany GmbH (Leipzig, Germany). RNA samples were quantified using Qubit 4.0 Fluorometer (Life Technologies, Carlsbad, CA, USA), and RNA integrity was checked with RNA Kit on Agilent 5300 Fragment Analyzer (Agilent Technologies, Palo Alto, CA, USA). RNA sequencing libraries were prepared using the NEB Next Ultra RNA Library Prep Kit for Illumina following manufacturer’s instructions (NEB, Ipswich, MA, USA). Briefly, mRNAs were first enriched with Oligo(dT) beads. Enriched mRNAs were fragmented for 15 min at 94 °C. First-strand and second-strand cDNAs were subsequently synthesized. cDNA fragments were end repaired and adenylated at 3′ ends, and universal adapters were ligated to cDNA fragments, followed by index addition and library enrichment by limited-cycle PCR. Sequencing libraries were validated using NGS Kit on the Agilent 5300 Fragment Analyzer (Agilent Technologies, Palo Alto, CA, USA) and quantified by using Qubit 4.0 Fluorometer (Invitrogen, Carlsbad, CA). The sequencing libraries were multiplexed and loaded on the flow cell on the Illumina NovaSeq 6000 instrument according to manufacturer’s instructions. The samples were sequenced using a 2 × 150 Pair-End (PE) configuration v1.5. Image analysis and base calling were conducted by the NovaSeq Control Software v1.7 on the NovaSeq instrument. Raw sequence data (.bcl files) generated from Illumina NovaSeq was converted into fastq files and de-multiplexed using Illumina bcl2fastq program version 2.20. One mismatch was allowed for index sequence identification.

### Biotin proximity labeling interactomics

Interactomics was performed on HUVECs transduced with oexORP7 or control oexControl vectors detailed in the “cDNA constructs and mRNA quantification by quantitative real-time PCR (qPCR)” section. Transduced cells on 6-well plates were selected for approximately 5 days with 10 μg/mL of blasticidin until all untransduced HUVECs were dead, and then left to grow until confluent, after which cells were expanded to passage 6. Samples were created as described in [103] section “Procedure” steps 47–60, from three 10-cm plates of transduced and selected cells for each sample. LC–MS/MS was performed by University of Helsinki Viikki Proteomics Unit (Helsinki, Finland) as described in [103].

### Bioinformatics and statistics

RNA-seq analysis was performed according to the following pipeline using the Chipster suite [104]: pair ended reads were clipped with Triommatic (phred ≥ 30) [105] and then aligned to the human genome (GRCh38) with STAR [106], aligned reads were counted using HTSeq [107], and differential expression-analysis was performed using DESeq2 [108], and log2 fold change shrinkage was estimated using apeglm [109]. Overrepresentation analysis (ORA) was performed using all genes that had an adjusted *p*-value of less than 0.05, gene set enrichment analysis (GSEA) was performed using the whole RNAseq dataset and ranked according to log2 fold change, and ties were resolved at random. Both ORA and GSEA were performed using the R package ClusterProfiler [110]. Lipidomic data analysis was performed using the Bioconductor package LipidR [111] with the following caveats: lipid concentrations were normalized to a normalization factor calculated by dividing the protein concentrations of each sample by the median of all sample protein concentrations. Concentrations were then multiplied by 1000 and log2 transformed for further analysis. To decrease false discovery rate, all *p*-values in omics results were corrected by using the Benjamini–Hochberg correction. *T*-test and ANOVA comparisons between groups were made using R package ggpubr (stat_compare_means function). PC plots were generated using R package factoextra. Statistical filtering for interactomics analysis was performed using SAINT and further refined with CRAPome [112].

### C-laurdan staining and imaging

C-Laurdan staining, imaging, and analysis were done according to [113]. In short, HUVECs grown on 4-well plate wells with a cover slip were incubated for 24 h with either 1:1000 of CpdG or DMSO. After that, incubation media were changed to FBS-free EGCM2 media with 5 μM of C-laurdan, and cells were incubated for 30 min at 37 °C 5% CO_2_, after which cells were washed twice with 1 X PBS. Washed cells were fixed with 4% PFA for 10 min at RT. PFA was removed and cells were washed twice with 1 X PBS. Cover slips were mounted with Mowiol:Dabco solution. C-Laurdan imaging was performed using a Leica Stellaris 8 Falcon/DLS inverted confocal microscope, equipped with HyD detectors and a 63X/1.40 HC PL APO CS2 oil objective, with an excitation of a 405 nm diode laser and emission spectra of 415–460 and 470–530 nm. Gains were kept constant during imaging. Generalized polarization (GP) values were calculated using ImageJ, and pseudocolored images were generated according to the described ImageJ macro [113].

### AMAXA transfection and quantification of D4H-mCherry phenotypes

AMAXA D4H-mCherry transfections were done according to manufacturer’s instructions (Lonza Bioscience, cat #: VPB-1002), by adding 3.1 μg of D4H-mCherry vector to the reaction. Transfected cells were seeded in 4-well plates with cover slips. Two hours after seeding, media were changed to normal EGCM2 media, and cells were incubated overnight. Transfected cells were incubated for another 24 h in 100 μL of media containing 10 μM CpdG or 1:1000 DMSO. Cells were fixed with 4% PFA in PBS and blocked in 1% BSA in PBS. Coverslips were mounted with Mowiol:Dabco:DAPI solution. Imaging was carried out on a Zeiss Observer.Z1 fluorescence microscope (Serial #: 3,834,002,509, Zeiss Group), with a 63 X immersion oil objective. Coverslips were mounted, and each microscope slide was blinded by obscuring the markings on microscope slide. One hundred cells were counted from each cover slip with the assistance of an Arduino microcontroller, the code can be found in a folder “threebuttoncounter” in the Open Science framework repository https://osf.io/jy5vu/.

## Supplementary Information

Additional file 1: Fig. S1: Metabolic activity (MTT-assay) in CpdG or DMSO treated and oex cells. Fig. S2: PCA-scores plots of omics results used in this study. Fig. S3: Dot plot of enriched WikiPathways in oexORP7 cells. Fig. S4: Volcano plot of differential expression results in oexORP7 cells. Fig. S5: Box plots of angiogenesis metrics in oexORP7 cells. Fig. S6: Box plots of total and free cholesterol levels in oexORP7 cells. Fig. S7: Box plots of cholesterol efflux fold change of oexORP7 cells compared to oexControl cells. Fig. S8: Box plot of log2 fold change distribution of different lipid classes in oexORP7 HUVECs. Fig. S9: Tile plot of log2 fold changes in oexORP7 cells compared to oexControl cells. Fig. S10: A box plot exhibiting lipid droplet metrics in oexORP7-cells compared to oexControl cells. Fig. S11: Western blotting results of both unphosphorylated and phosphorylated FAK and AKT1 as well as β-actin loading control in oexORP7 and control cells. Fig. S12: Full membrane images used to make Fig. 11. Fig. S13: Chemiluminescence images of CNR1 quantification in CpdG and DMSO treated cells. Fig. S14: Full membrane images used to make lower right section of Fig. S11 B. Fig. S15: Full membrane images used to make upper right section of Fig. S11 B. Fig. S16: Complete membrane images for oexORP7 and oexControl cells, used in Fig. 1C.

Additional file 2: Figs. S1-S8: GSEA plots for results from other databases shown on top of each plot. Transcriptomics results were from CpdG treated versus DMSO control cells. Figs. S9-S16: GSEA plots for results from other databases shown on top of each plot. Transcriptomics results were from oexORP7 treated versus control cells.

Additional file 3: Figs. S1–S26: Complete membrane images.

## Data Availability

Data has been added to Open Science Framework and can be found from the following link: https://osf.io/jy5vu/. All transcriptomics data has been uploaded to Gene Expression Omnibus under the accession number GSE261689. Any missing data or materials are made available upon reasonable request.

## Abbreviations

AA: Arachidonic acid
ABCG1: ATP-binding cassette subfamily G member 1
AKT1: RAC-alpha serine/threonine-protein kinase 1
ALOX: Lipoxygenase genes
ApoA1: Apolipoprotein A1
AvgP: Average probability
CNR1: Cannabinoid receptor 1
CE: Cholesteryl esters
Cer: Ceramides
D4H: Domain 4 (D4) of perfringolysin O
ECM: Extracellular matrix
FAK: Focal adhesion kinase
FC: Free cholesterol
FDR: False discovery rate
GABARAPL2: GABA type A receptor-associated protein like 2
HDL: High-density lipoprotein
HUVECs: Human umbilical vein endothelial cells
LC3B: Microtubule-associated proteins 1A/1B light chain 3B
log2FC: Log2 fold change
LPC: Lysophosphatidylcholines
LXR: Liver X receptor
MaxP: Maximum probability
MAM: Mitochondria-associated membrane
MCS: Membrane contact site
NES: Normalized enrichment score
nTPM: Transcripts per million
Oex: Over expression
OexControl: Overexpression construct of mycBirA tag
OexORP7: Overexpression construct of ORP7 with a mycBirA tag
ORP: Oxysterol-binding protein-related protein
OSBP: Oxysterol-binding protein
padj: Benjamini-Hochberg adjusted *p*-values
PCA: Principal component analysis
PDCA: Pancreatic ductal adenocarcinoma
PE: Phosphatidylethanolamine
PI: Phosphatidylinositol
PM: Plasma membrane
PS: Phosphatidylserines
SM: Sphingomyelin
TC: Total cholesterol
TG: Triacylglycerol
