# Supplementary material for: Functional omics of ORP7 in primary endothelial cells
Source: BMC Biol. 2024 Dec 18;22:292. doi: 10.1186/s12915-024-02087-6 (PMC11656939; doi:10.1186/s12915-024-02087-6)
Supplement: Supplementary file 1 — Additional file 1: Fig. S1: Metabolic activity (MTT-assay) in CpdG or DMSO treated and oex cells. Fig. S2: PCA-scores plots of omics results used in this study. Fig. S3: Dot plot of enriched WikiPathways in oexORP7 cells. Fig. S4: Volcano plot of differential expression results in oexORP7 cells. Fig. S5: Box plots of angiogenesis metrics in oexORP7 cells. Fig. S6: Box plots of total and free cholesterol levels in oexORP7 cells. Fig. S7: Box plots of cholesterol efflux fold change of oexORP7 cells compared to oexControl cells. Fig. S8: Box plot of log2 fold change distribution of different lipid classes in oexORP7 HUVECs. Fig. S9: Tile plot of log2 fold changes in oexORP7 cells compared to oexControl cells. Fig. S10: A box plot exhibiting lipid droplet metrics in oexORP7-cells compared to oexControl cells. Fig. S11: Western blotting results of both unphosphorylated and phosphorylated FAK and AKT1 as well as β-actin loading control in oexORP7 and control cells. Fig. S12: Full membrane images used to make Fig. 11. Fig. S13: Chemiluminescence images of CNR1 quantification in CpdG and DMSO treated cells. Fig. S14: Full membrane images used to make lower right section of Fig. S11 B. Fig. S15: Full membrane images used to make upper right section of Fig. S11 B. Fig. S16: Complete membrane images for oexORP7 and oexControl cells, used in Fig. 1C. [file 12915_2024_2087_MOESM1_ESM.docx]

**Functional omics of ORP7 in primary endothelial cells**

Juuso H. Taskinen, Minna Holopainen, Hanna Ruhanen, Miesje van der Stoel, Reijo Käkelä, Elina Ikonen, Salla Keskitalo, Markku Varjosalo, Vesa M. Olkkonen

Additional File 1

As we explained in the main article, we decided to keep most of the results we obtained for overexpression samples separate from the inhibitor treated cell results. We will use the same numbering scheme for subsections and figures to keep cross-referencing as easy as possible. Therefore, there might not only be gaps in the subsection numbering, but similar gaps can also appear in figure numbering.

3.2. Quality control of over expression treatment, and omics results.

We also performed PCA on the overexpressed cells (oexControl and oexORP7) omics data and these results are shown in Figure S2 panels C-E (appearing after the PCA on DMSO- and CpdG-inhibited cells in panels A and B). Transcriptomics data show clear separation along the first principal component, marked as dimension 1 (Dim1), explaining the largest part of the data variation, whereas the separation power of the second largest dimension 2 (Dim2) is clearly smaller. Lipidomics data shows more random spread along both dimensions than the transcriptomics data, even though the group centers for the two groups separate along the diagonal.


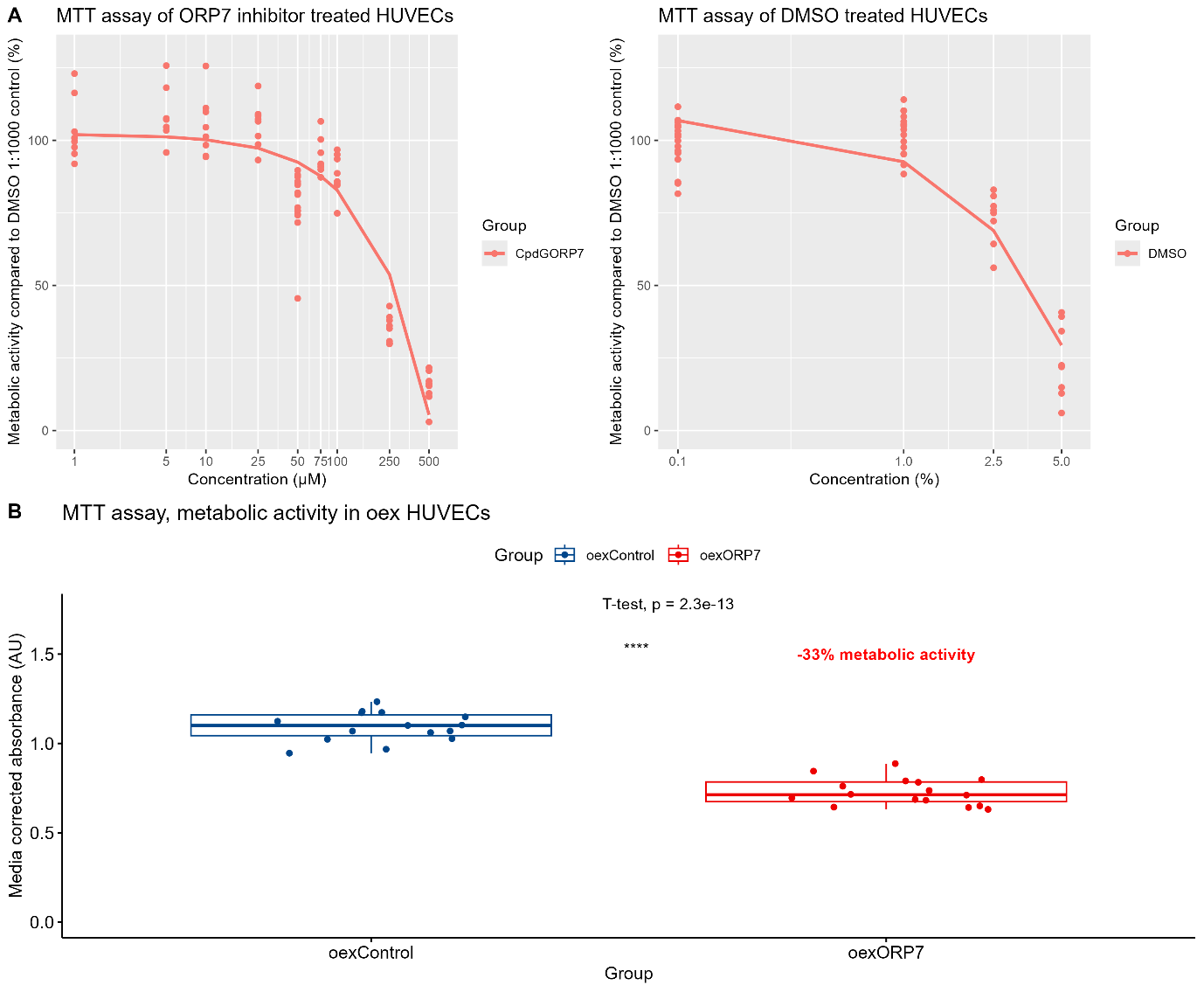


Figure S1. Metabolic activity (MTT-assay) in CpdG or DMSO treated and oex cells. A) Y-axis represents the % of metabolic activity compared to vehicle control (1:1000 DMSO), and the X-axis depicts either the concentration of CpdG in µM or DMSO in v/v-%. N = 8 per concentration. B) Metabolic activity in oexORP7 or oexControl HUVECs, Y-axis represents absorbance in arbitrary units, and X-axis each group measured. In red is displayed the calculated reduction in metabolic activity in oexORP7 cells as compared to oexControl cells. N = 14 per group. P-values were determined using Student’s T-test.


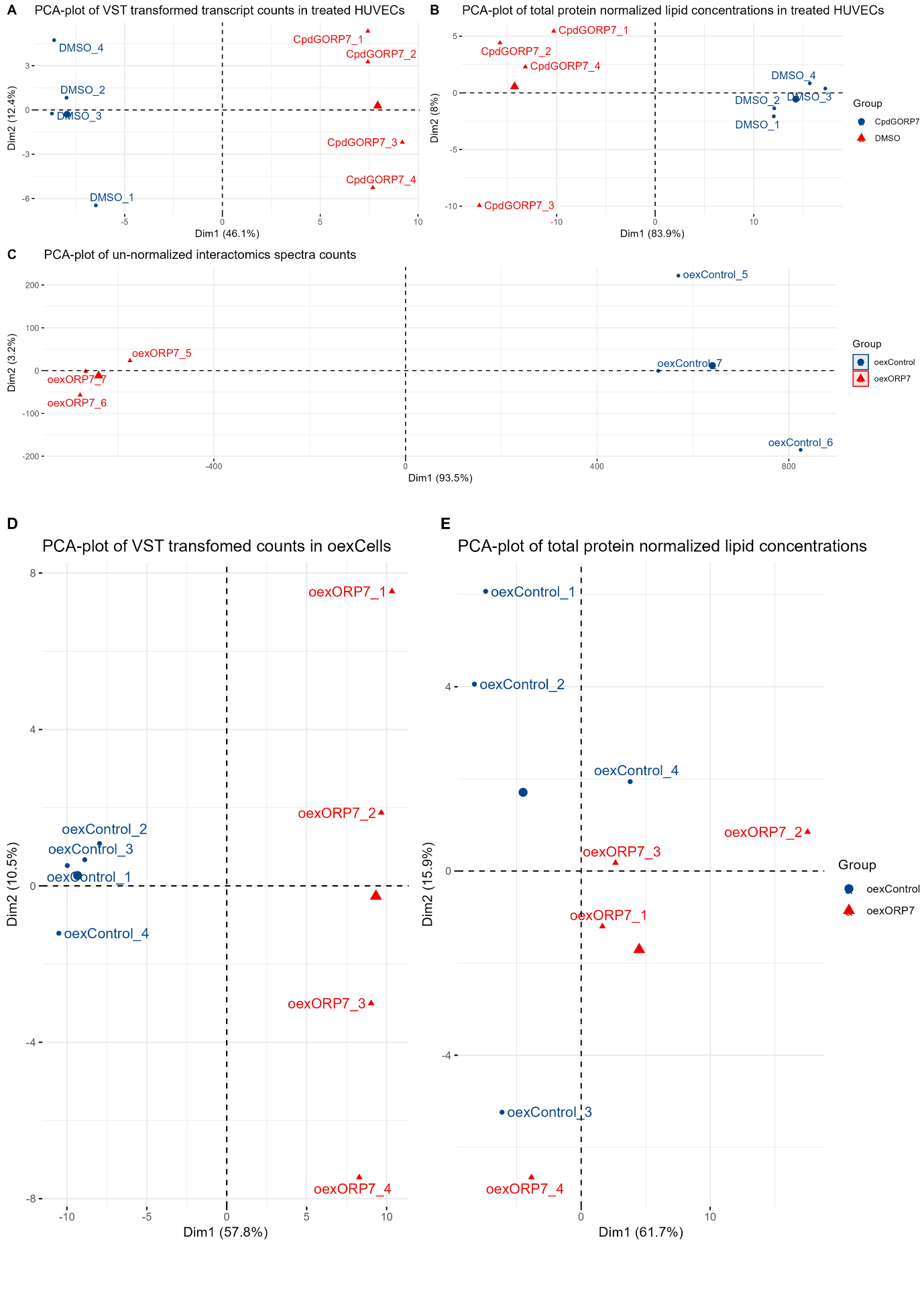
Figure S2. PCA-scores plots of omics results used in this study. All plots show controls (DMSO or oexControl) in blue and CpdG inhibition or ORP7 overexpression in red. The axes show the two largest principal components (marked Dim1 and Dim2) where percentage in brackets is the amount of variation explained by the principal component. A) PCA-plot of variance stabilizing transformed transcript counts in CpdG- or DMSO-treated HUVECs. B) Total protein normalized lipid concentration in CpdG- or DMSO-treated HUVECs. C) PCA-plot of un-normalized unique spectra counts in proximity based interactomics samples. D) PCA-plot of variance stabilizing transformed transcript counts in oexORP7 or oexControl HUVECs. E) Total protein normalized lipid concentration in oexORP7 or oexControl HUVECs. In all plots, except E, the samples of a study group are shown to group together along the largest principal component (Dim1), with additional within-group spread along the second largest principal component (Dim2). N = 4.

3.3 Transcriptomics reveals multifaceted changes in inhibitor treated HUVECs.

We performed gene set analysis on the oex-cell samples, but the results showed relatively little significant changes in any gene sets, suggesting that expression changes are somewhat random. For those who are not familiar with gene set analysis, a simplified explanation of how gene sets are considered significant is as follows: Significance of a gene set is based on how genes within a gene set cluster in a, for example, log2 fold change ranked list of genes. This means that if genes in a gene set cluster more towards top, middle or bottom of the list the gene set is considered significant, whereas if genes are clustered randomly in the list, the set is not significant. In Figure S3.1 a dot plot of enriched gene sets from Wikipathways is shown, which demonstrates on the X-axis the normalized enrichment score (NES) of each gene set shown on the Y-axis. The color of each dot represents the Benjamini Hochberg adjusted p-value for each gene set. Figure S3.1, shows some similarities to the results in treated cells although the amount of significantly altered pathways is much lower. For example, on the left facet pathways related to cancer are shown and on the right facet pathways related to cholesterol synthesis are visible. Most notably inflammation related sets are shown in the left facet as well as cancer related sets in the right facet which is opposed to what we showed for the treated cells. Other databases show very few or no significantly altered pathways and these figures are shown as Figures S22-S29, which depict similar dot plots to Figure S6.


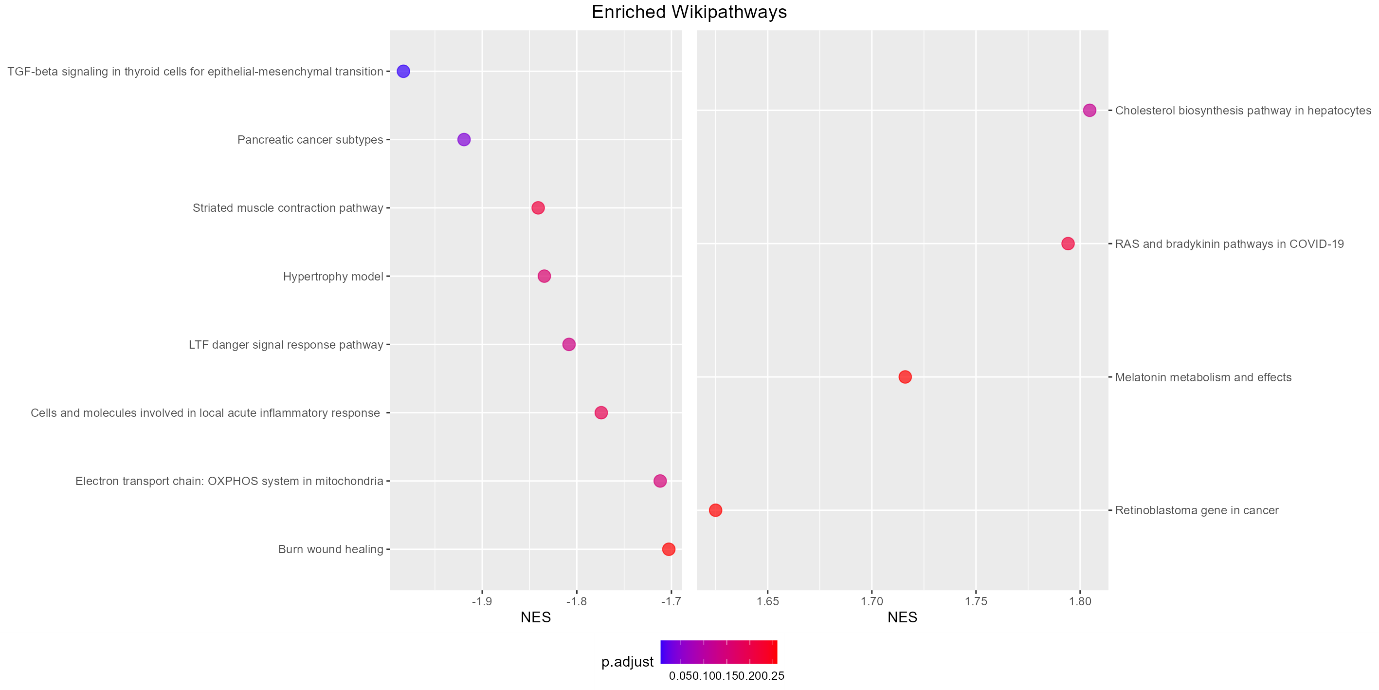


Figure S3. Dot plot of enriched Wikipathways in oexORP7 cells. Y-axis represents enriched gene sets, and X-axis the normalized enrichment score for each gene set, where a negative value indicates more downregulated genes and a positive value more upregulated genes. The color of each dot represents the Benjamini Hochberg adjusted p-value for each gene set.

Gene set analysis is not the end all be all functional analysis, however, it is insightful when investigating the individual gene changes. The most notable difference between oex-cells and CpdG treated cells is that genes in the oex-cells had a larger log2 fold change in both directions. Although these changes seem random based on gene set analysis, some insight could still be made. These changes are exhibited in Figure S4.


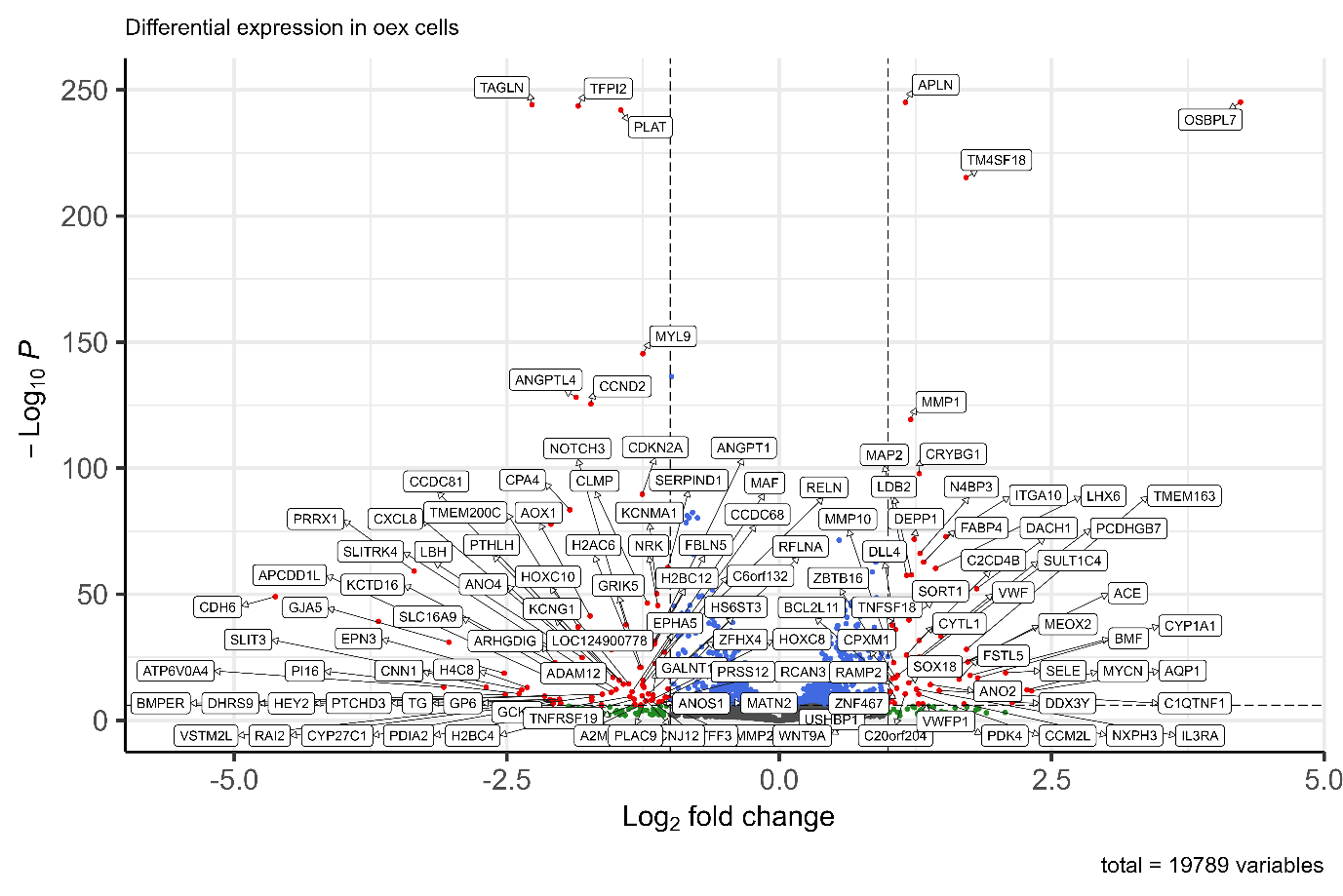


Figure S4. Volcano plot of differential expression results in oexORP7 cells. X-axis represents log2 fold change and Y-axis -log10 adjusted p-value. Labeled red dots show genes that have an absolute log2 fold change of 1 or higher and are significantly altered, blue dots have an absolute log2 fold change which is lower than 1 and are significantly altered, grey dots are genes that have not been significantly altered are shown between and below dashed lines.

We can see that OSBPL7 has had the larger positive fold change compared to oexControl and with a large significance value which serves as a good quality control metric for our overexpression construct in general. There are some similarities between the CpdG inhibited cells and oex-cells in the overexpressed genes, mainly: CYP1A1 ITGA10, SELE and SERPIND1 at the downregulated genes.

3.4 Manipulated HUVECs have reduced angiogenic capacity.

OexORP7 and oexControl cells were plated on a matrix which induces angiogenesis in HUVECs, to measure how the overexpression of ORP7 affects angiogenesis. These results are shown in Figure S5, where multiple angiogenesis metrics have been plotted in the oex-cells. All Y-axes represent different angiogenic metrics whereas all x-axes show each group measured. In all statistics, the calculated oexControl cells were the reference group.


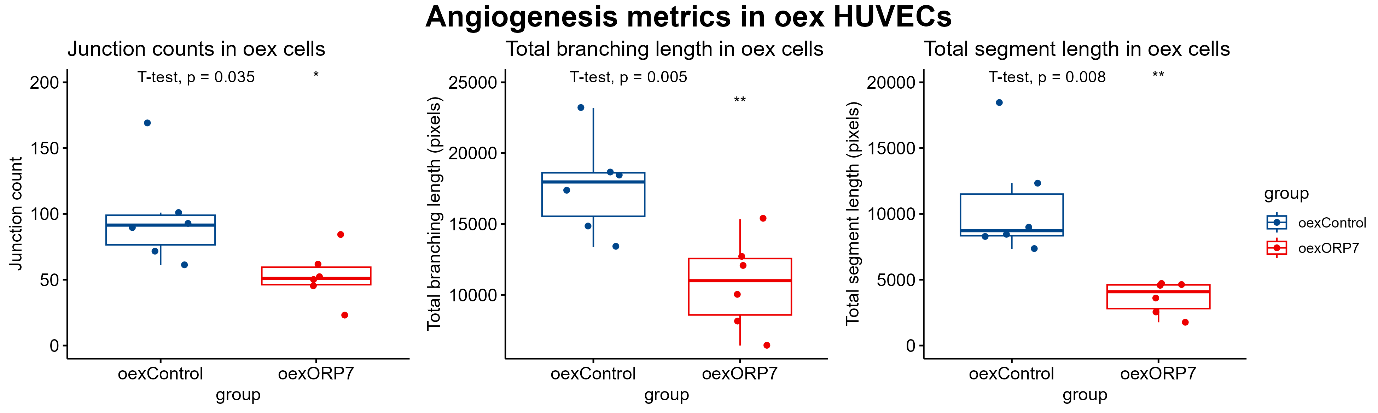


Figure S5. Box plots of angiogenesis metrics. In all plots the Y-axis represents each angiogenic metric and X-axis the group measured. Blue represents the oexControl cells and red indicates oexORP7 cells. The oexORP7 cells had statistically significantly (Students T-test) reduced values for all angiogenetic metrics.

As exhibited in Figure S5. overexpression of ORP7 reduced angiogenetic capacity in all metrics measured and a similar manner to inhibition of ORP7 but does not block it entirely.

3.5 Lipidome of manipulated HUVECs is significantly altered.

We also measured similar lipidomic metrics for the oexControl and oexORP7 cells as for the CpdG- or DMSO-treated cells. In contrast to the CpdG treated cells, we were able to detect a small reduction in free cholesterol levels in the oex-cells, but otherwise these results are the same as shown in Figure S6.


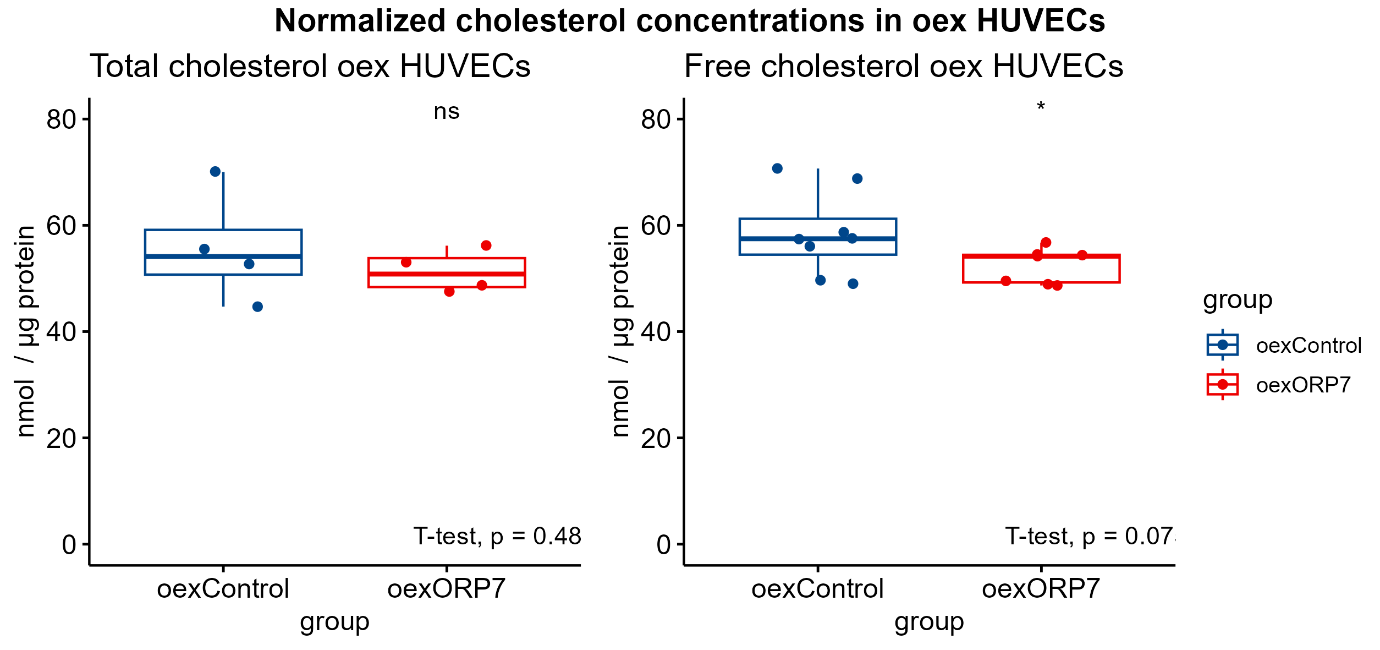


Figure S6. Box plots of total and free cholesterol levels, where the Y-axis represents protein normalized cholesterol concentrations and X-axis each group measured. Blue color represents the oexControl and red depicts oexORP7-cells. A significant but slight decrease in mean free cholesterol levels is visible. P-values were determined using Student’s T-test.


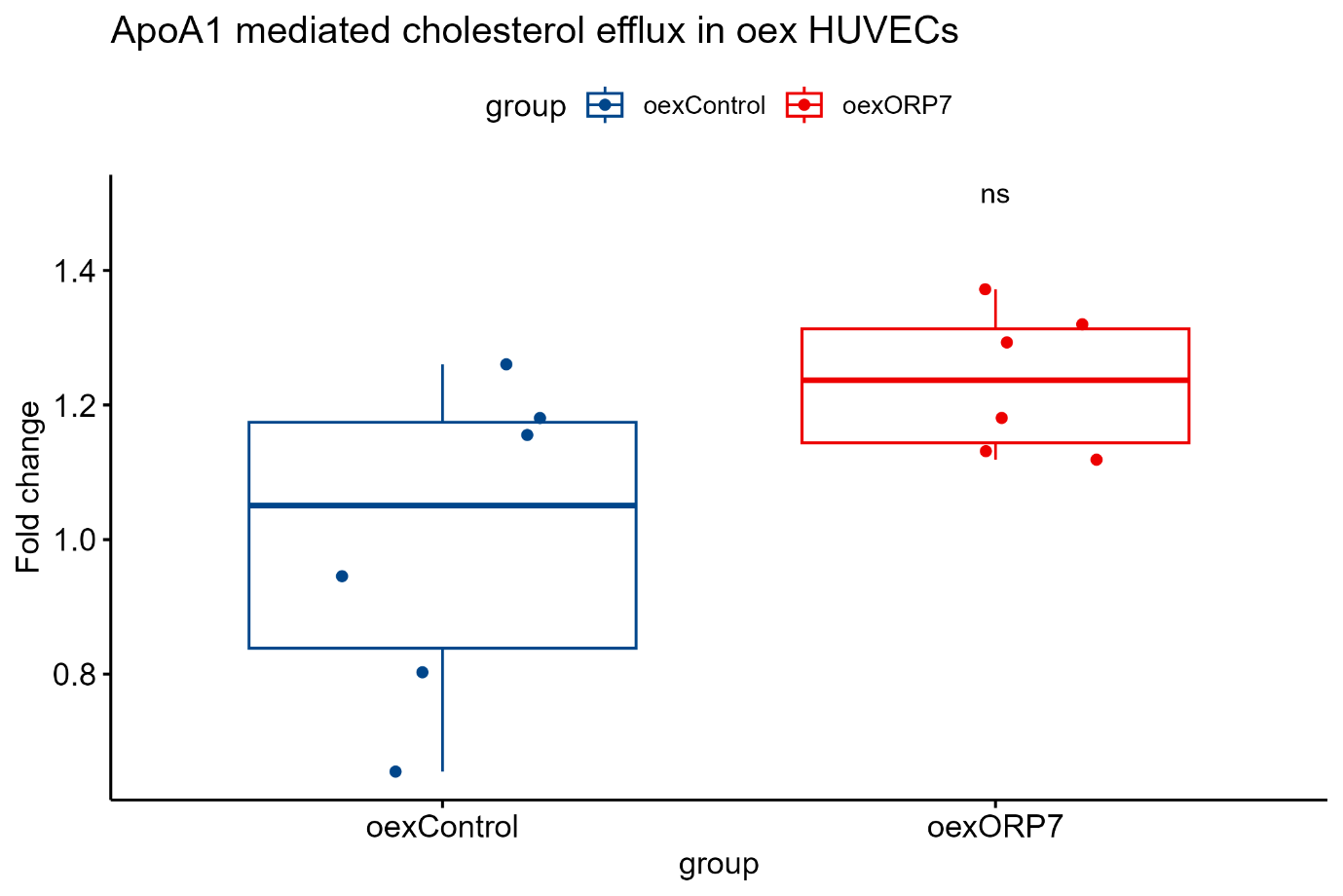


Figure S7. Box plots of cholesterol efflux fold change of oexORP7 cells compared to oexControl cells. A slight but non-significant increase in ApoA1 mediated cholesterol efflux is visible. P-values were determined using Student’s T-test and ANOVA.

ApoA1 mediated cholesterol efflux showed a slight increase in oexORP7 cells but this change was statistically not significant as shown in Figure S7

Lipidomic analysis of oexORP7 cells showed a similar but more muted decrease in cholesteryl ester (CE) and a larger drop in phosphatidylserine (PS) compared to CpdG inhibited cells, as well as an increase in phosphatidylcholine ether species (PC-O) in oexORP7 cells as compared to CpdG treated cells as shown in Figure S8


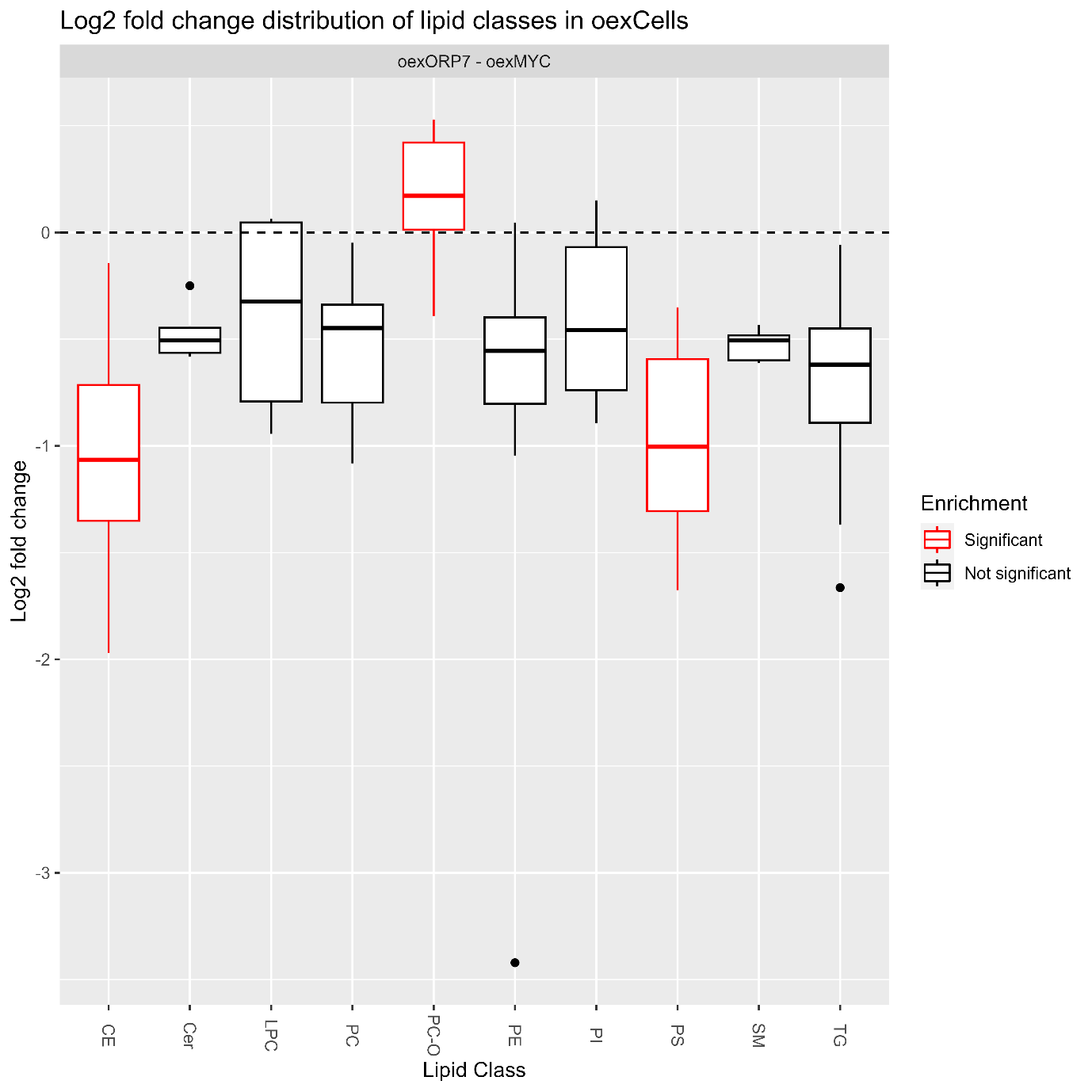


Figure S8. Box plot of log2 fold change distribution of different lipid classes in oexORP7 HUVECs. The Y-axis represents log2 fold change and X-axis each lipid class. Statistically significantly altered classes are shown in red. OexORP7 cells show significant changes in total concentrations of cholesteryl ester (CE), phosphatidylcholine ether species (PC-O) and phosphatidylserine (PS) when compared to oexControl cells.  The lipid classes that did not show any change were ceramide (Cer), lysophosphatidylcholine (LPC), phosphatidylcholine diacyl species (PC), phosphatidylethanolamine (PE), phosphatidylinositol (PI), sphingomyelin (SM) and triacylglycerol (TAG).


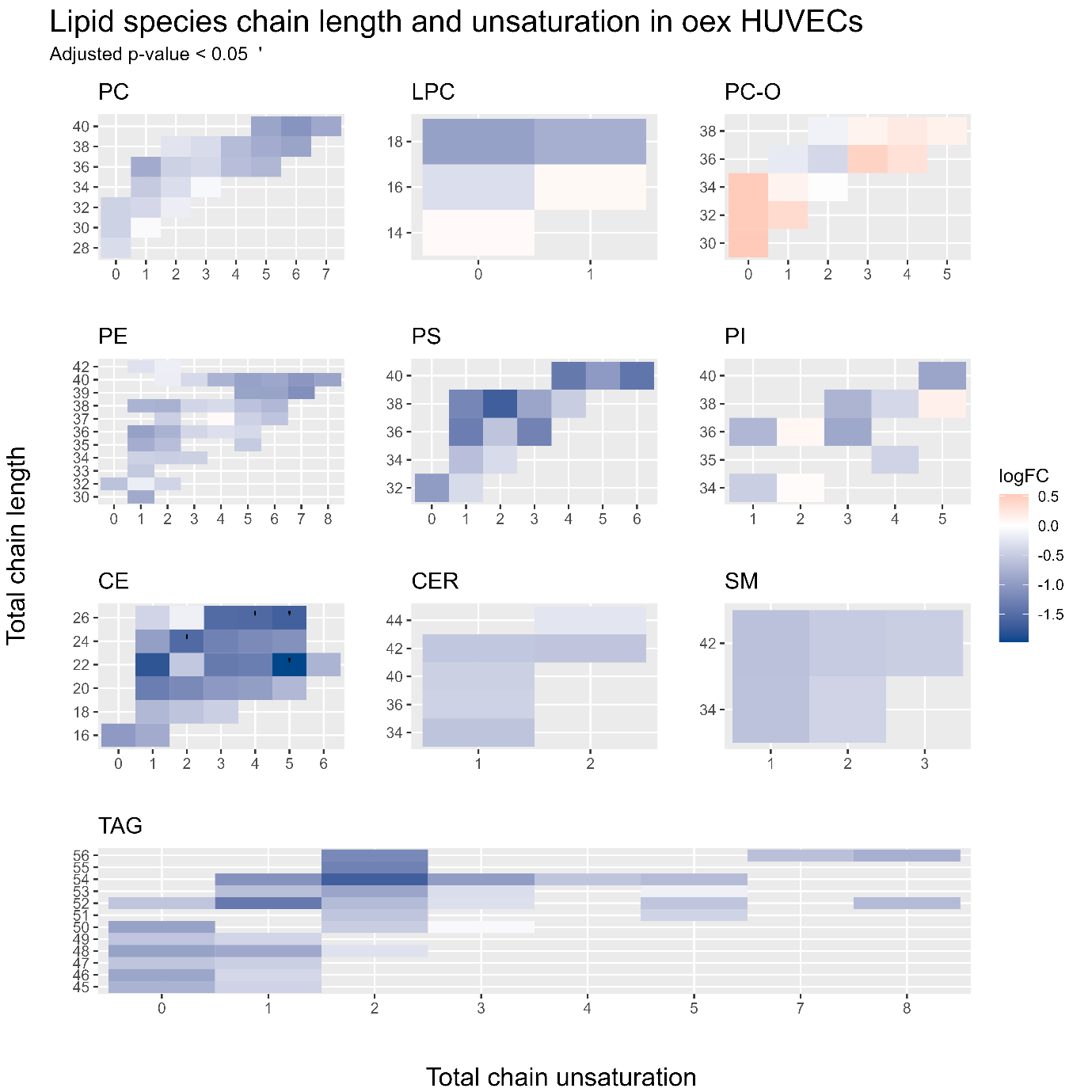


Figure S9. Tile plot of log2 fold changes in oexORP7 cells compared to oexControl cells. Each facet shows a different lipid class (abbreviations as in Figure S8), where the Y-axis represents the total chain length, the X-axis total chain unsaturation and each tile depicts a different lipid species. Each tile is colored according to the log2 fold change of each species, where orange represents an increase in and blue a decrease, each statistically significantly altered species has been marked with a dot on the tile.

Even though CEs, PS and PC-Os showed significant changes as a group, Figure S9 clearly shows that the changes in individual lipids are not significant in any other class except for CEs, where alterations are concentrated to only a few lipid species.


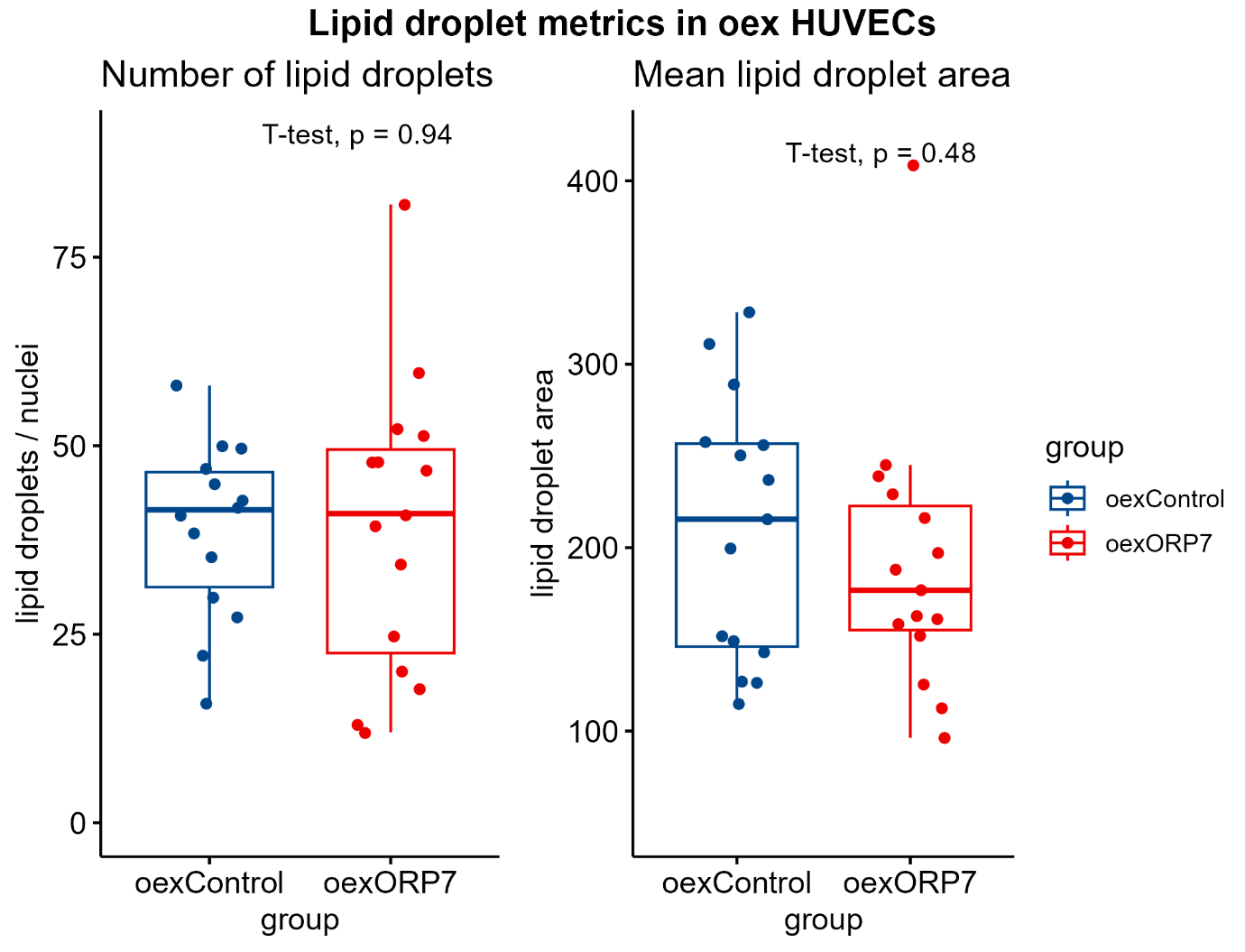


Figure S10, A box plot exhibiting lipid droplet metrics in oexORP7-cells (red) compared to oexControl cells (blue). Left and right facets show mean lipid droplet amount and mean lipid droplet area respectively. OexControl is shown in blue. No significant changes can be seen in either metric.

Lipid droplets showed no significant changes between the oexORP7 and oexControl cells in either the amount of lipid droplets or the mean area of lipid droplets as shown in Figure S10.


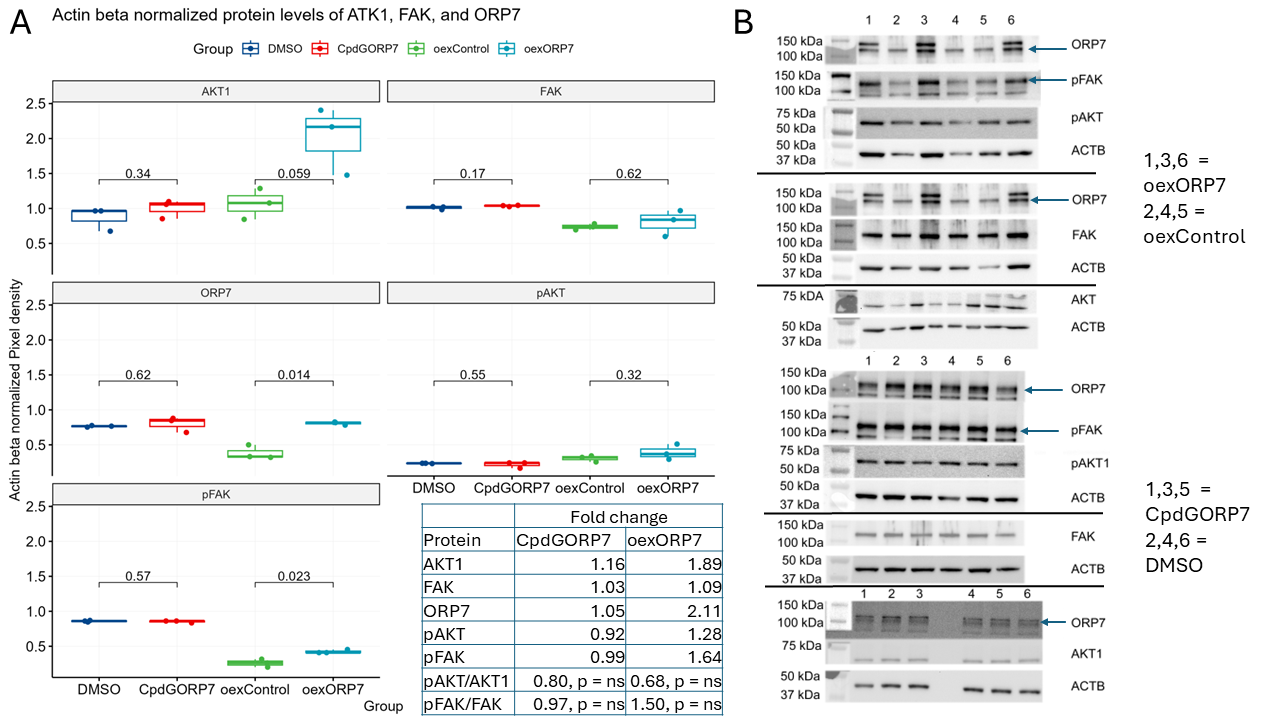


Figure S11. Western blotting results of both unphosphorylated and phosphorylated FAK and AKT1 as well as β-actin loading control. A) Box plots and table quantifying protein levels of AKT1, FAK and ORP7 and their fold change, where each facet shows the levels for different proteins, and pairwise t-test p-values shown above brackets as a comparison to DMSO and oexControl in CpdG treated and ORP7 overexpressing cells, respectively. The table also exhibits pAKT1/AKT1 and pFAK/FAK ratios and their statistical significance (N = 3). B) Western blots where sample types are shown on top of each lane and each row shows the protein detected. The left-most lane depicts the protein standard from composite images, whereas other lanes are from chemi-illuminance images. The top panel shows these results for oexORP7 and oexControl cells and bottom panel for CpdG and DMSO treated cells. Lysates are from one experiment and arrows point to the wild type protein annotated on the right side of each blot where two or more bands are visible. Black lines separate different membranes.


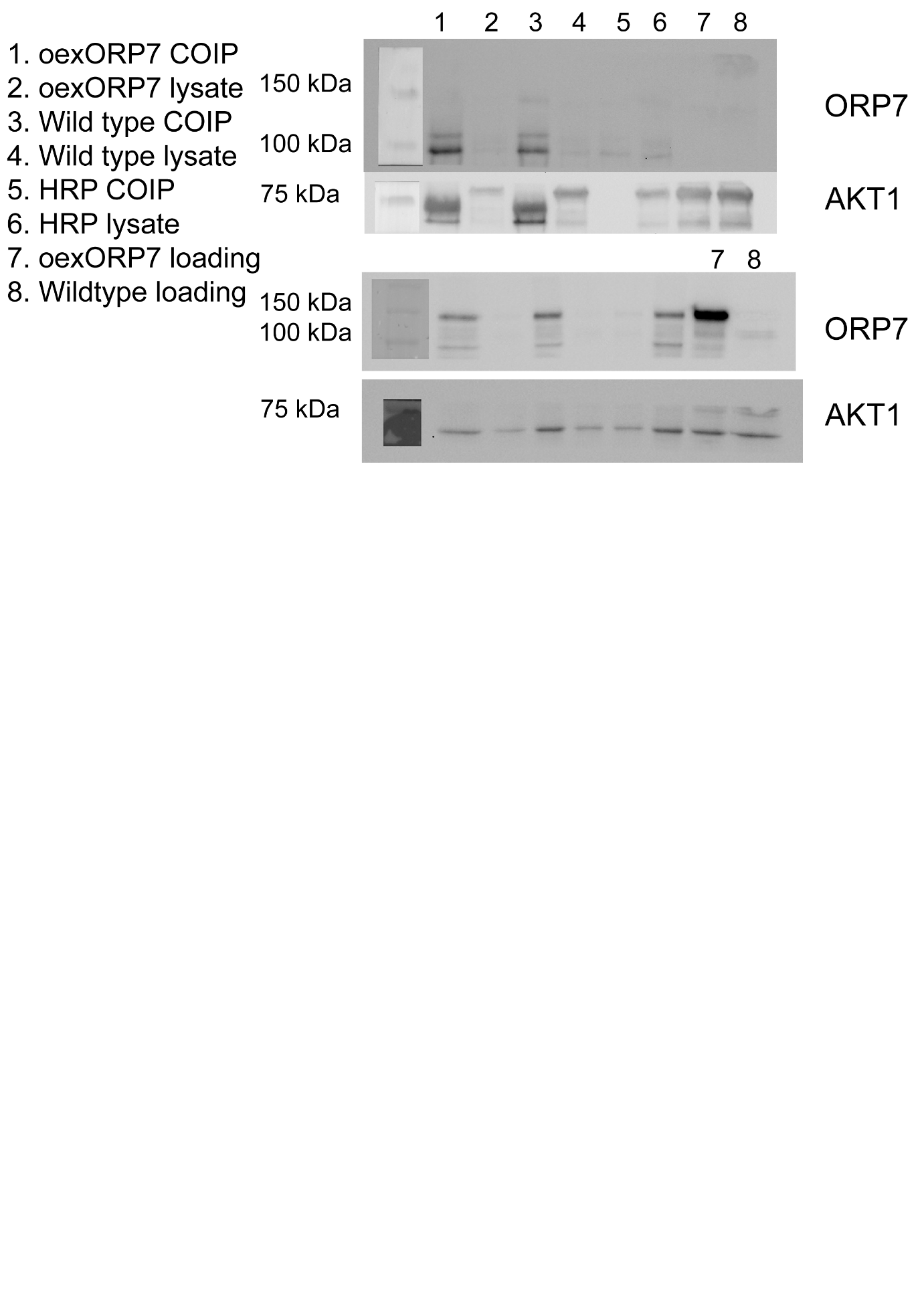
Figure S12. Full membrane images used to make figure 11


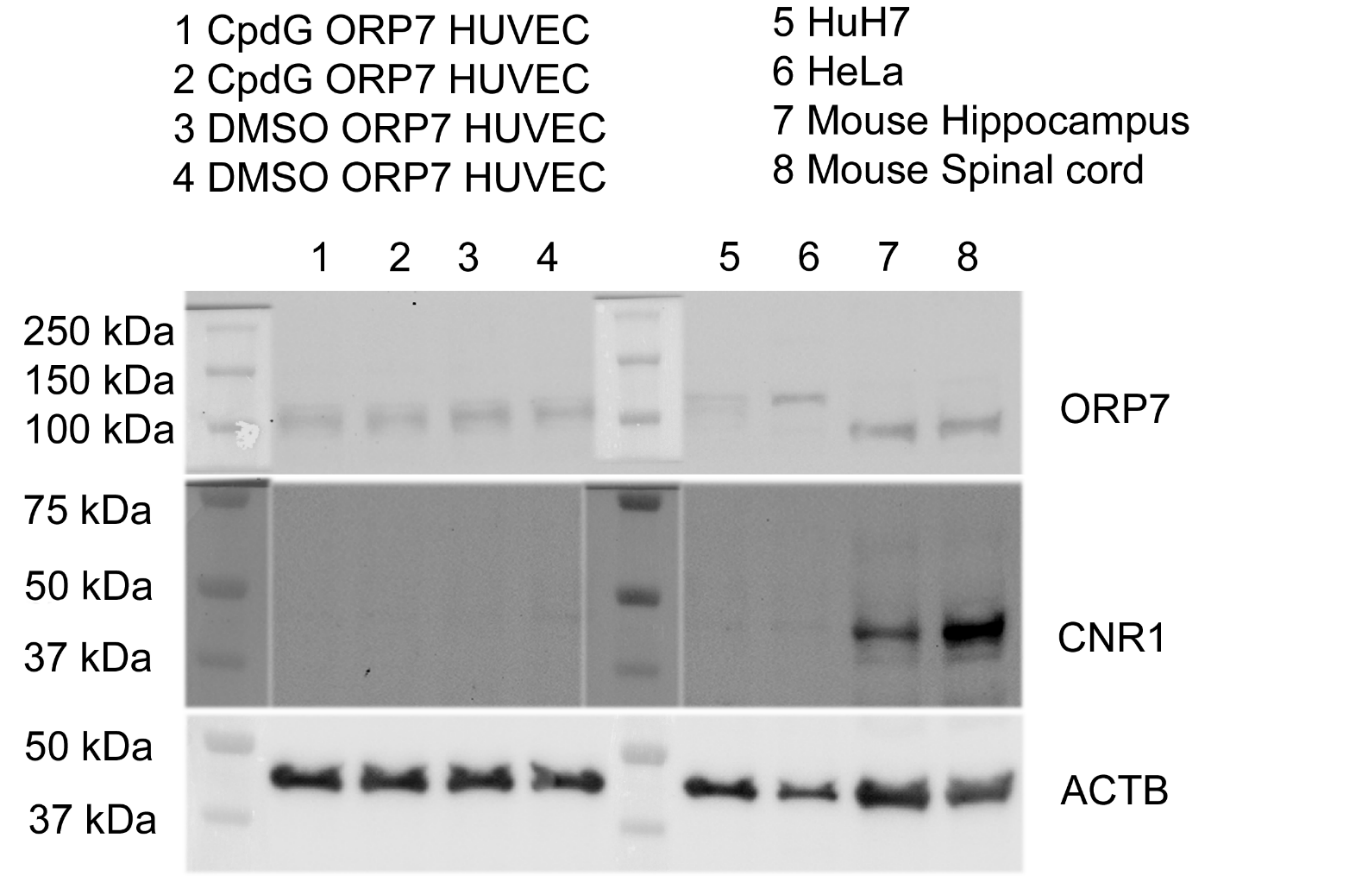


Figure S13. Chemiluminescence images of CNR1 quantification in CpdG and DMSO treated cells. The samples are identified at the top, molecular mass information displayed on the left, and each protein probed on the right. Mouse tissue samples shown in the right-most lanes show strong bands whereas there are no visible bands in the human cell lysates.


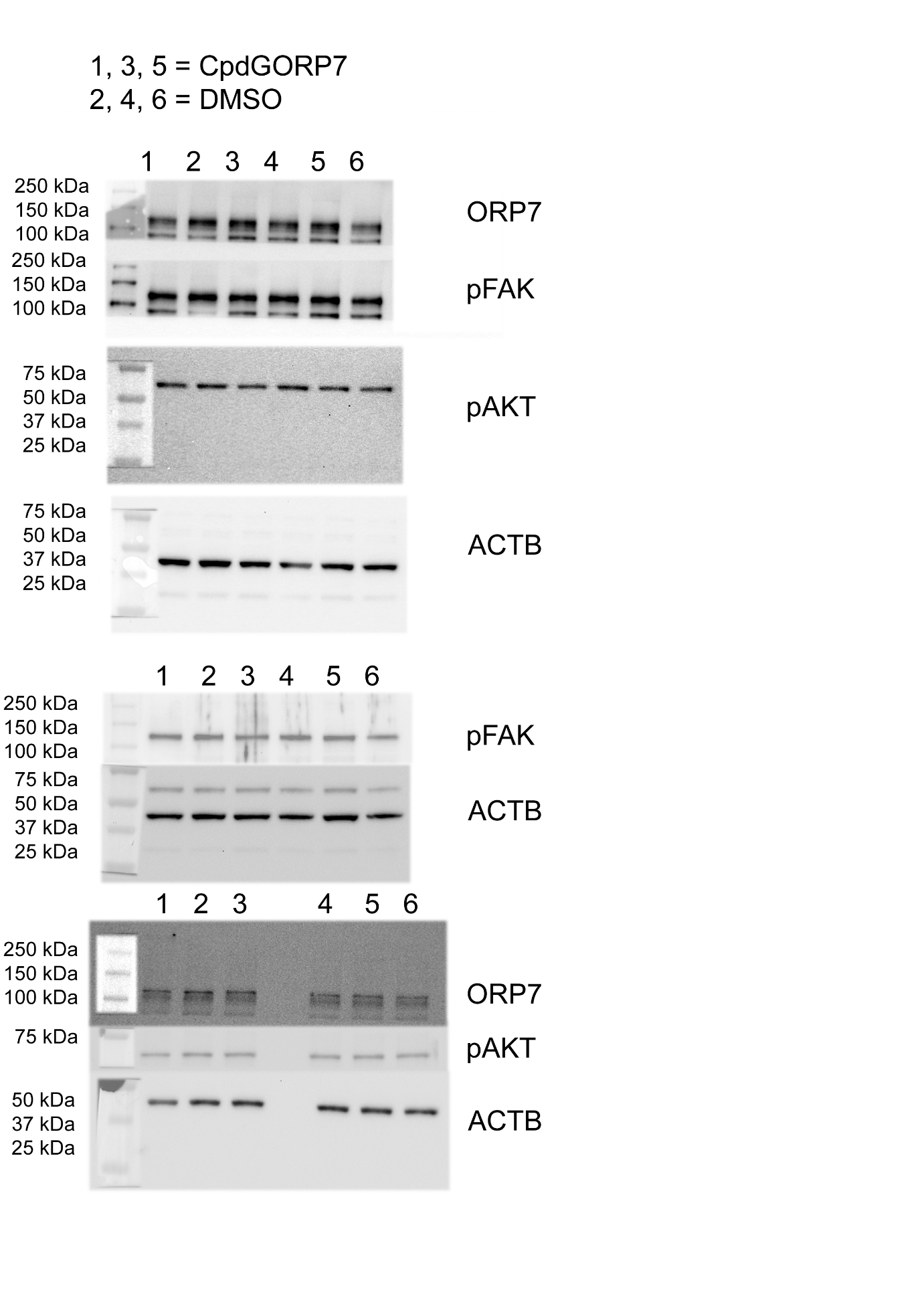


Figure S14. Complete membrane images for CpdG or DMSO treated cells, used in Figure S11, where protein ladders are from composite images and other lanes are from chemi-illuminance images. Sample order shown on the top of the image.


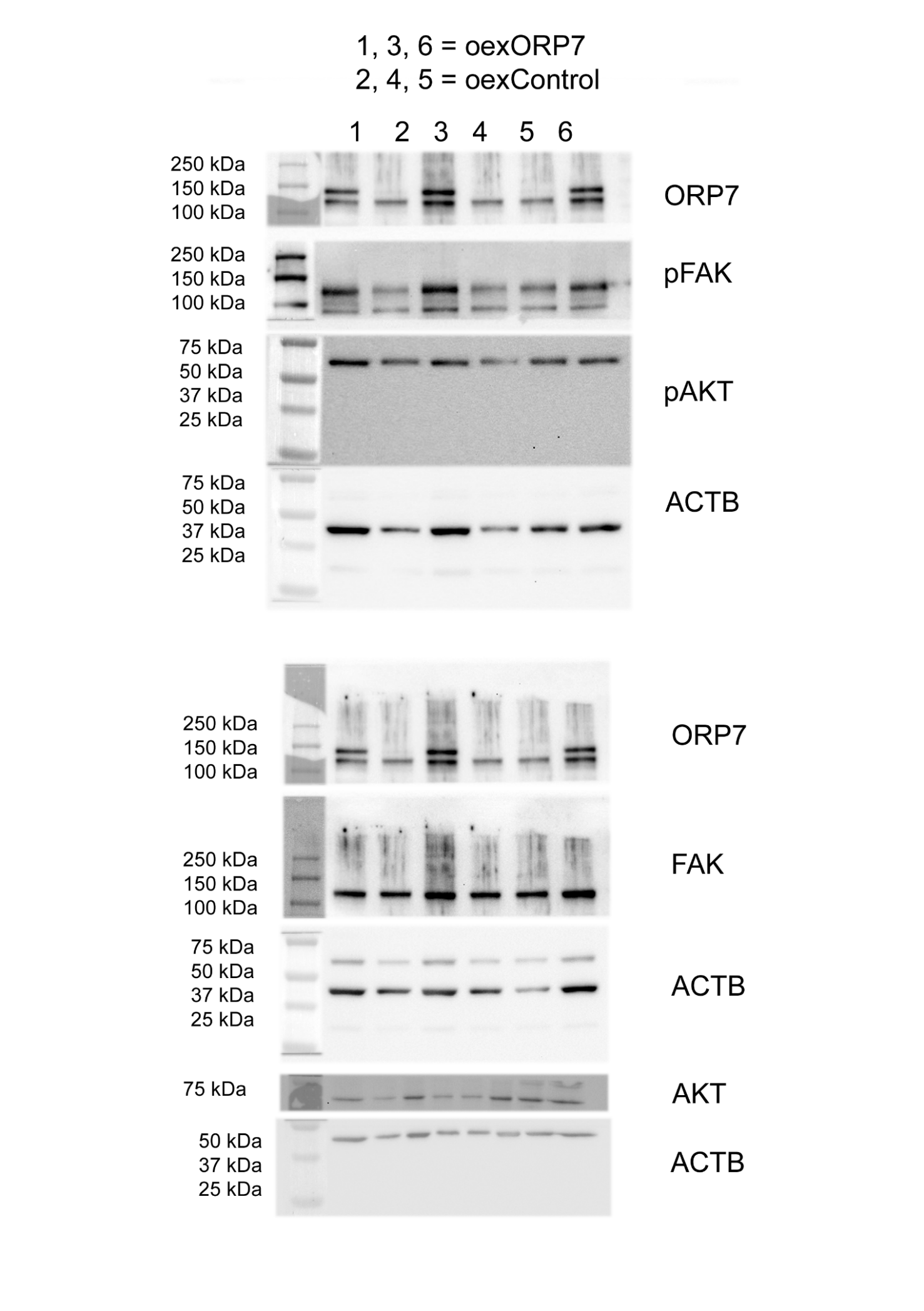


Figure S15. Complete membrane images for oexORP7 and oexControl cells, used in Figure S11, where protein ladders are from composite images and other lanes are from chemi-illuminance images. Sample order shown on the top of the image.


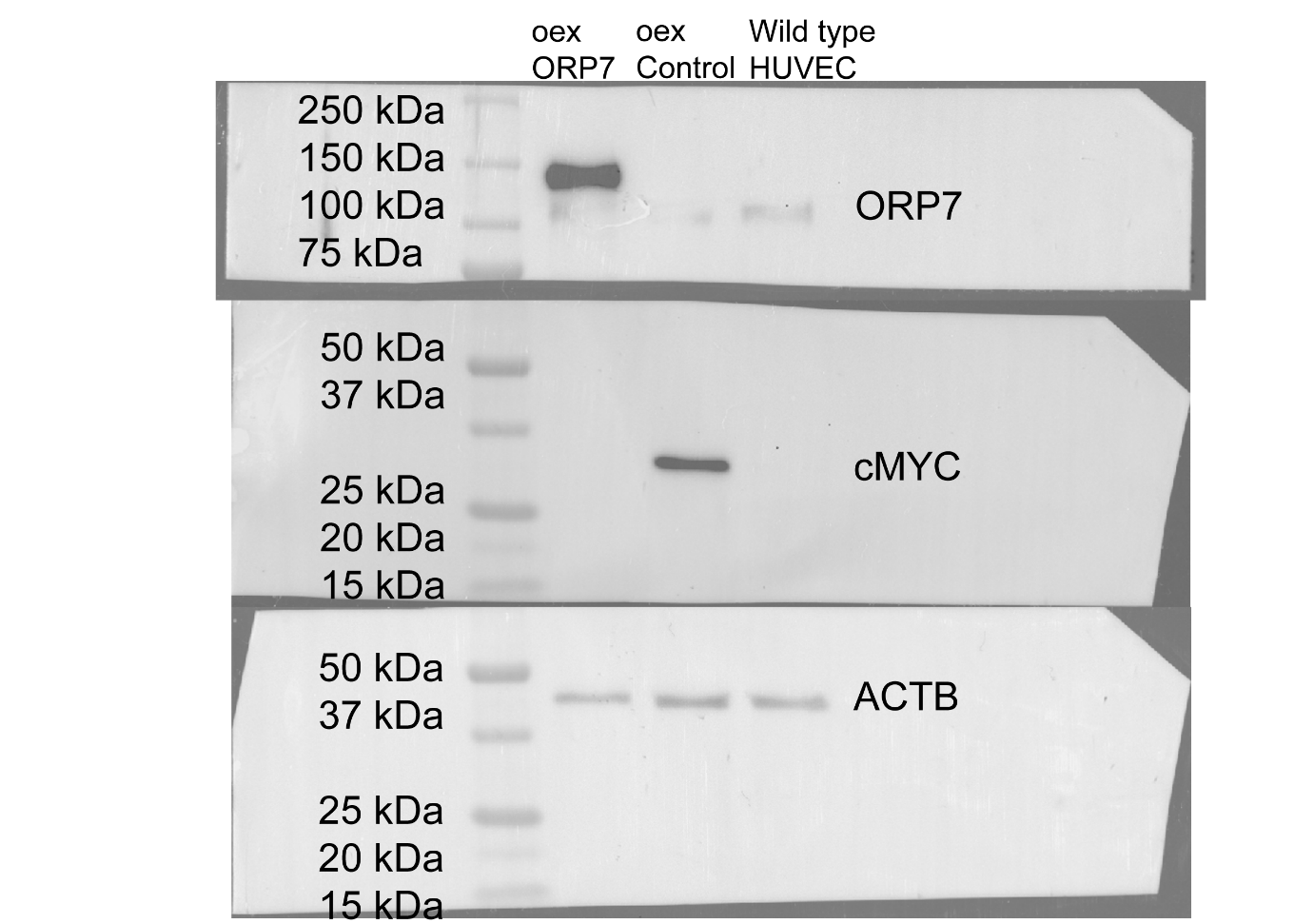


Figure S16. Full membrane images used to make Figure 1 C
