# Supplementary material for: Functional omics of ORP7 in primary endothelial cells
Source: BMC Biol. 2024 Dec 18;22:292. doi: 10.1186/s12915-024-02087-6 (PMC11656939; doi:10.1186/s12915-024-02087-6)
Supplement: Supplementary file 2 — Additional file 2: Figs. S1-S8: GSEA plots for results from other databases shown on top of each plot. Transcriptomics results were from CpdG treated versus DMSO control cells. Figs. S9-S16: GSEA plots for results from other databases shown on top of each plot. Transcriptomics results were from oexORP7 treated versus control cells. [file 12915_2024_2087_MOESM2_ESM.docx]

GSEA results on inhibitor treated cells.
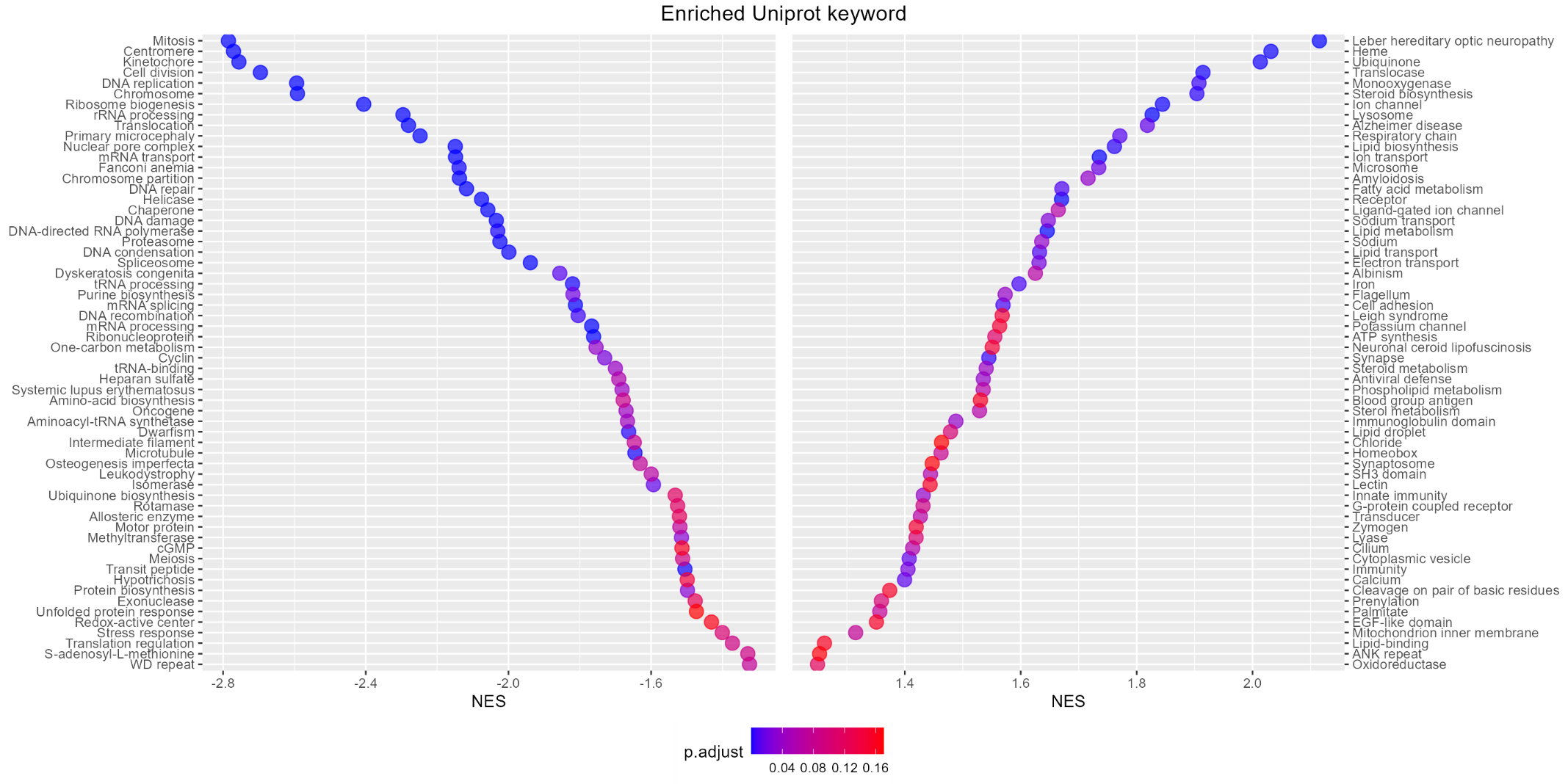
Figure S1


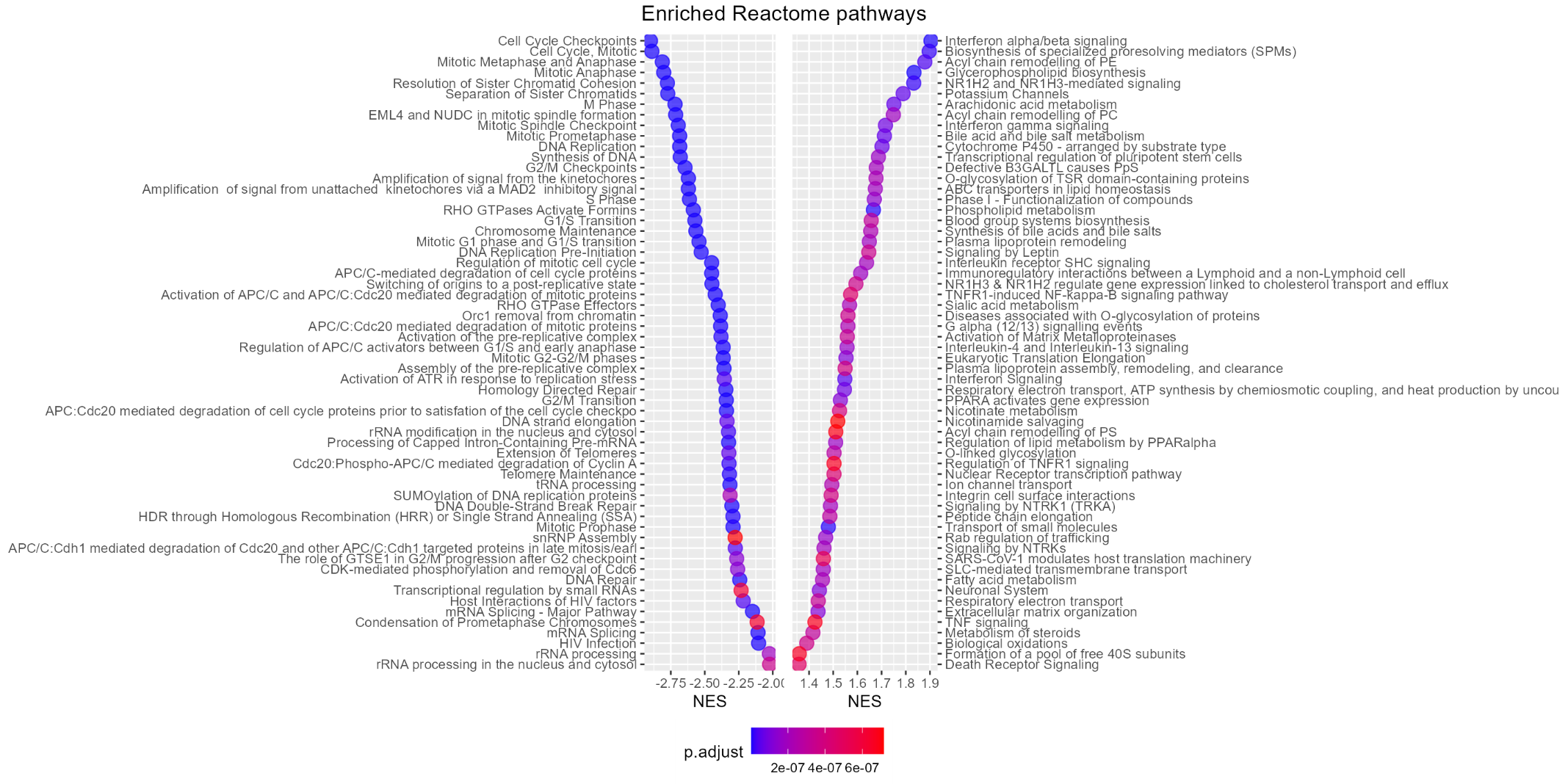
Figure S2
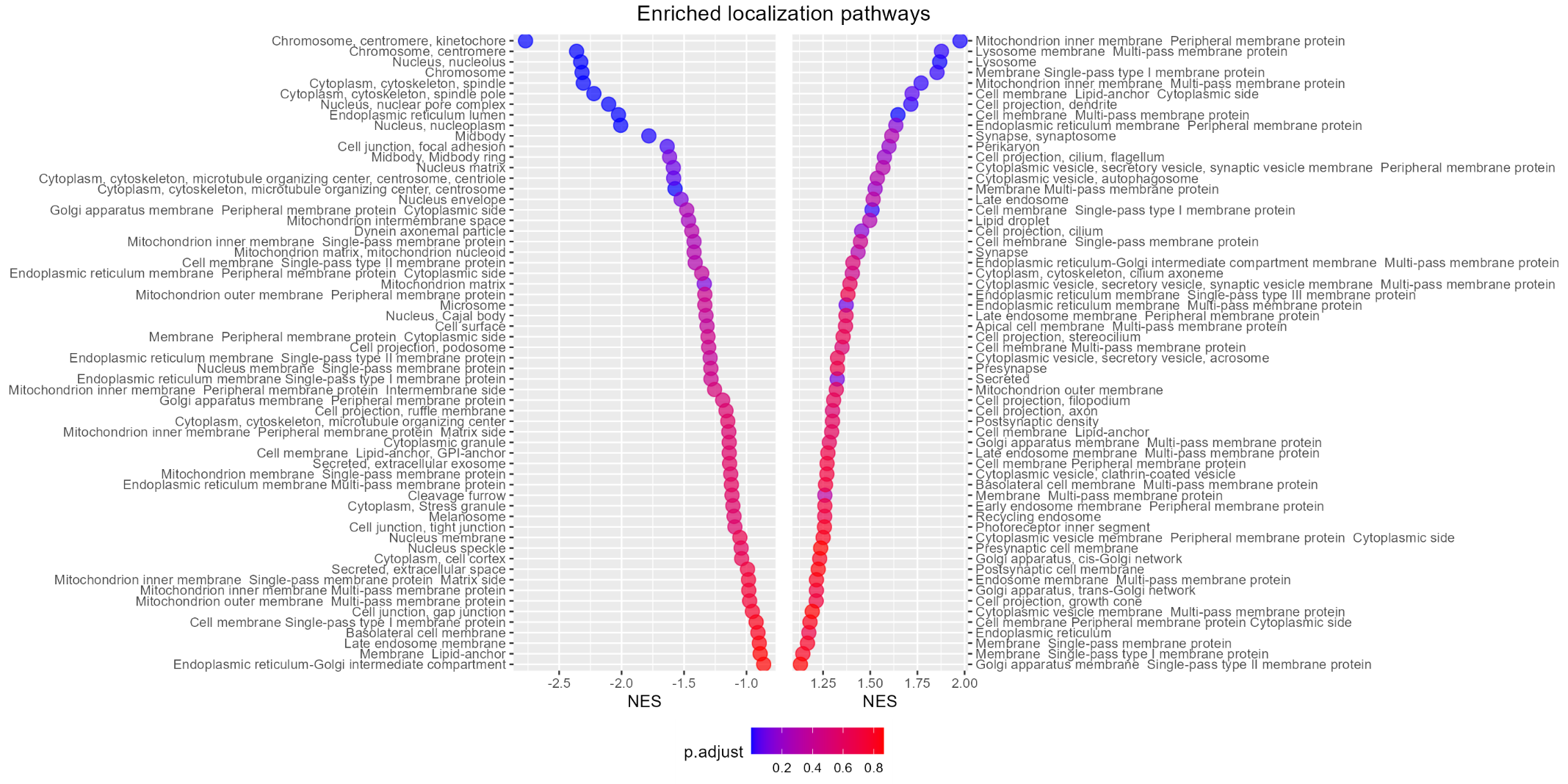
Figure S3
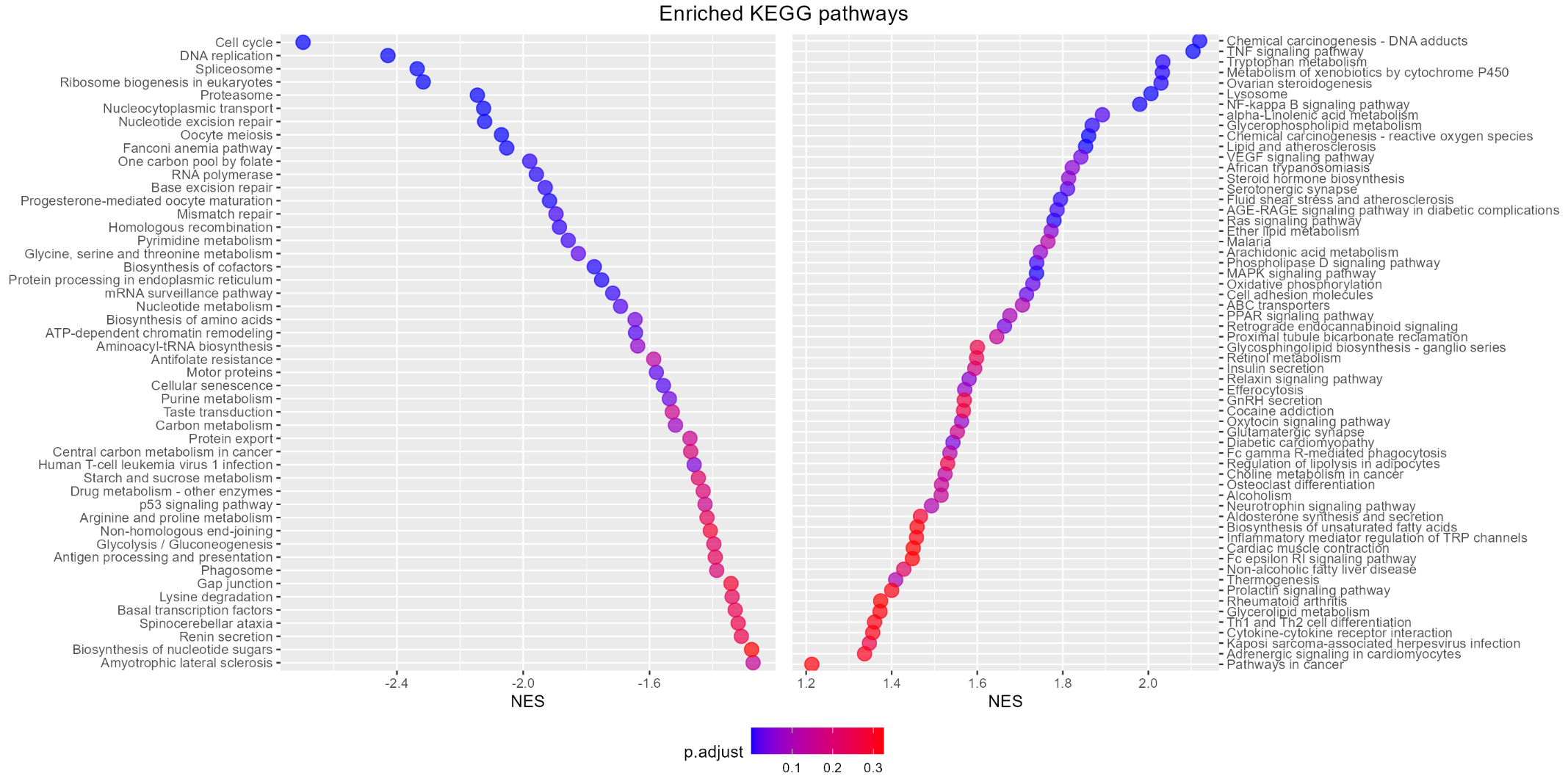
Figure S4
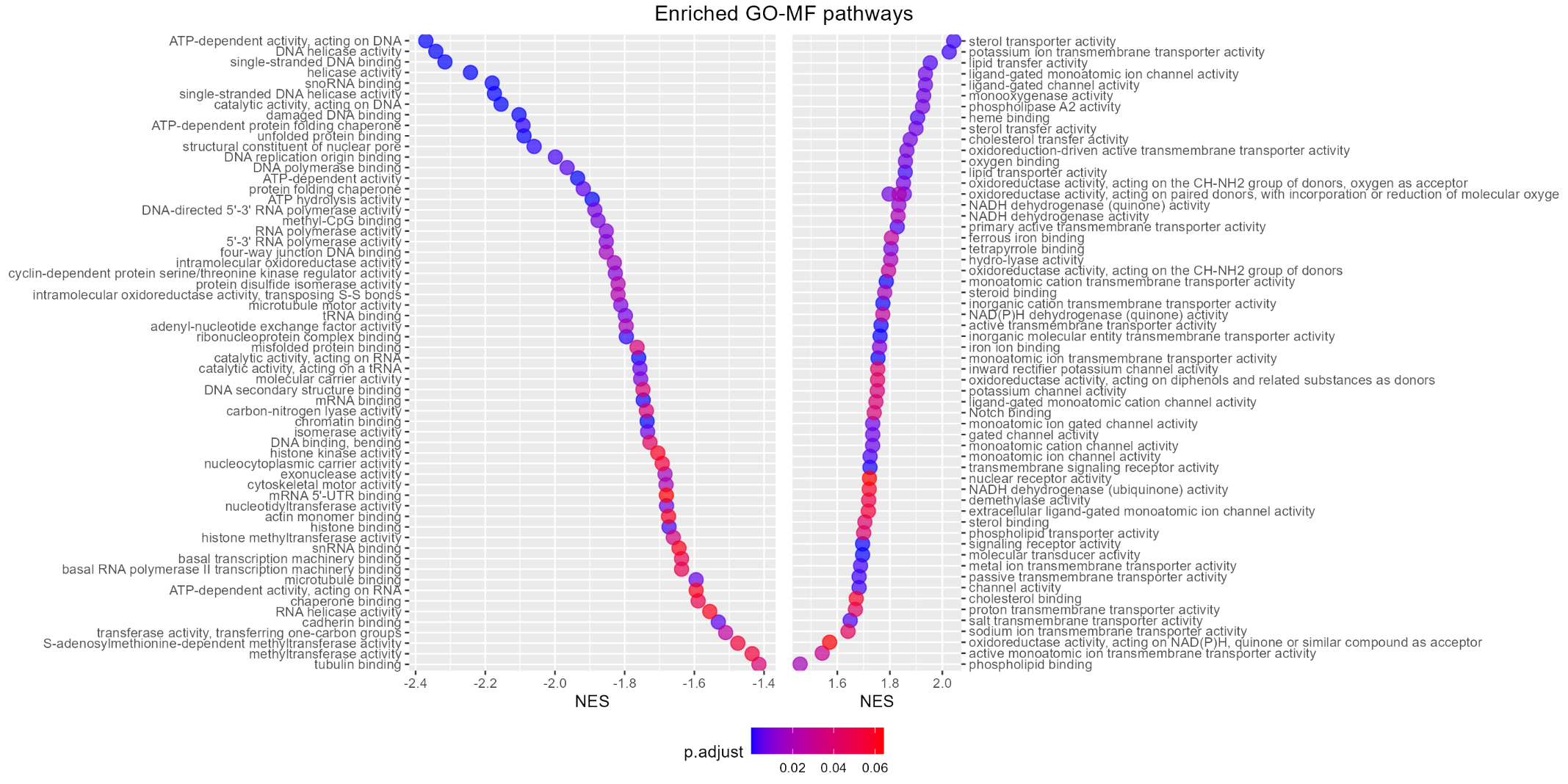
Figure S5
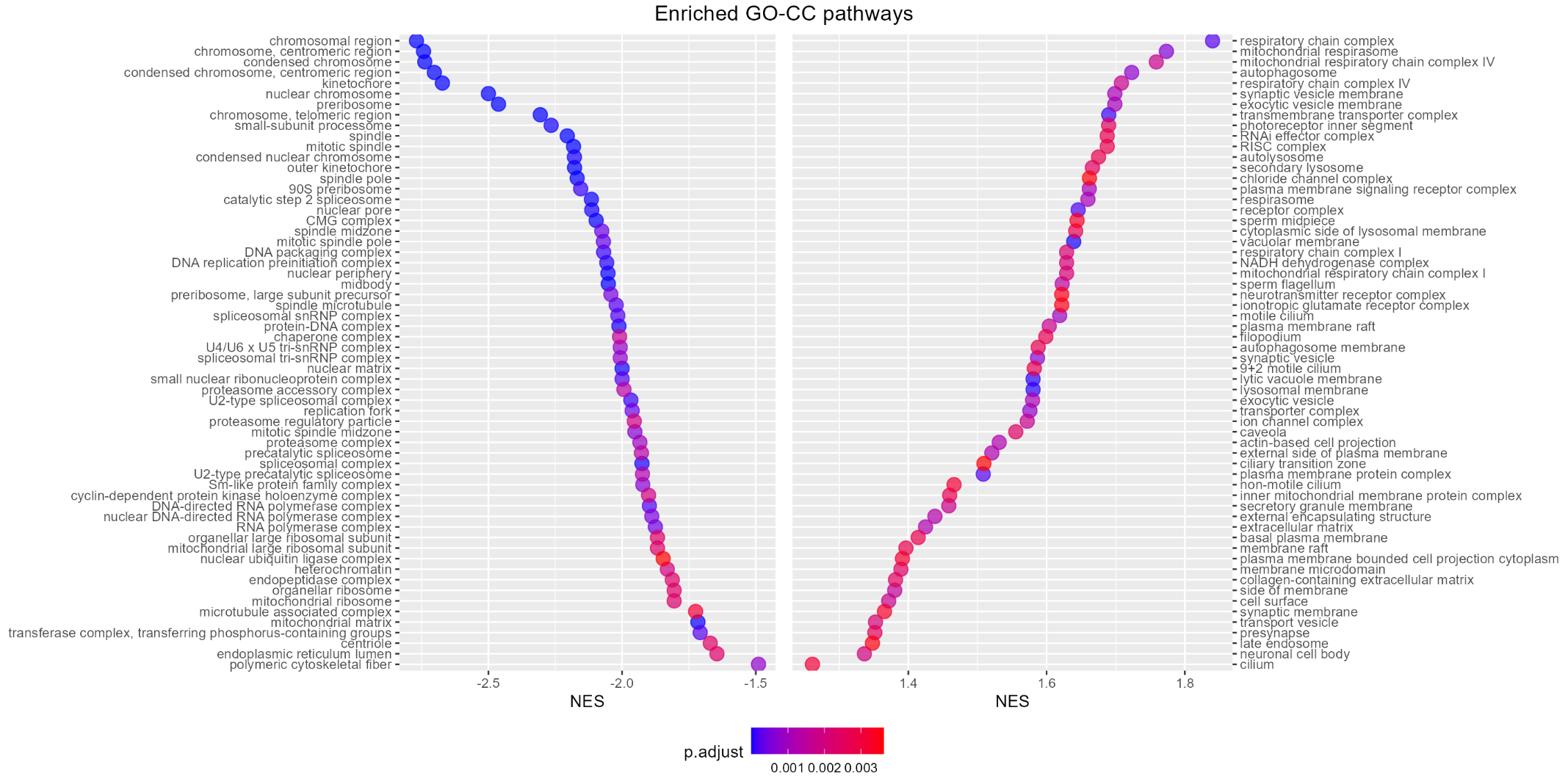
Figure S6
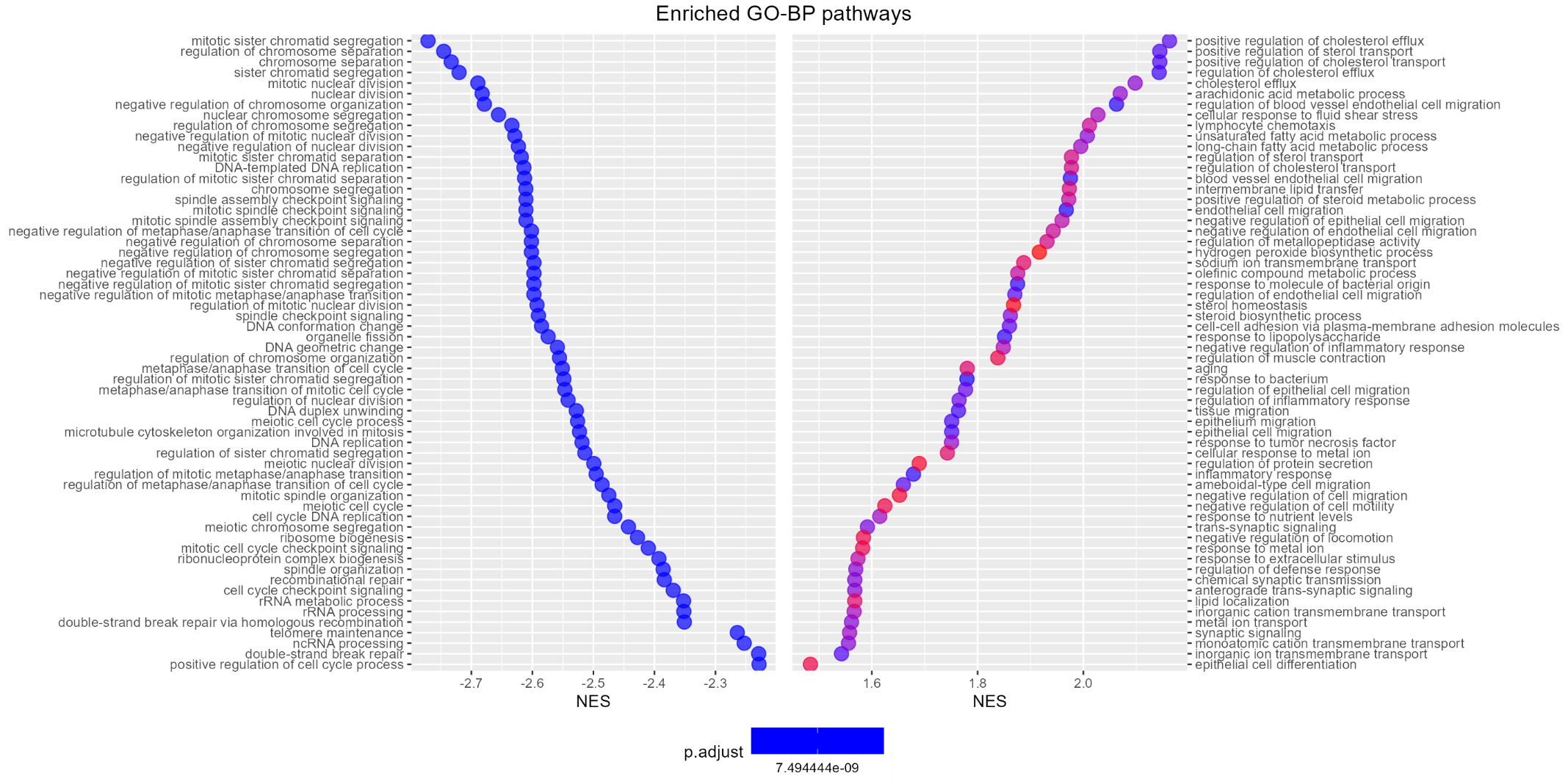
Figure S7
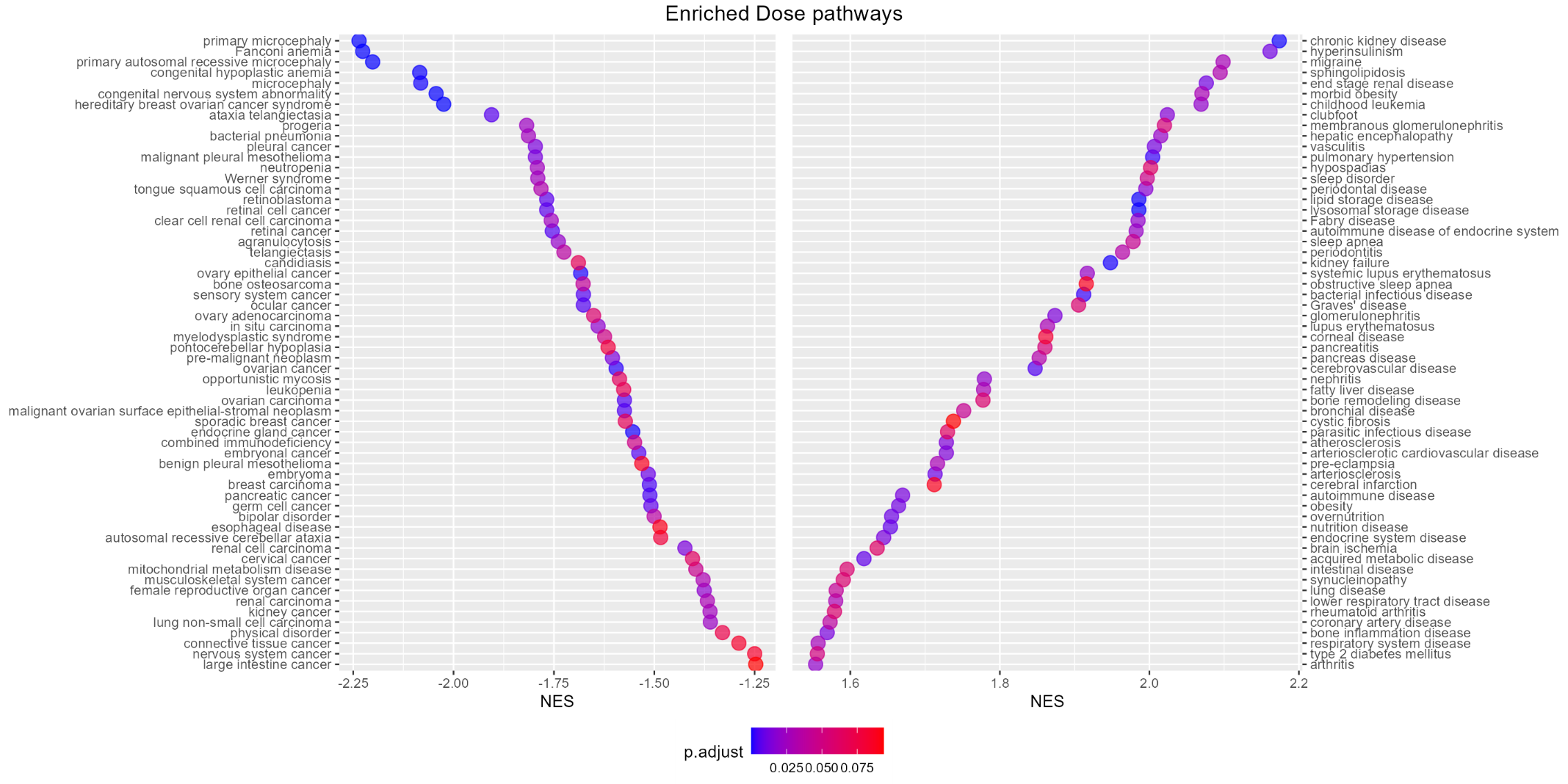
Figure S8

GSEA results on oexCells
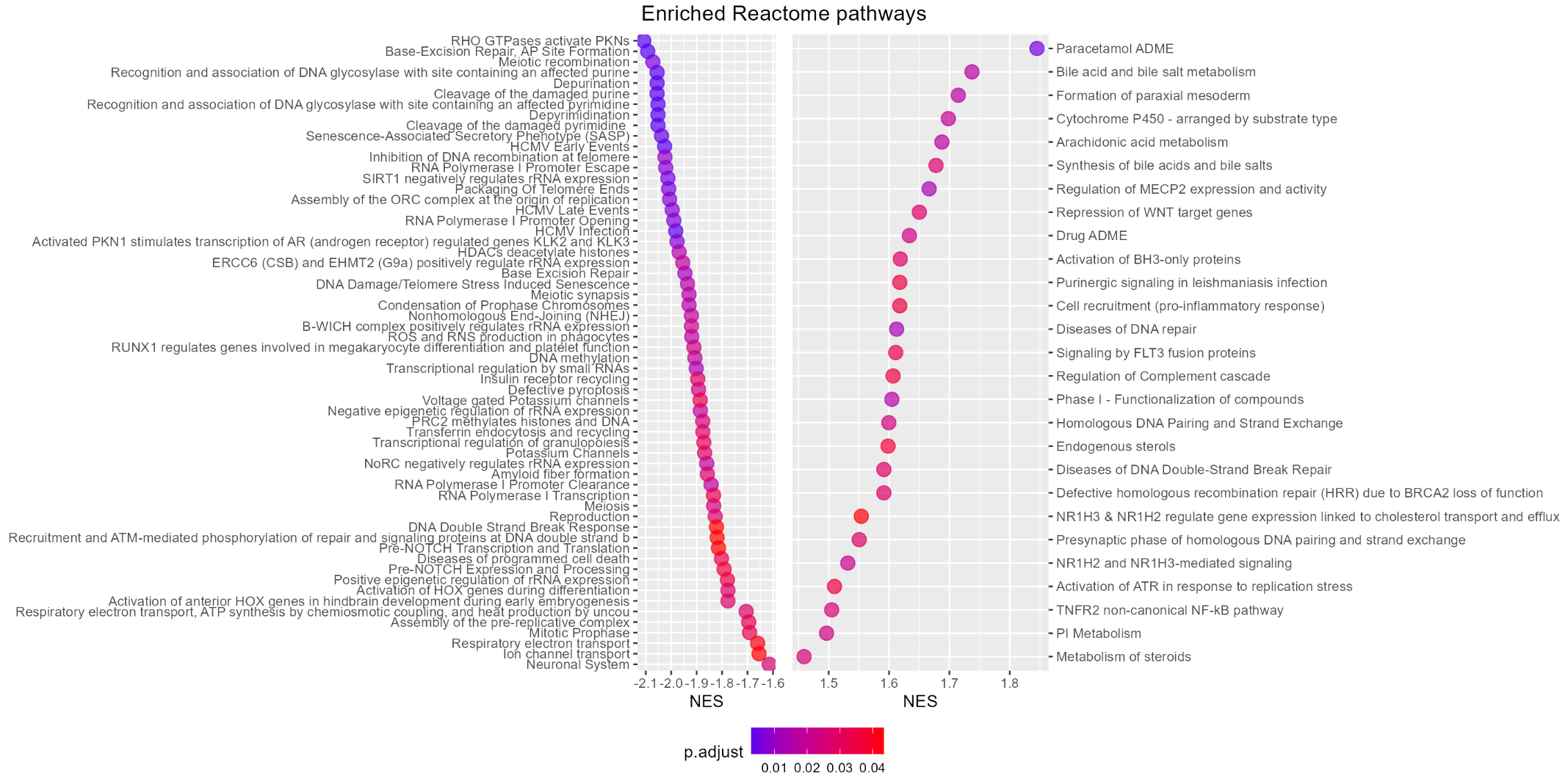
Figure S9
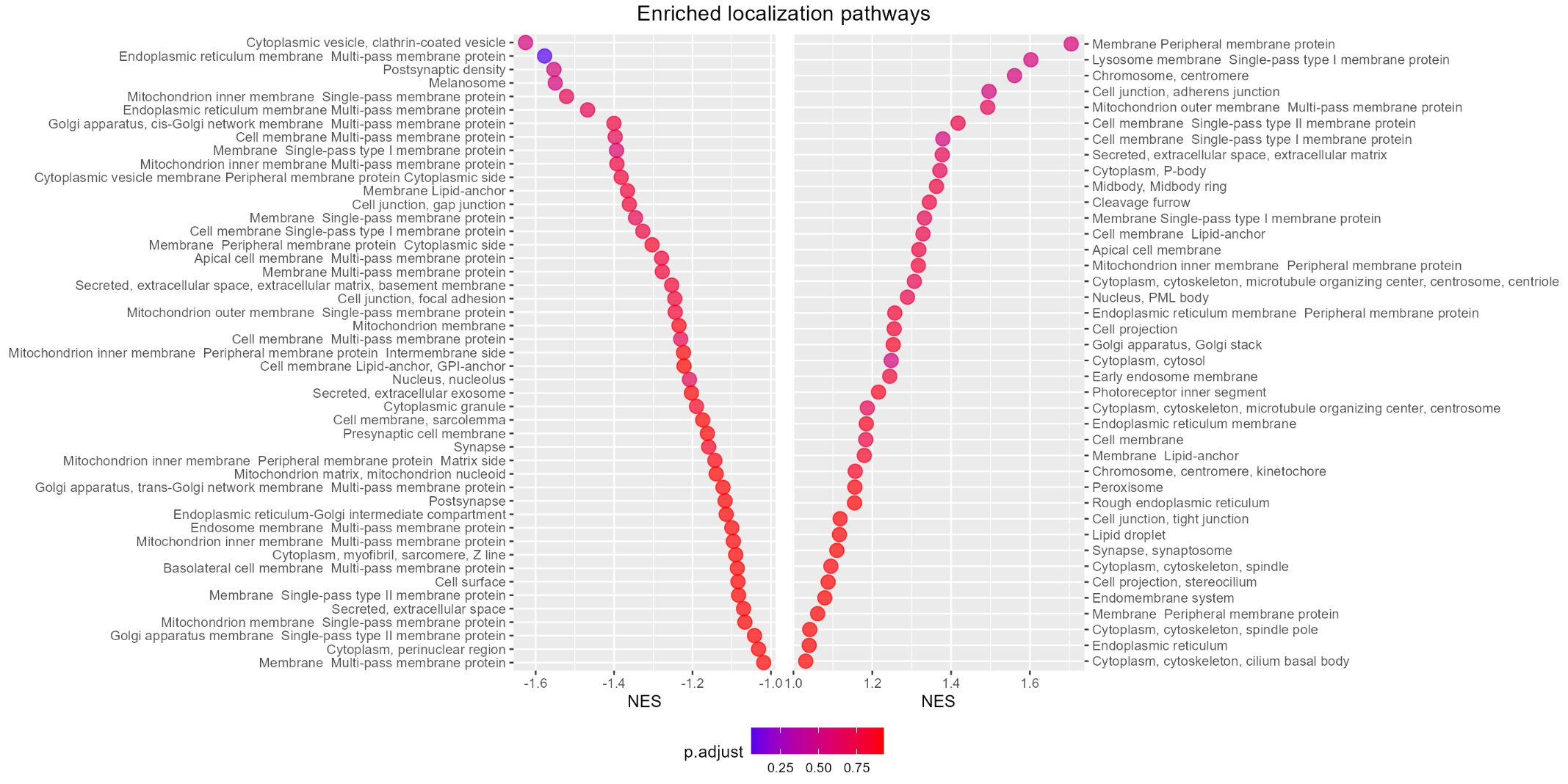
Figure S10
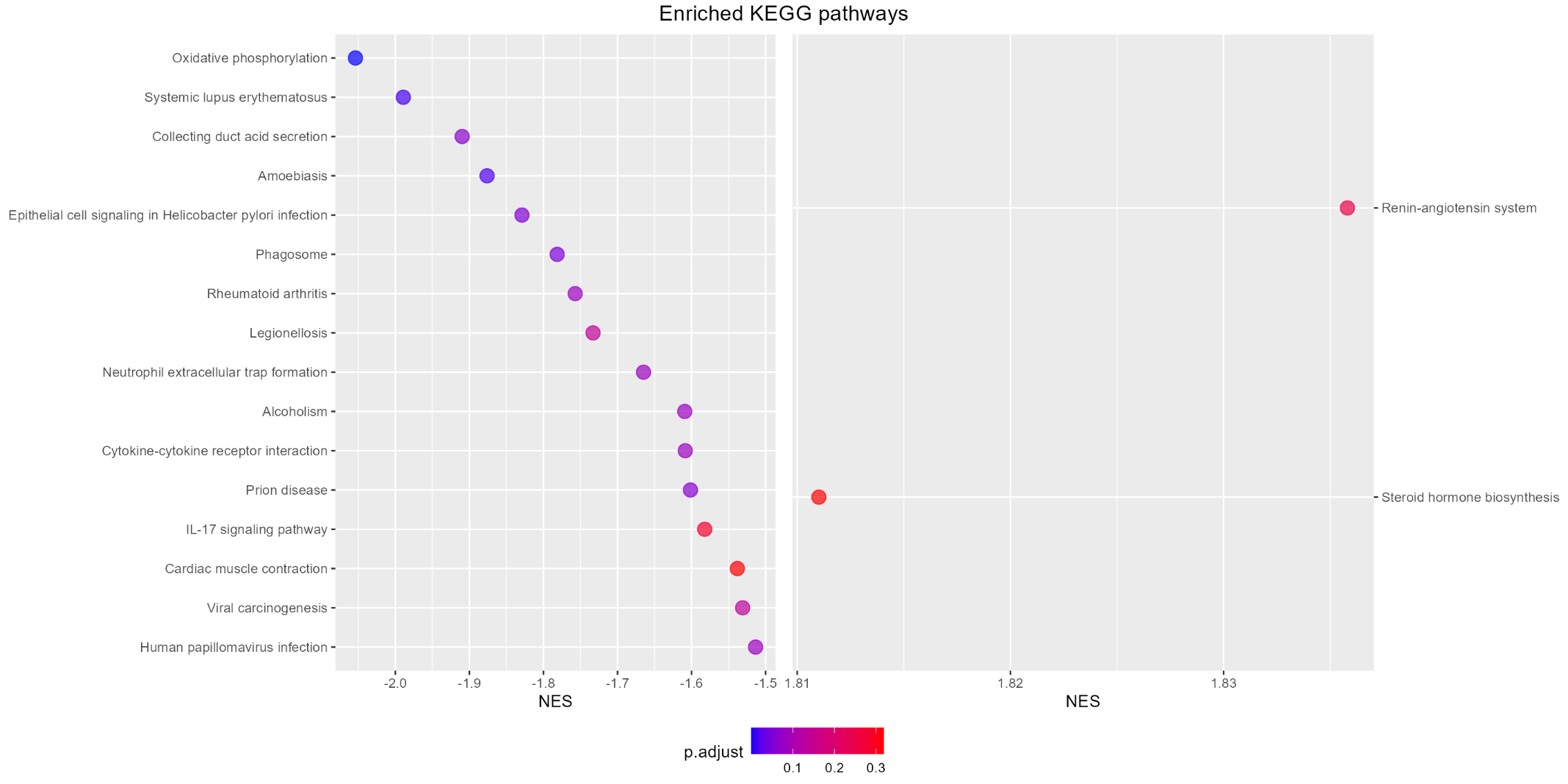
Figure S11
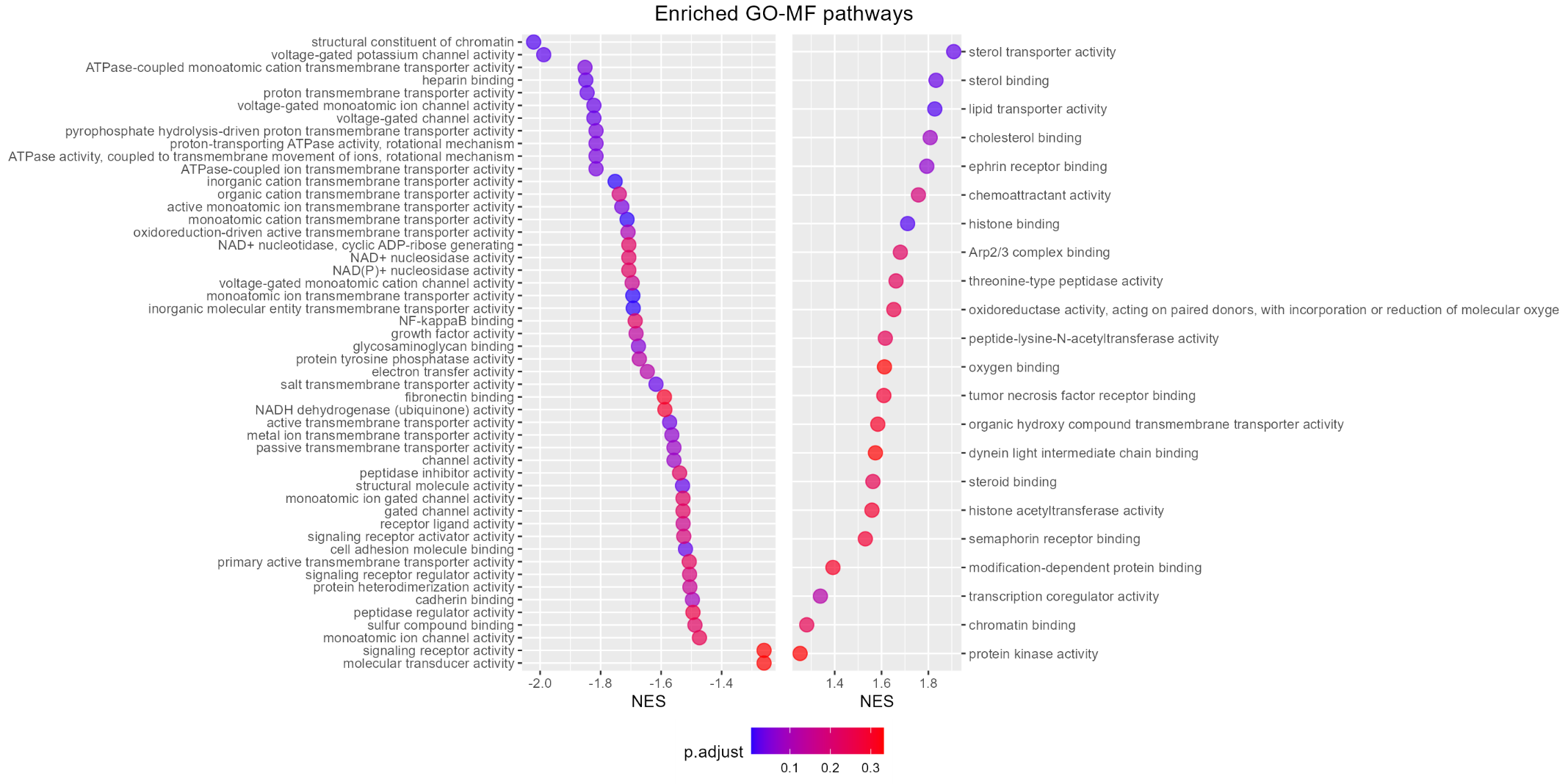
Figure S12
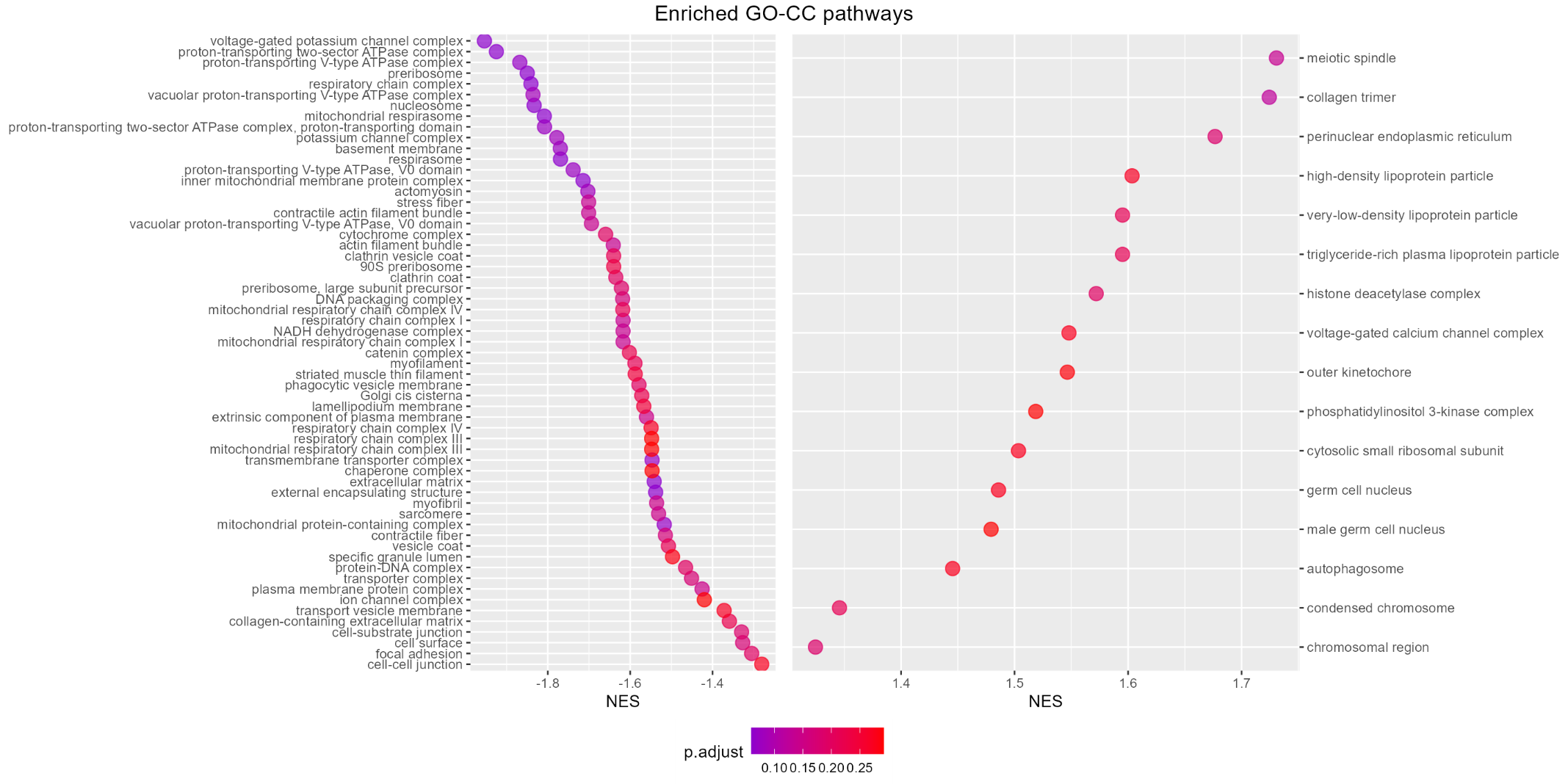
Figure S13
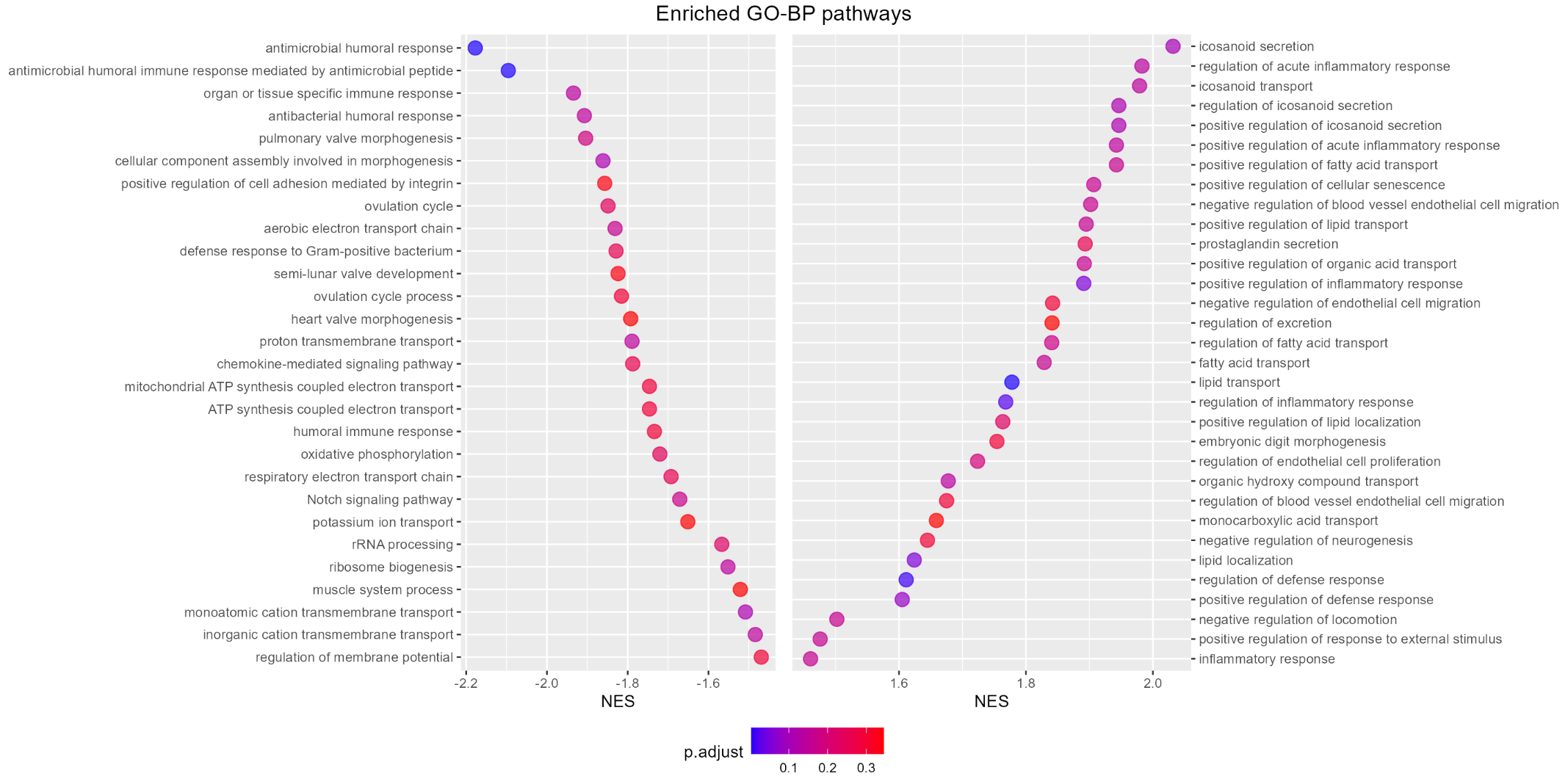
Figure S14
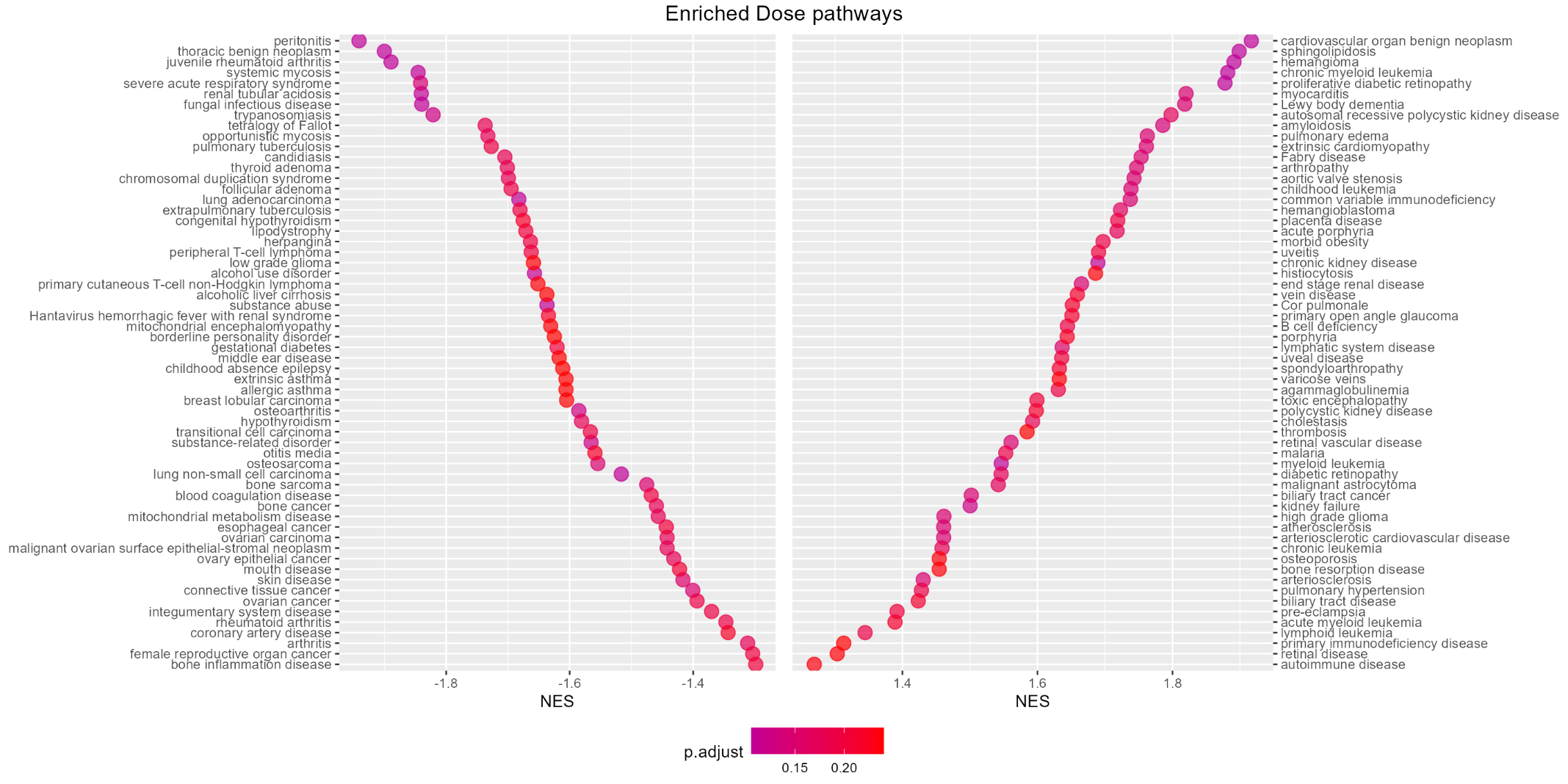
Figure S15
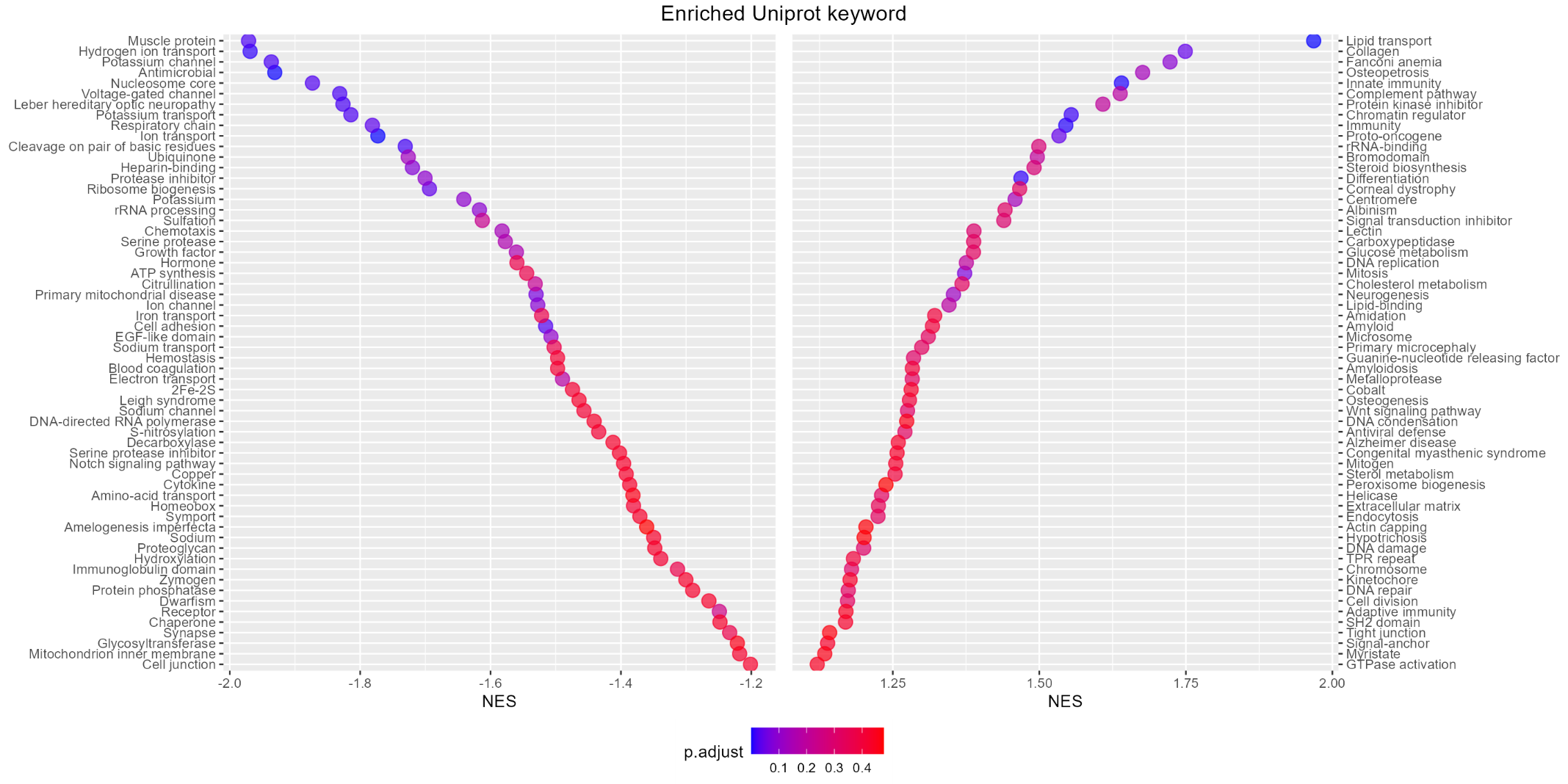
Figure S16
