# Supplementary material for: Functional omics of ORP7 in primary endothelial cells
Source: BMC Biol. 2024 Dec 18;22:292. doi: 10.1186/s12915-024-02087-6 (PMC11656939; doi:10.1186/s12915-024-02087-6)
Supplement: Supplementary file 3 — Additional file 3: Figs. S1–S26: Complete membrane images. [file 12915_2024_2087_MOESM3_ESM.docx]

**Additional File 3**


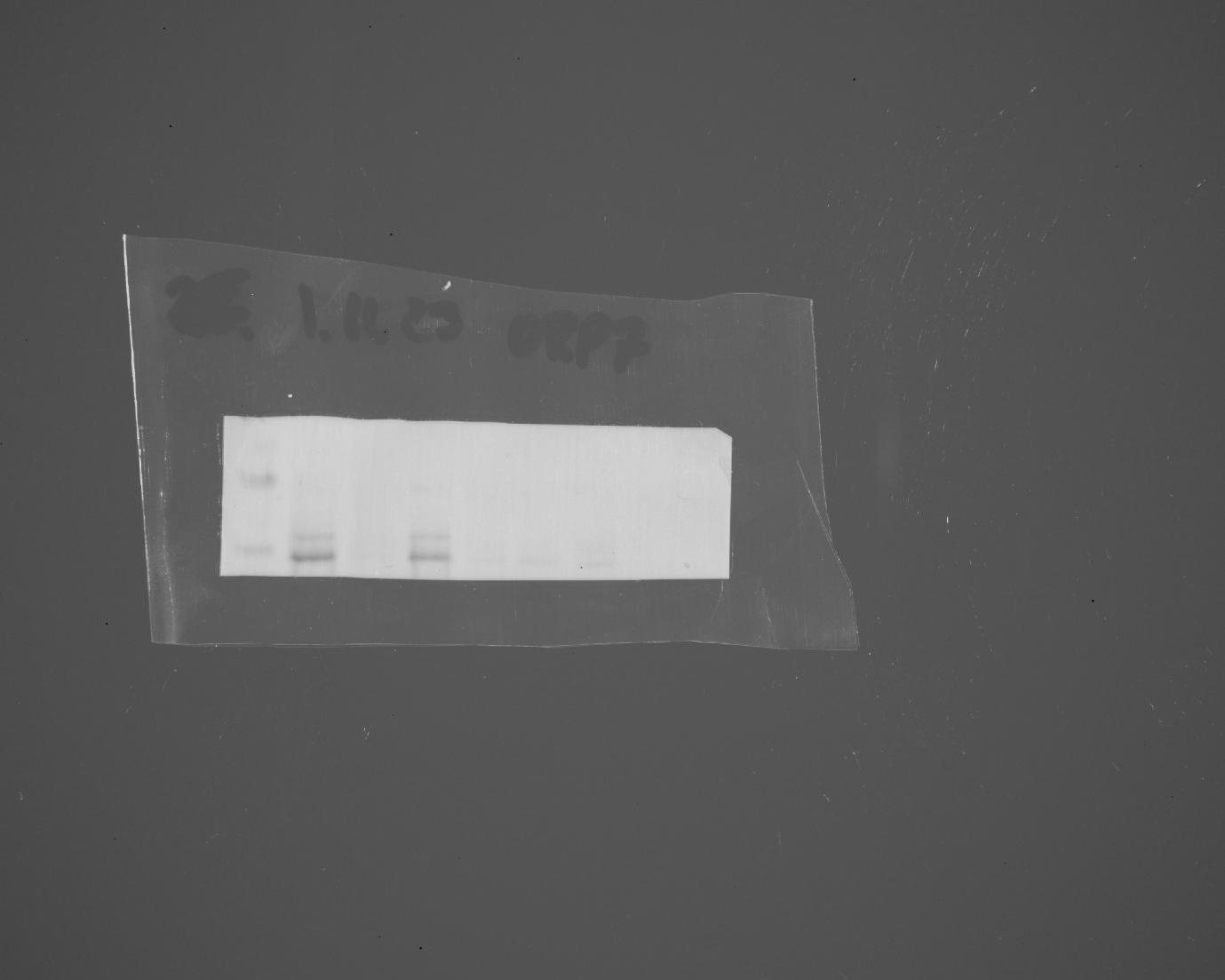


Figure S1. Full membrane images of ORP7 to make Figure 12


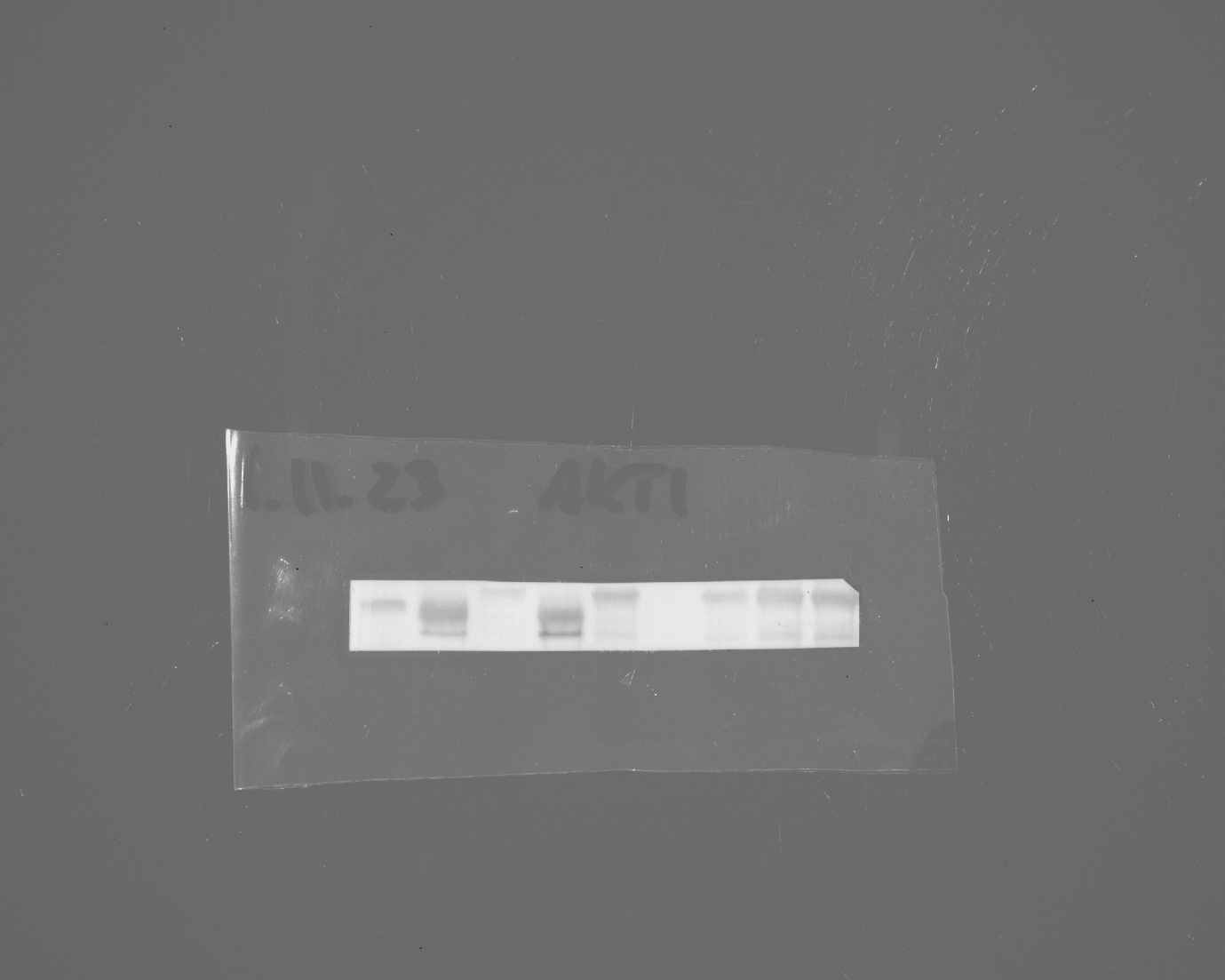


Figure S2. Full membrane image of AKT1 used to make Figure 12


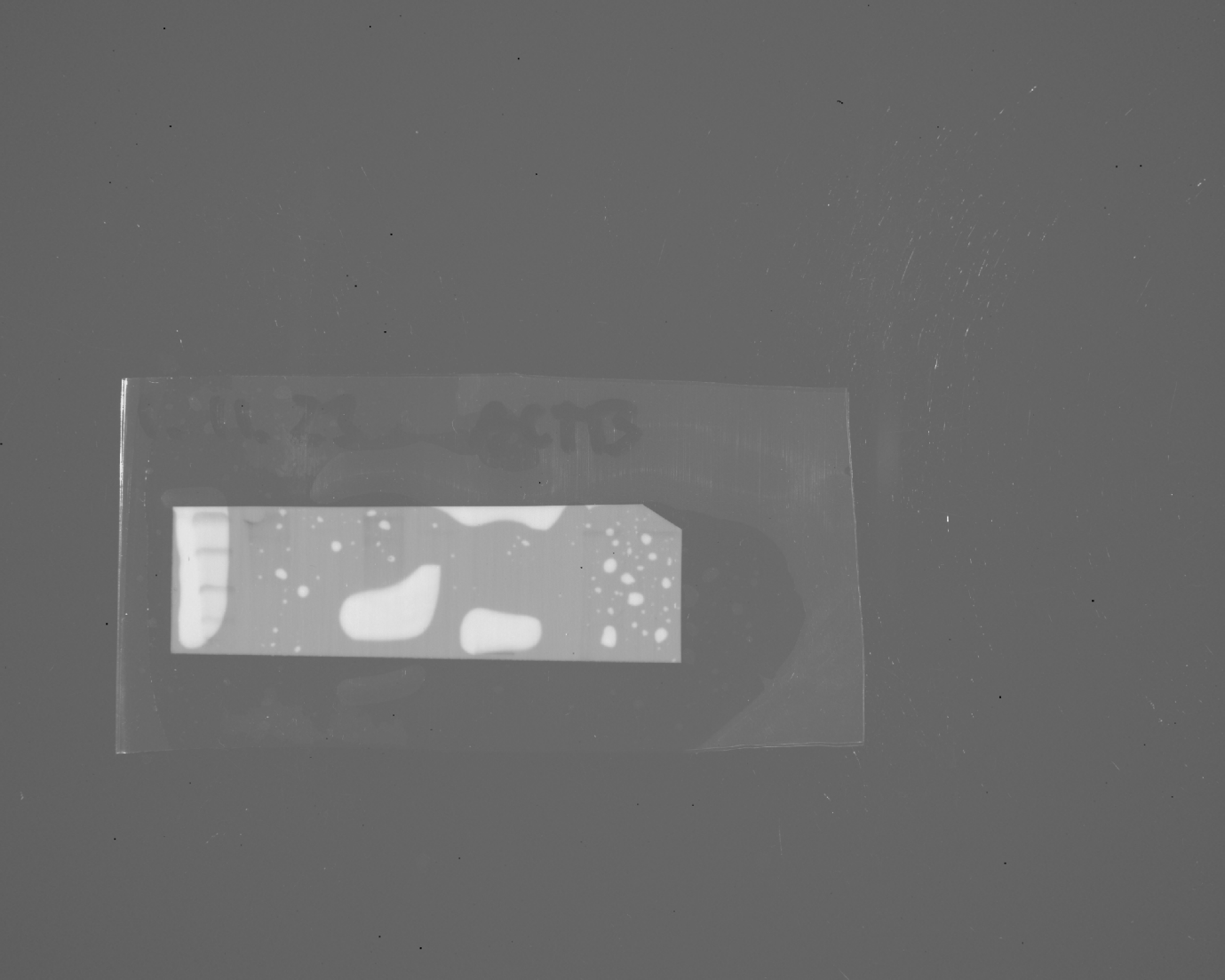


Figure S3. Full membrane images used to make Figure 12


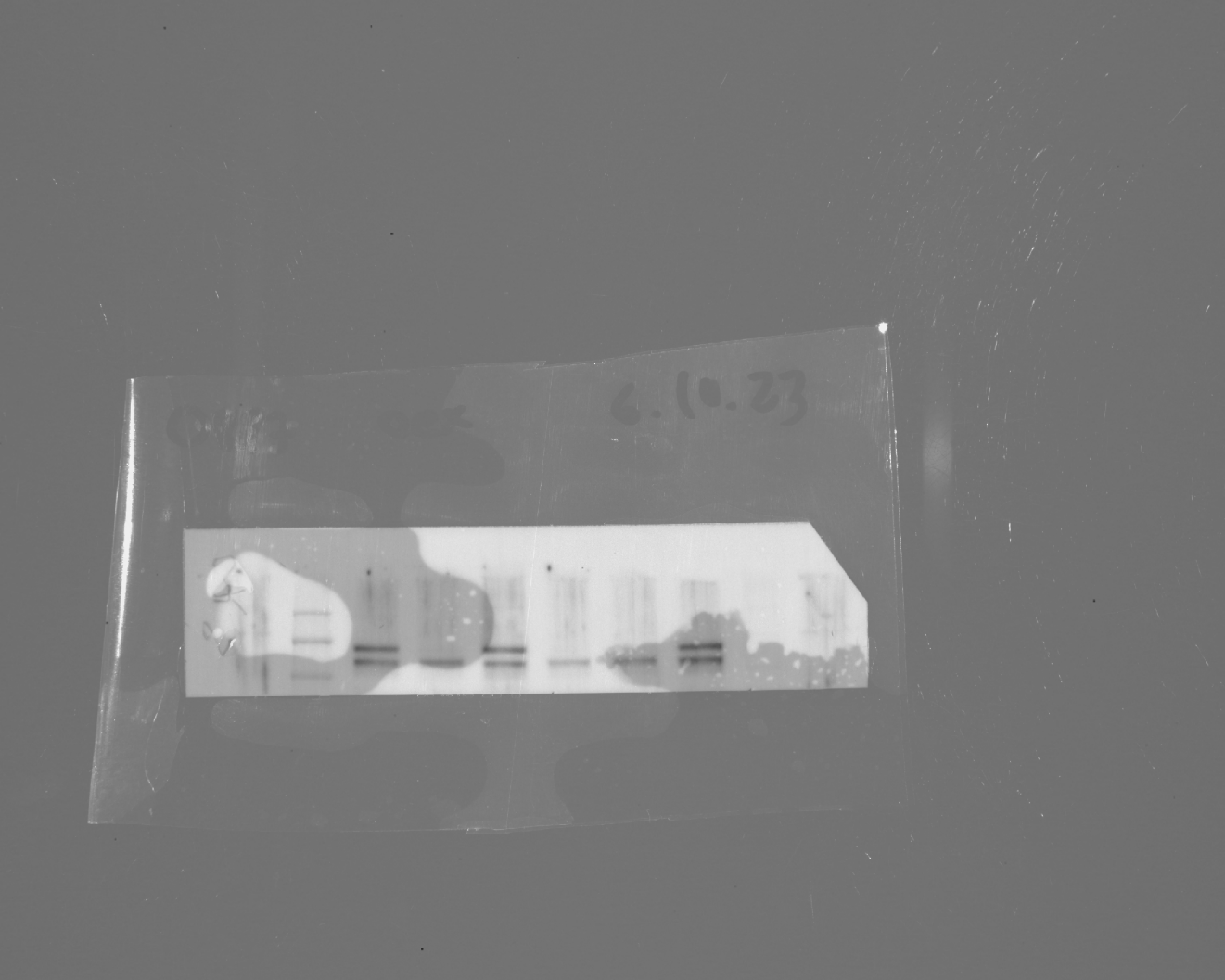


Figure S4. Full membrane image of ORP7 in oex samples used to make Figure S11B


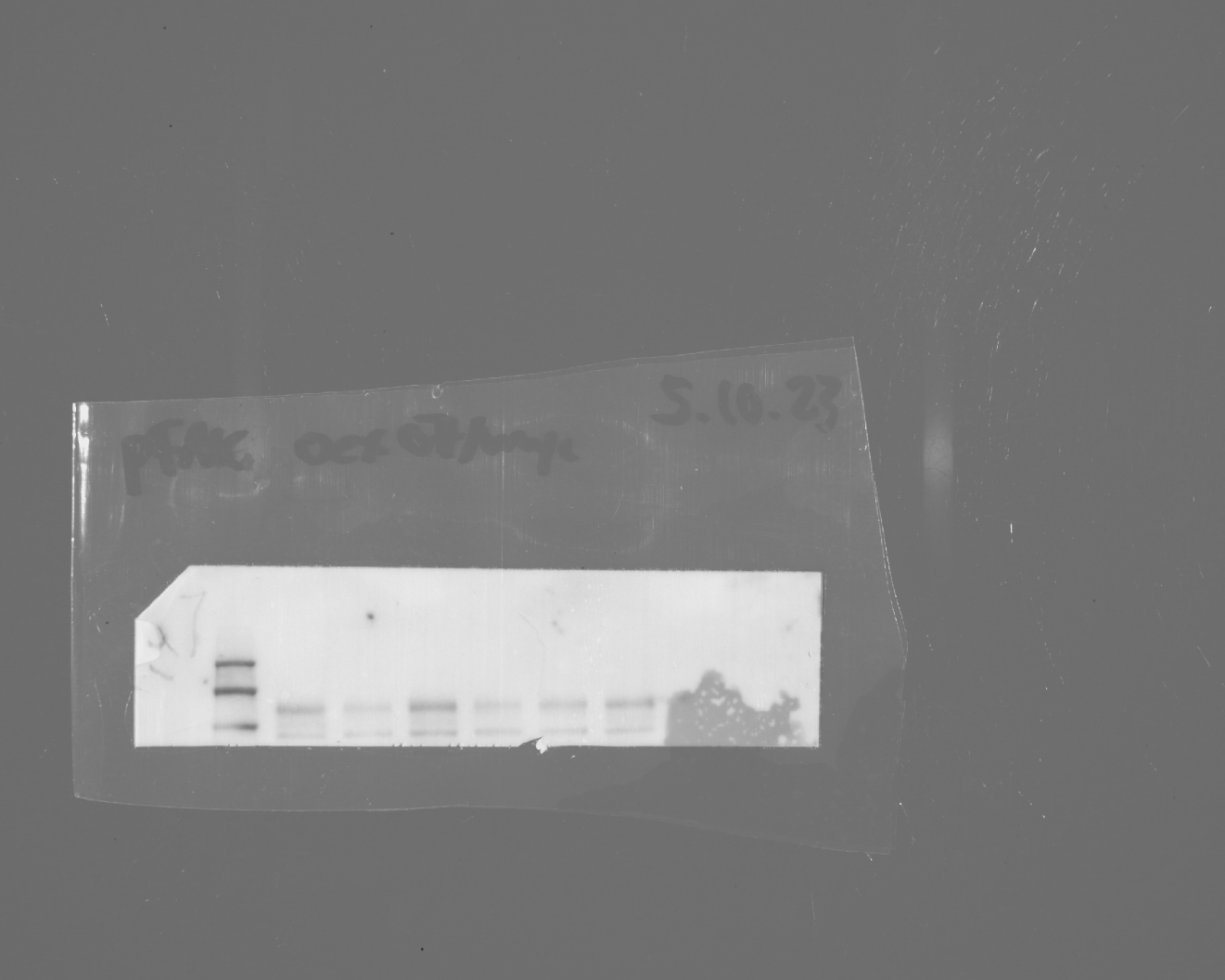


Figure S5. Full membrane image of pFAK in oex samples used to make Figure S11B


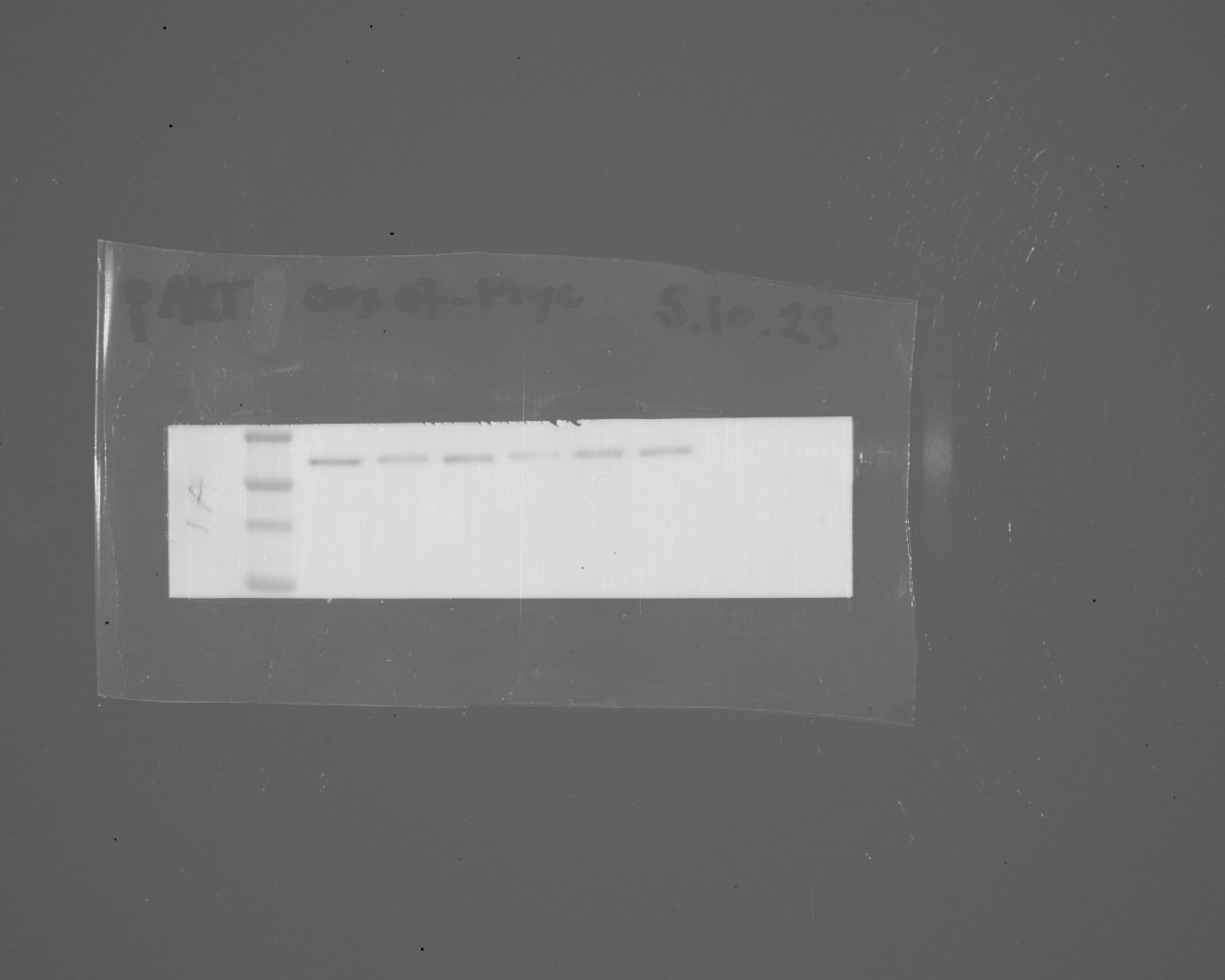


Figure S6. Full membrane image of pAKT in oex samples used to make Figure S11B


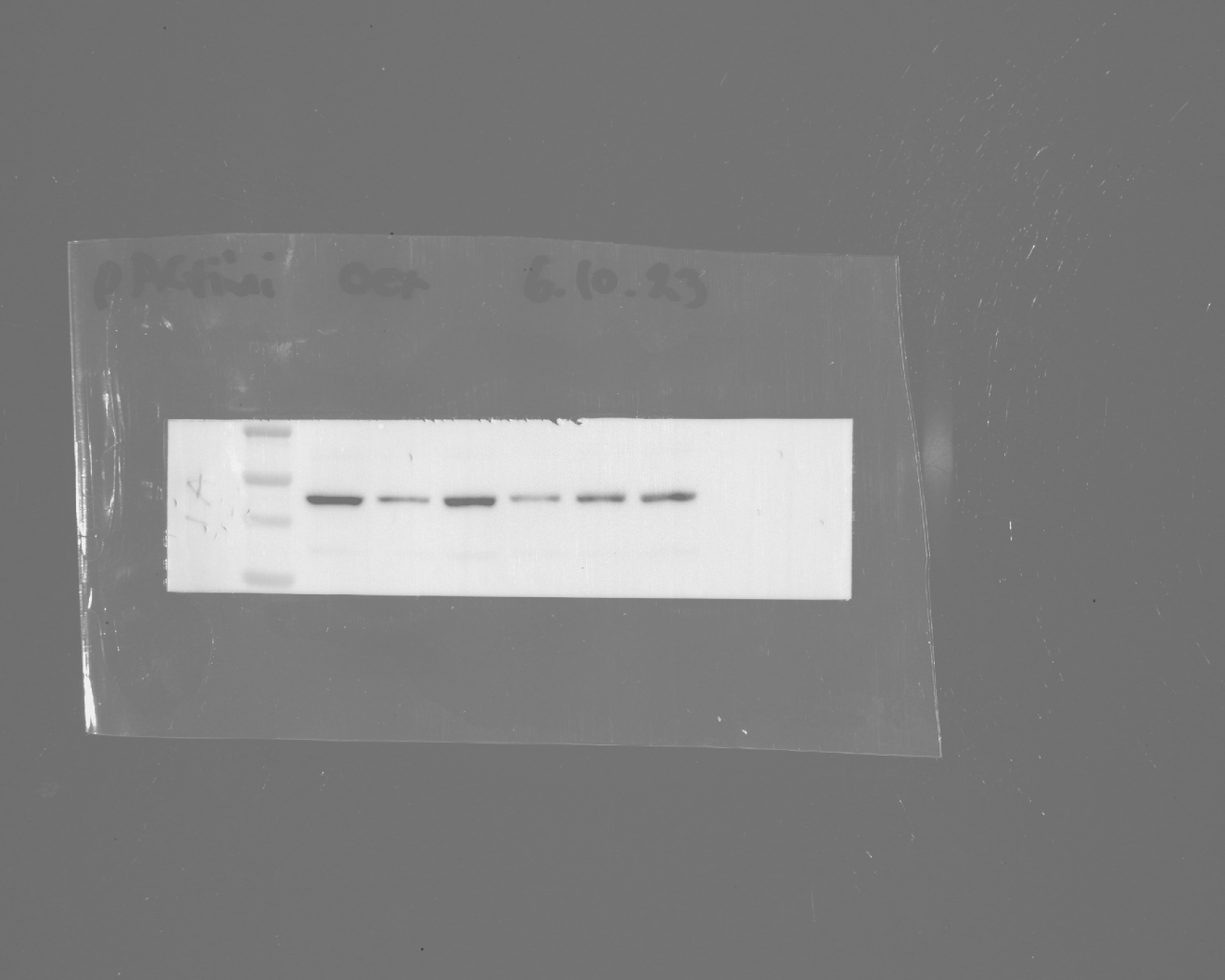


Figure S7. Full membrane image of ACTB in phosphorylate proteins from oex samples used to make Figure S11B


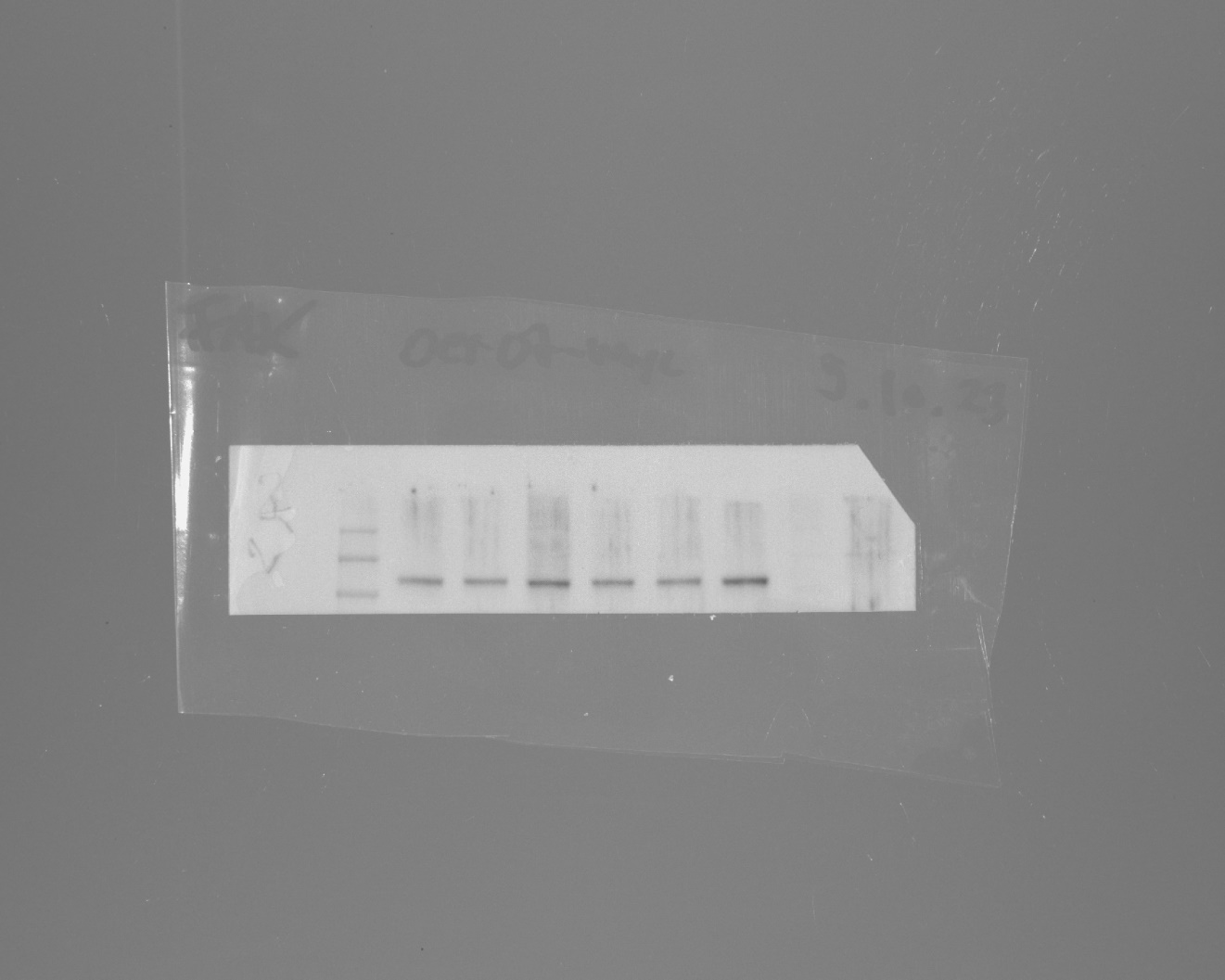


Figure S8. Full membrane image of FAK in oex samples used to make Figure S11B


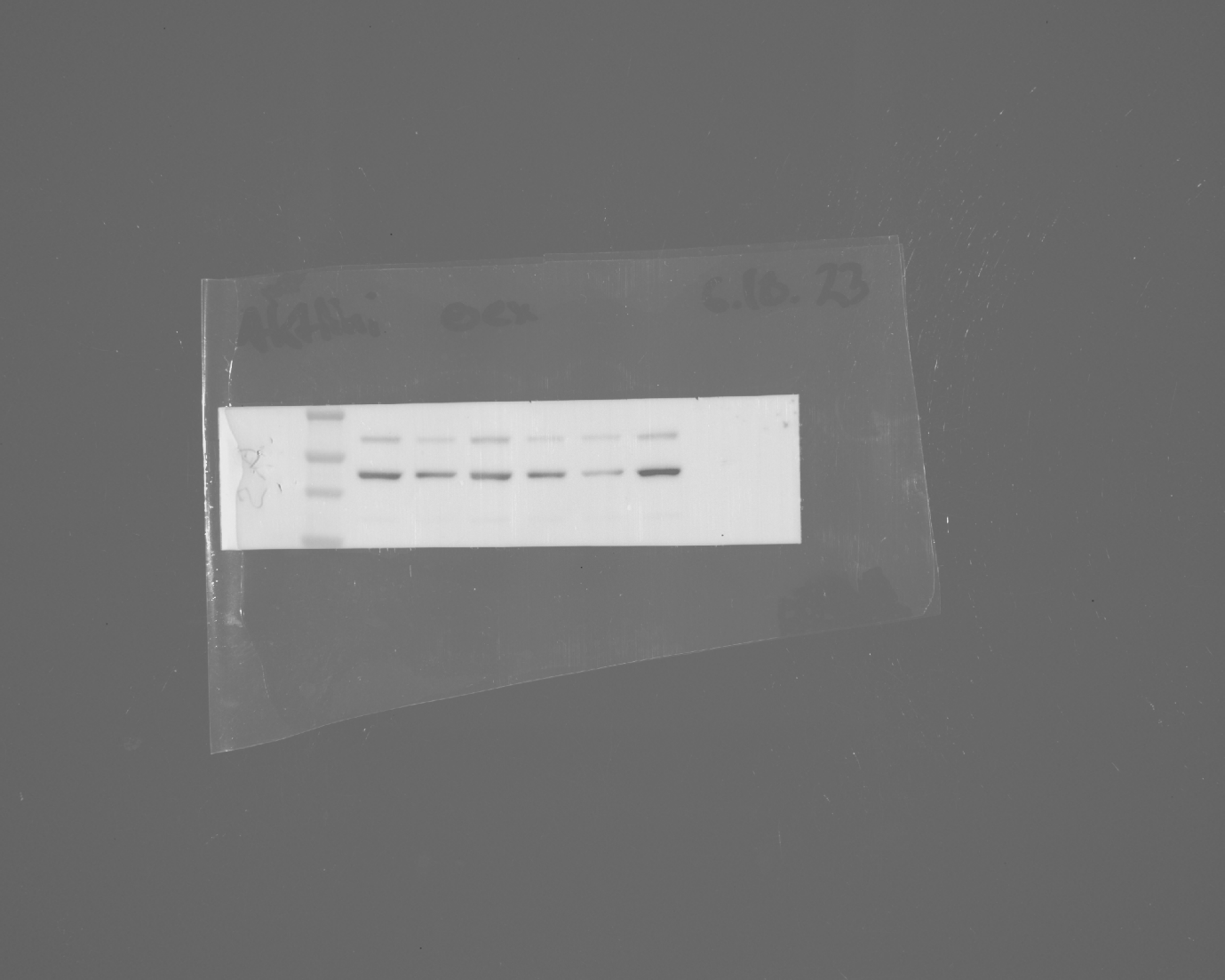


Figure S9. Full membrane image of ACTB in oex samples used to make Figure S11B


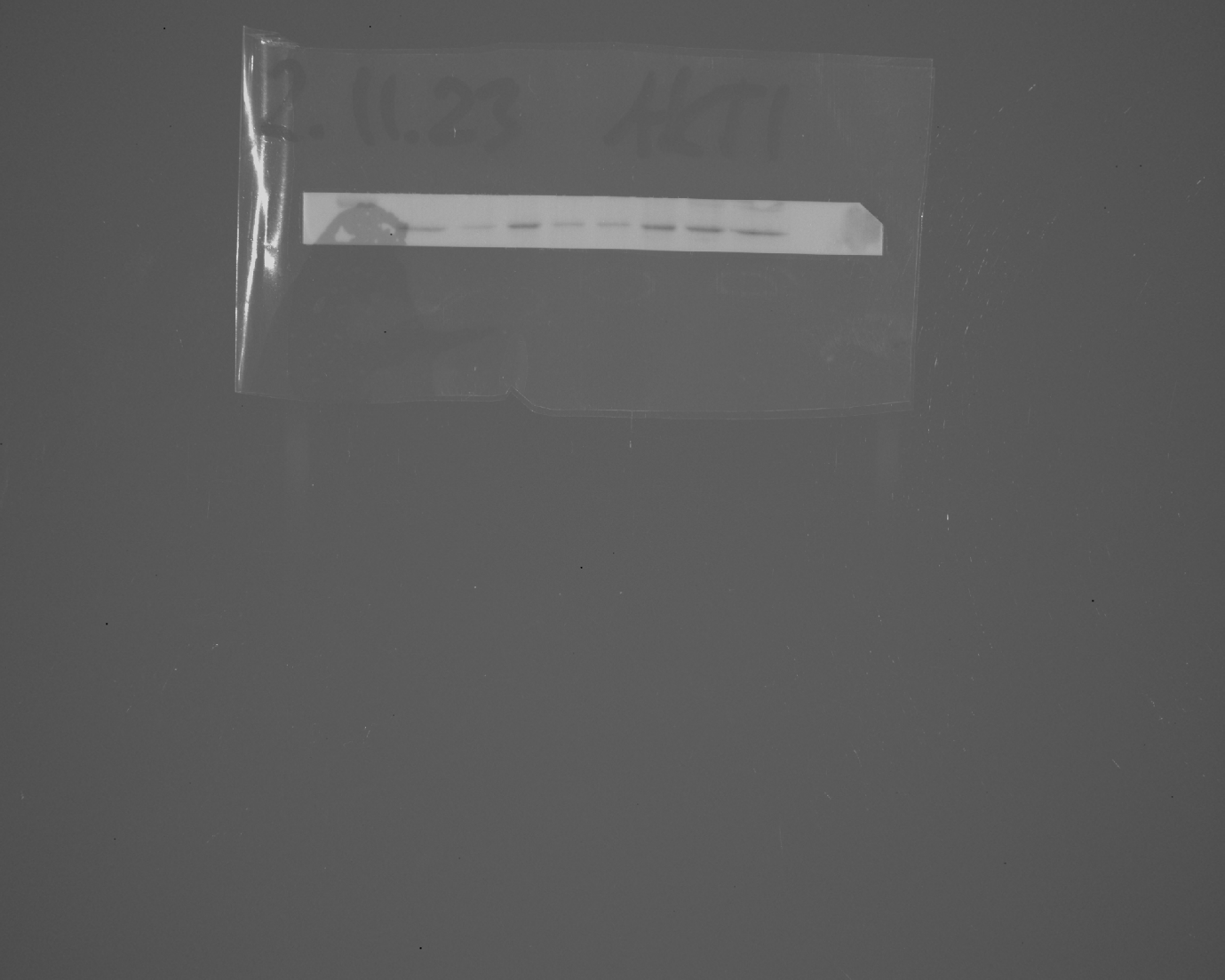


Figure S10. Full membrane image of AKT in oex samples used to make Figure S11B


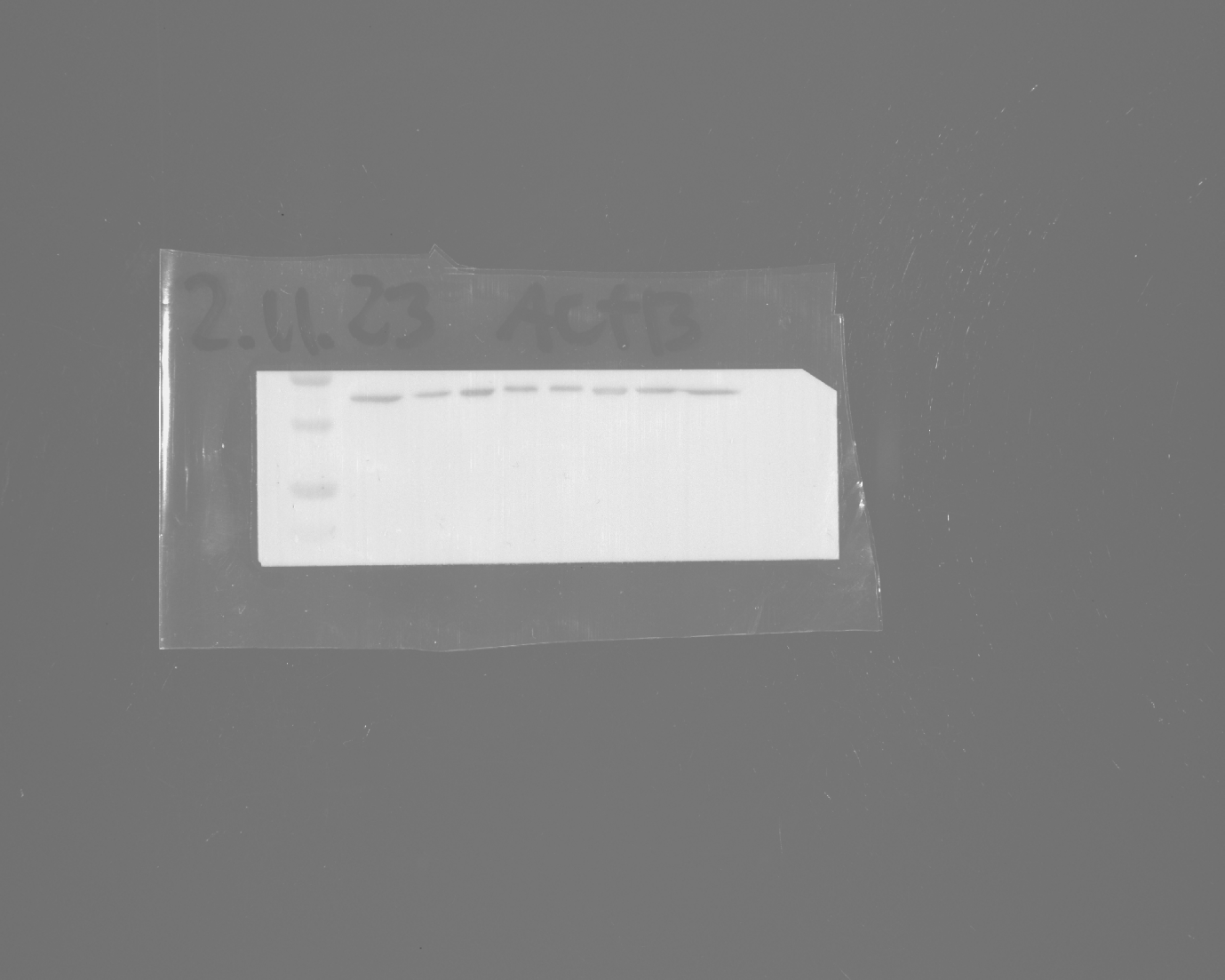


Figure S11. Full membrane image of ACTB in oex samples used to make Figure S11B


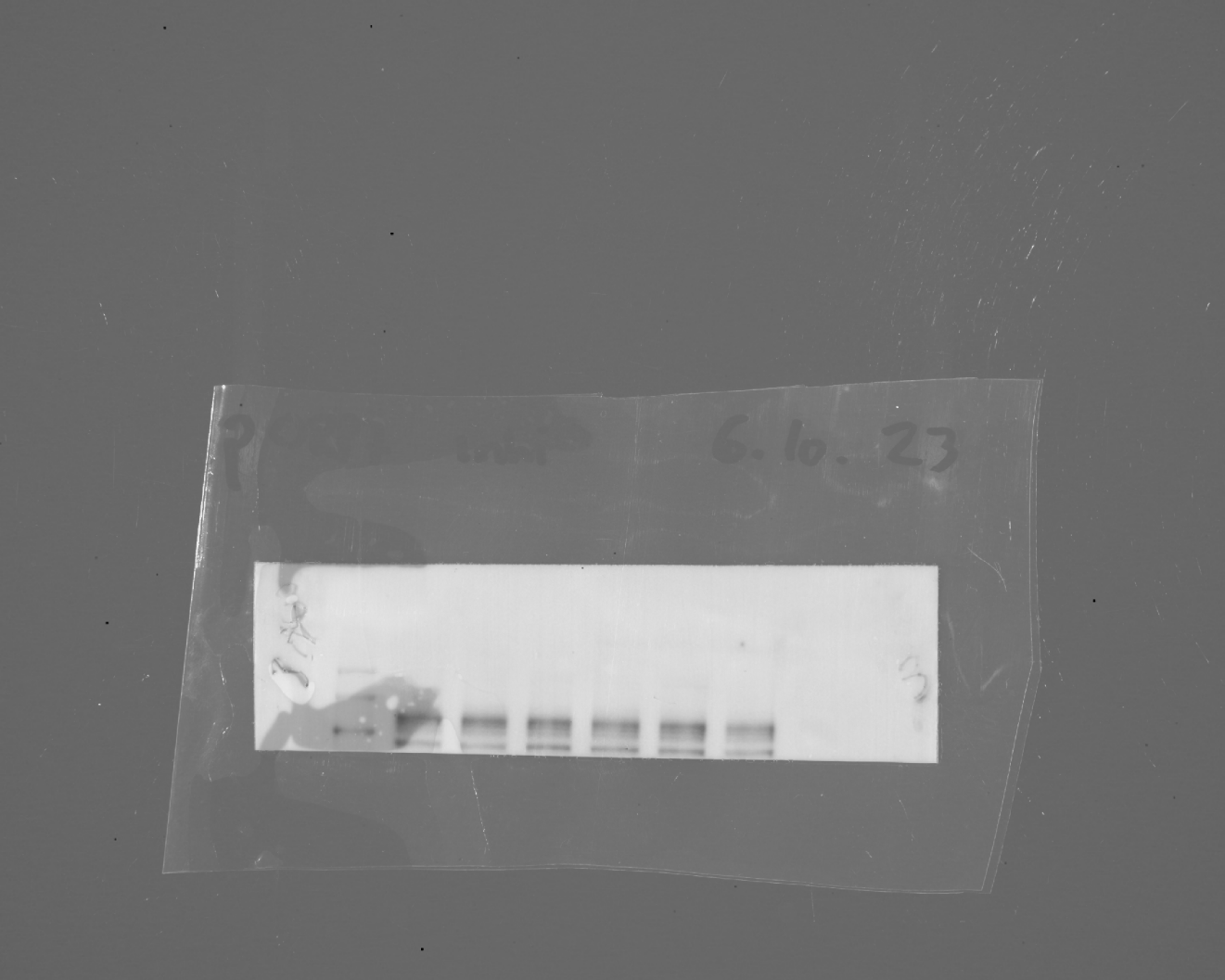


Figure S12. Full membrane image of ORP7 in treated samples used to make Figure S11B


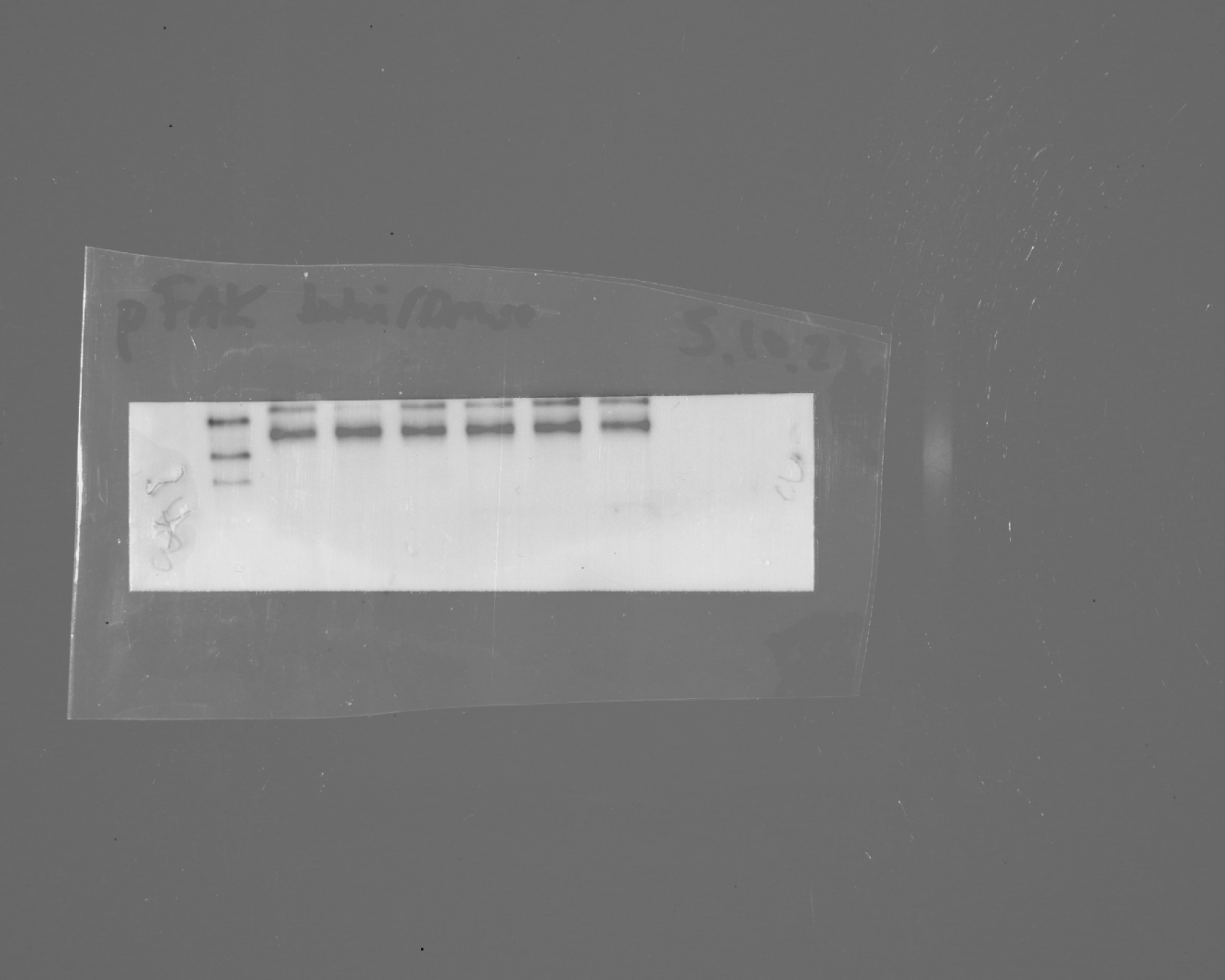


Figure S13. Full membrane image of pFAK in treated samples used to make Figure S11B


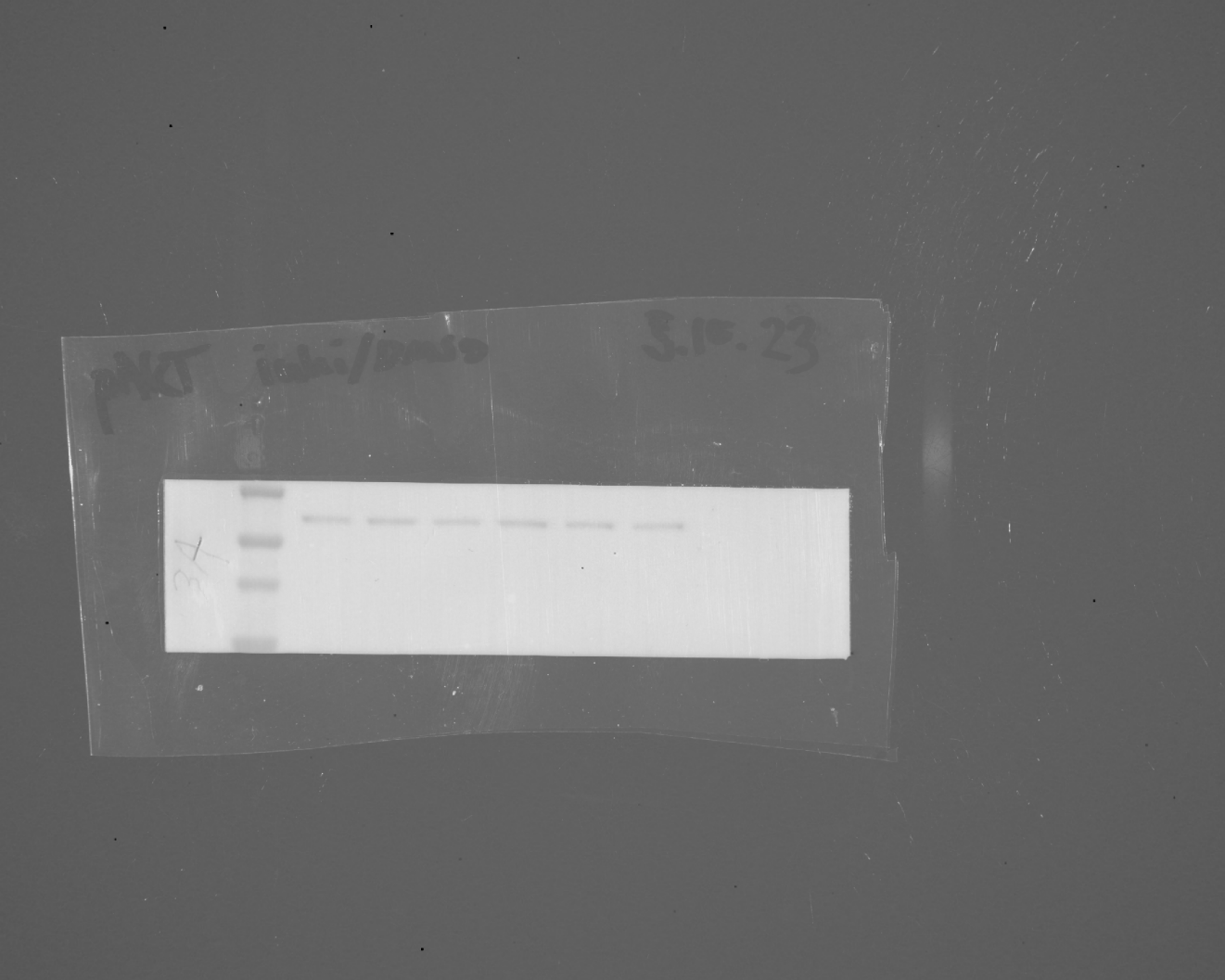


Figure S14. Full membrane image of pAKT1 in treated samples used to make Figure S11B


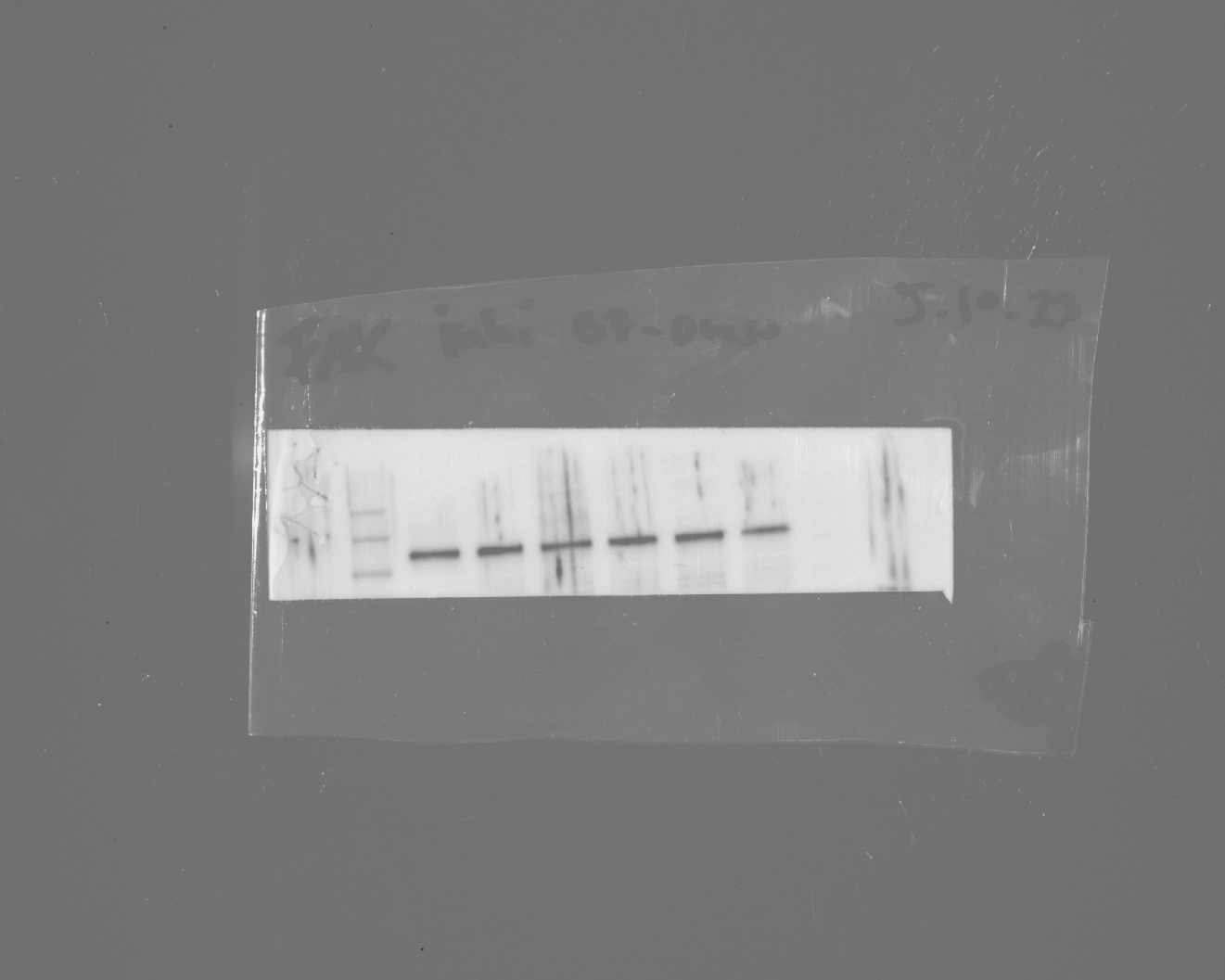


Figure S15. Full membrane image of FAK in treated samples used to make Figure S11B


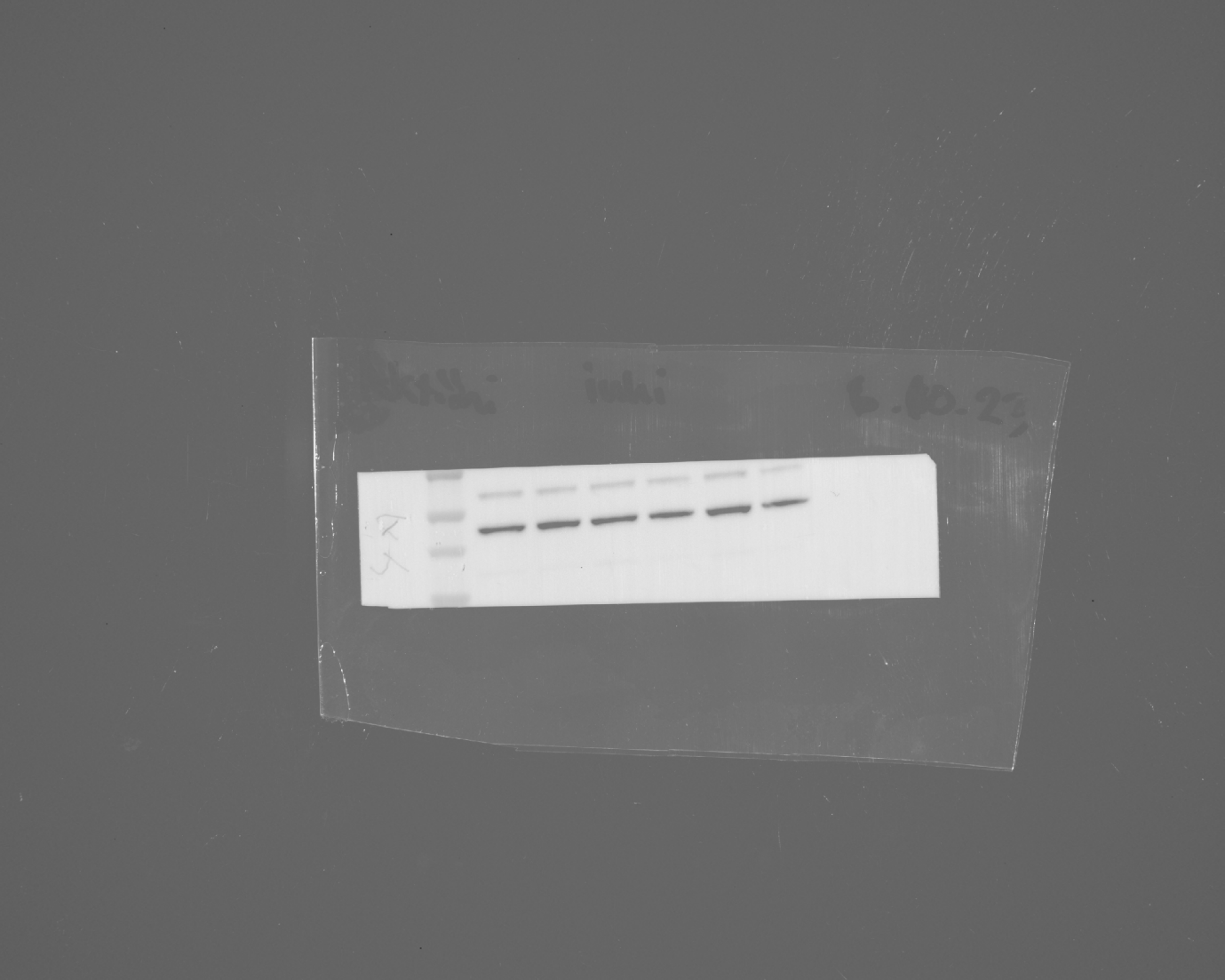


Figure S16. Full membrane image of ACTB in treated samples used to make Figure S11B


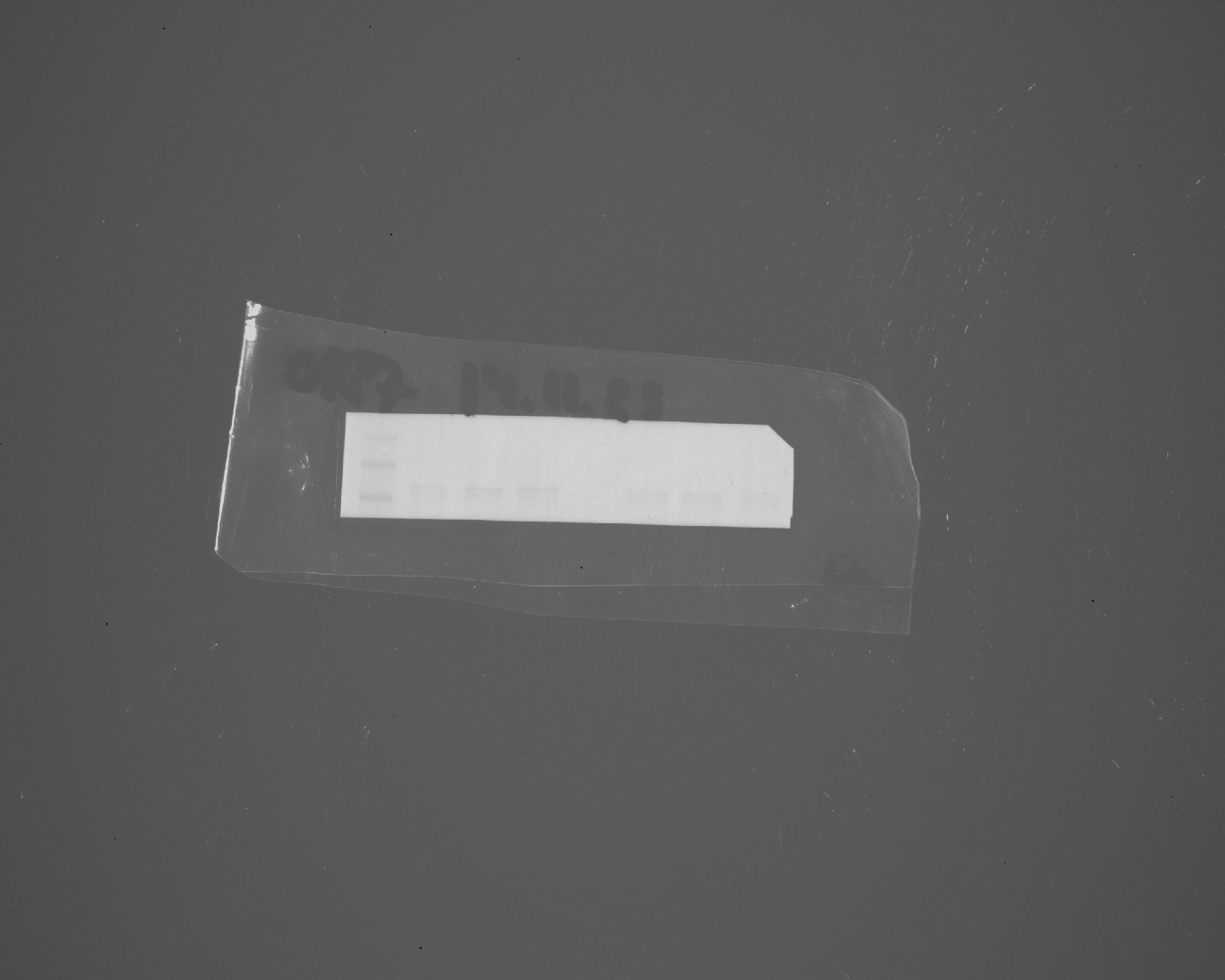


Figure S17. Full membrane image of ORP7 in treated samples used to make Figure S11B


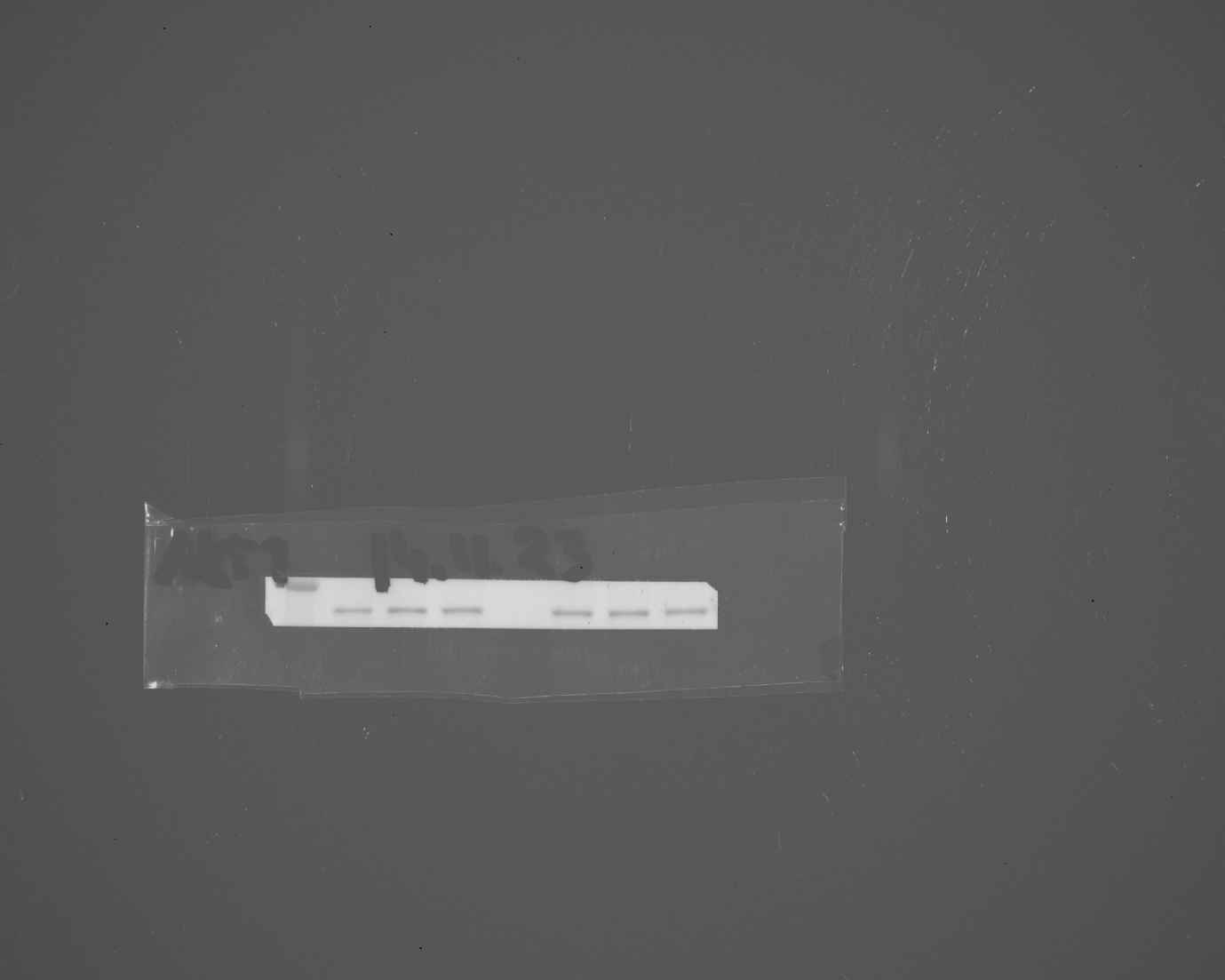


Figure S18. Full membrane image of AKT1 in treated samples used to make Figure S11B


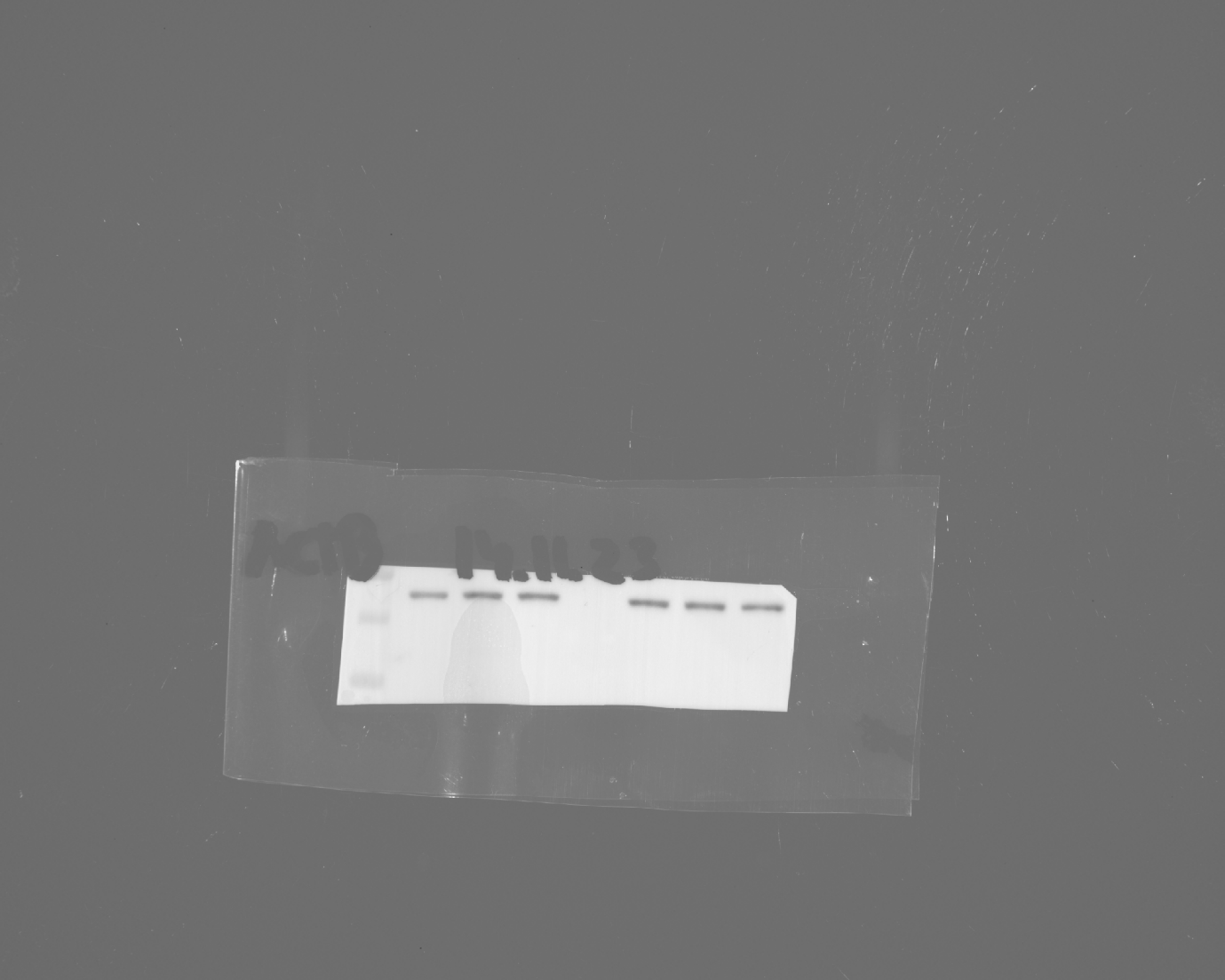


Figure S19. Full membrane image of ACTB in treated samples used to make Figure S11B


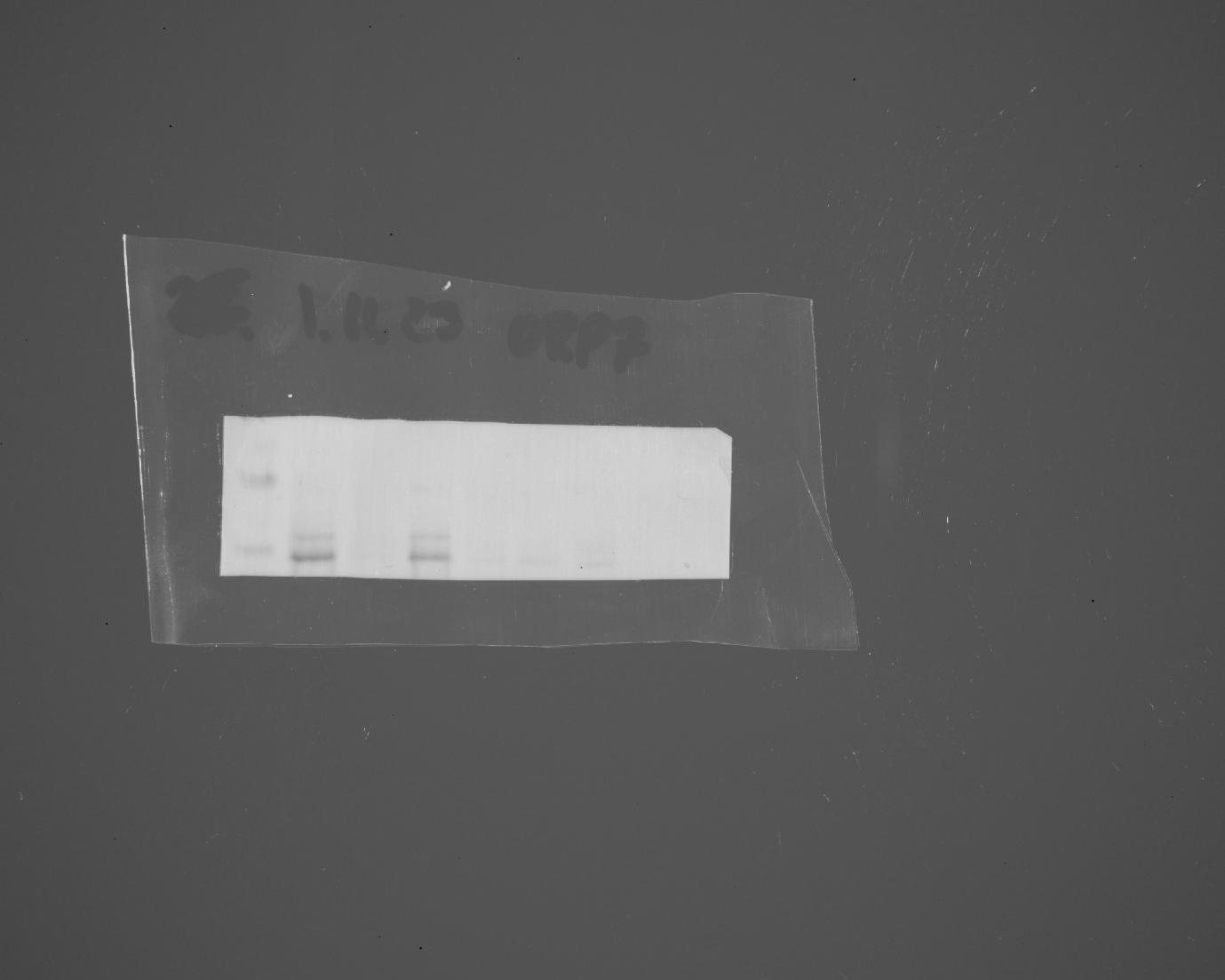


Figure S20. Full membrane image of ORP7 in oex samples used to make Figure 12


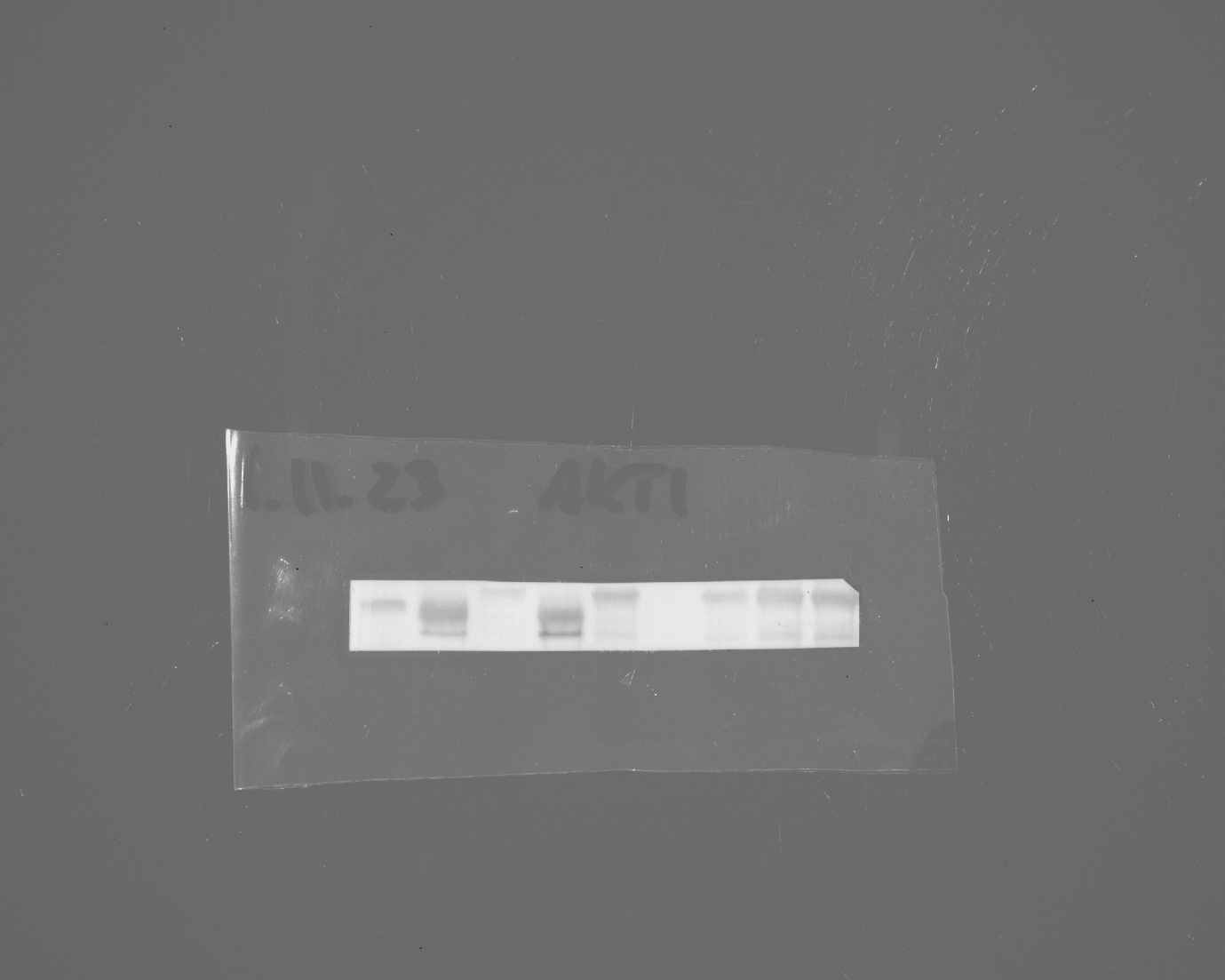


Figure S21. Full membrane image of AKT1 in oex samples used to make Figure 12


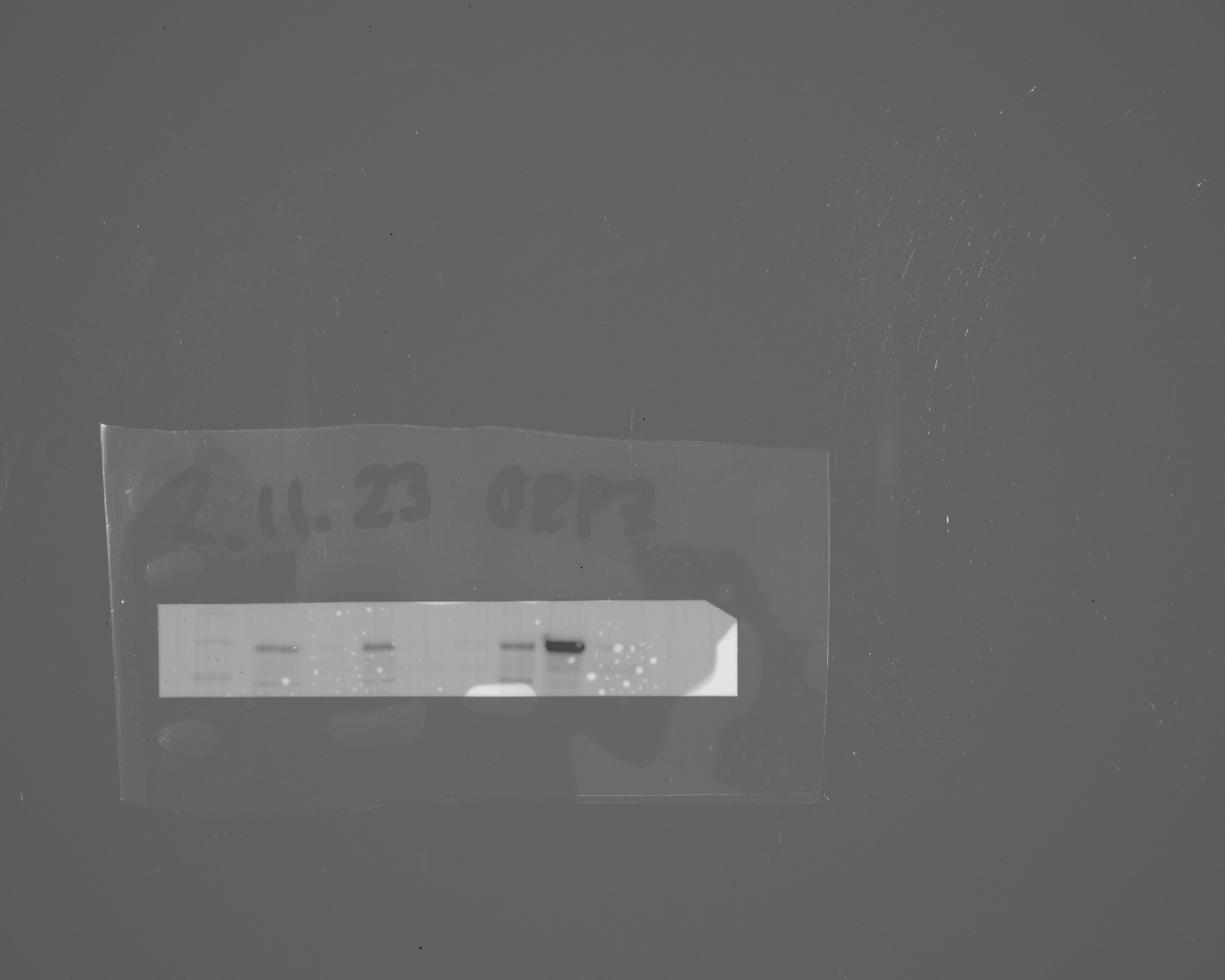


Figure S22. Full membrane image of ORP7 loadings in oex samples used to make Figure 12


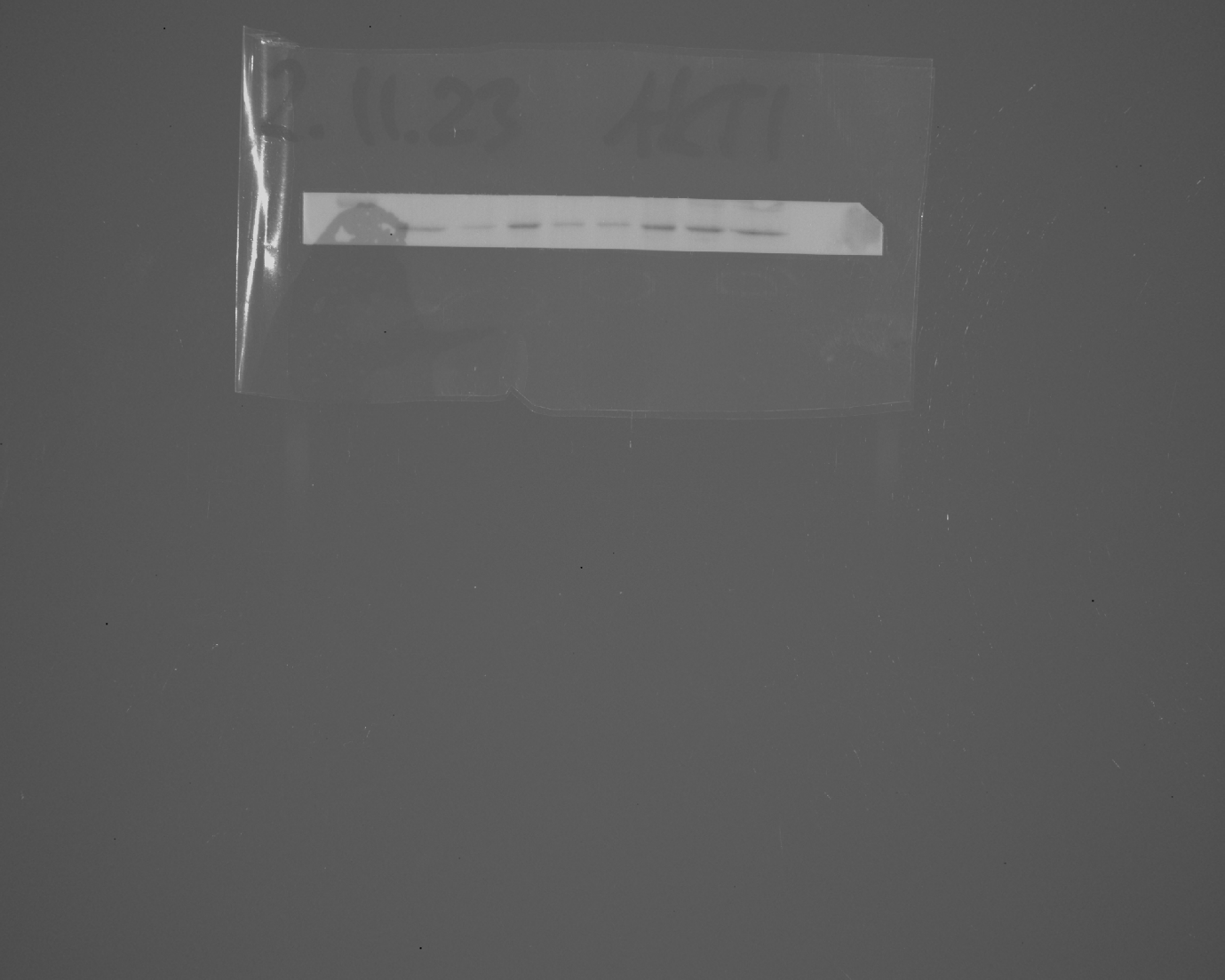


Figure S23. Full membrane image of AKT1 loadings in oex samples used to make Figure 12


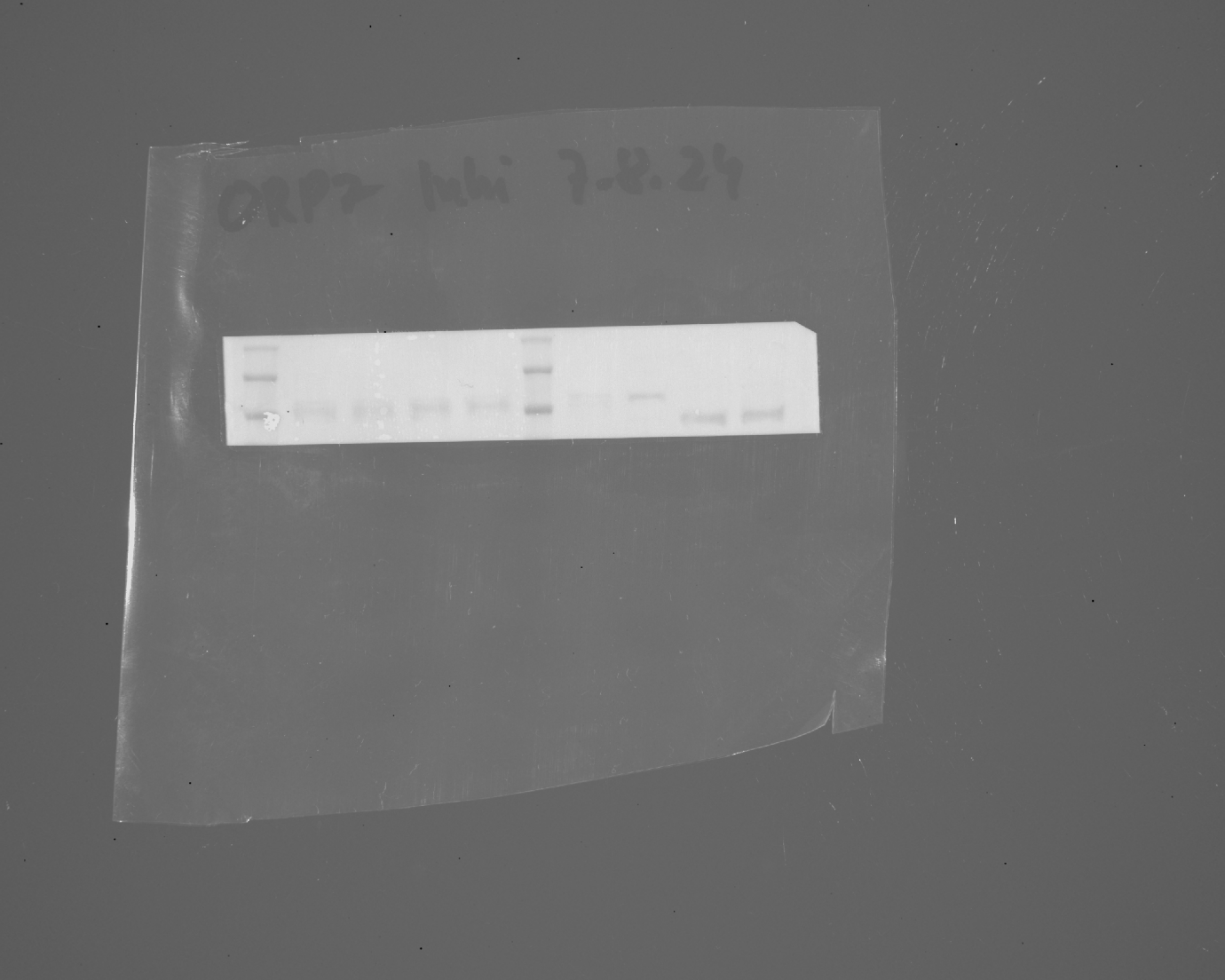


Figure S24. Full membrane image of ORP7 in treated samples used to make Figure S13


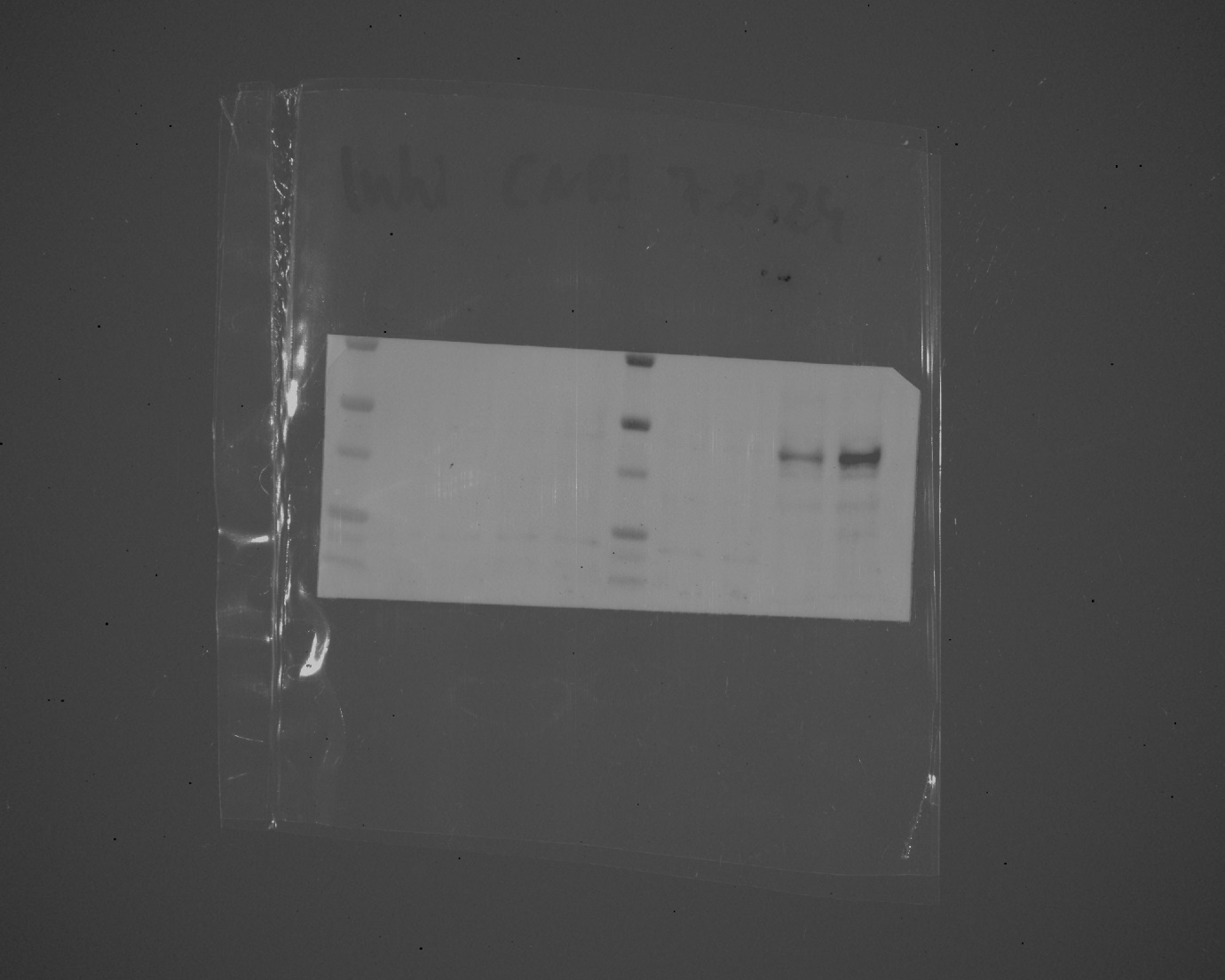


Figure S25. Full membrane image of CNR1 in treated samples used to make Figure S13


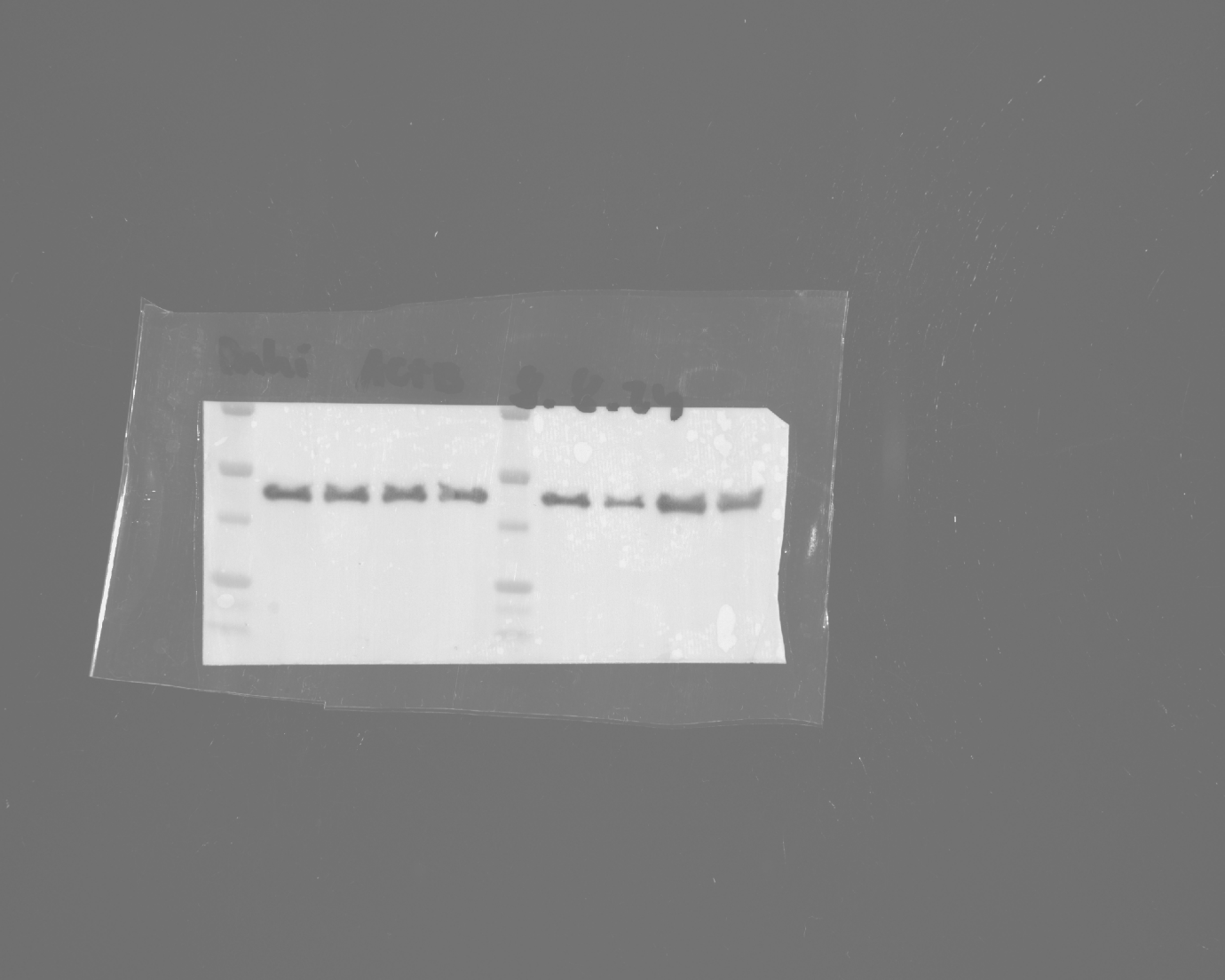


Figure S26. Full membrane image of ACTB in treated samples used to make Figure S13
